# Neurological scoring and gait kinematics to assess functional outcome in an ovine model of ischaemic stroke

**DOI:** 10.3389/fneur.2023.1071794

**Published:** 2023-02-20

**Authors:** Annabel J. Sorby-Adams, Oana C. Marian, Isabella M. Bilecki, Levi E. Elms, Jonathan Camargo, Kelly Hall, Robert G. Crowther, Anna V. Leonard, George I. Wadsworth, Joshua H. Spear, Renée J. Turner, Claire F. Jones

**Affiliations:** ^1^School of Biomedicine, Faculty of Health and Medical Sciences, The University of Adelaide, Adelaide, SA, Australia; ^2^George W. Woodruff School of Mechanical Engineering, Georgia Institute of Technology, Atlanta, GA, United States; ^3^School of Public Health, Faculty of Health and Medical Sciences, The University of Adelaide, Adelaide, SA, Australia; ^4^Alliance for Research in Exercise, Nutrition and Activity (ARENA), University of South Australia, Adelaide, SA, Australia; ^5^School of Mechanical Engineering, Faculty of Sciences, Engineering and Technology, The University of Adelaide, Adelaide, SA, Australia; ^6^Adelaide Spinal Research Group, Centre for Orthopaedics and Trauma Research, The University of Adelaide, North Terrace, SA, Australia; ^7^Department of Orthopaedics and Trauma, Royal Adelaide Hospital, Adelaide, SA, Australia

**Keywords:** stroke, functional outcome, sheep, motion capture, gait, neurological score

## Abstract

**Background:**

Assessment of functional impairment following ischaemic stroke is essential to determine outcome and efficacy of intervention in both clinical patients and pre-clinical models. Although paradigms are well described for rodents, comparable methods for large animals, such as sheep, remain limited. This study aimed to develop methods to assess function in an ovine model of ischaemic stroke using composite neurological scoring and gait kinematics from motion capture.

**Methods:**

Merino sheep (*n* = 26) were anaesthetised and subjected to 2 hours middle cerebral artery occlusion. Animals underwent functional assessment at baseline (8-, 5-, and 1-day pre-stroke), and 3 days post-stroke. Neurological scoring was carried out to determine changes in neurological status. Ten infrared cameras measured the trajectories of 42 retro-reflective markers for calculation of gait kinematics. Magnetic resonance imaging (MRI) was performed at 3 days post-stroke to determine infarct volume. Intraclass Correlation Coefficients (ICC's) were used to assess the repeatability of neurological scoring and gait kinematics across baseline trials. The average of all baselines was used to compare changes in neurological scoring and kinematics at 3 days post-stroke. A principal component analysis (PCA) was performed to determine the relationship between neurological score, gait kinematics, and infarct volume post-stroke.

**Results:**

Neurological scoring was moderately repeatable across baseline trials (ICC > 0.50) and detected marked impairment post-stroke (*p* < 0.05). Baseline gait measures showed moderate to good repeatability for the majority of assessed variables (ICC > 0.50). Following stroke, kinematic measures indicative of stroke deficit were detected including an increase in stance and stride duration (*p* < 0.05). MRI demonstrated infarction involving the cortex and/or thalamus (median 2.7 cm^3^, IQR 1.4 to 11.9). PCA produced two components, although association between variables was inconclusive.

**Conclusion:**

This study developed repeatable methods to assess function in sheep using composite scoring and gait kinematics, allowing for the evaluation of deficit 3 days post-stroke. Despite utility of each method independently, there was poor association observed between gait kinematics, composite scoring, and infarct volume on PCA. This suggests that each of these measures has discreet utility for the assessment of stroke deficit, and that multimodal approaches are necessary to comprehensively characterise functional impairment.

## 1. Introduction

Ischaemic stroke is a leading cause of death and neurological disability worldwide (1, 2). New approaches to reperfusion have extended the previously narrow window for intervention (3, 4), resulting in reduced mortality, yet a higher incidence of patients facing persistent neurological impairment (5). To improve functional outcomes in the increasing number of patients who survive stroke, new therapies targeting secondary injury and neurological recovery mechanisms are urgently required (6). Animal models are an essential step in the development of novel stroke therapeutic agents, with restoration of function a key indicator of a treatment's efficacy. Despite this, the translation of pre-clinical findings to clinically efficacious stroke therapies has been largely ineffective to date (7). This may be a consequence of pre-clinical experimental design, selection of model species, and lack of comprehensive functional assessment (8). Due to their potential for enhanced clinical translation, large animal species, including sheep, pigs and non-human primates (NHP's) are increasingly being used as a screening tool once initial therapeutic efficacy has been demonstrated in small animals (9–12), with accurate assessment of functional deficit in these species necessary for relevance to clinical disability.

Clinical assessment of post-stroke function is often carried out using composite scoring systems such as the National Institutes of Health Stroke Scale (NIHSS) or the modified Rankin Scale (mRS) which are used to determine acute stroke severity and long-term stroke outcomes respectively (13, 14). The NIHSS assesses 11 criteria including vision, facial movement, motor function of the lower and upper extremities, and language disturbances, where a higher score allocated indicates greater stroke severity, providing useful information in the acute care setting. By comparison, the mRS comprises a 7-point scale which assesses functional independence and gait stability ranging from no symptoms to severe disability and death. Although relatively crude, the mRS is commonly employed as a long-term outcome measure in stroke clinical trials. Quantitative differences in gait kinematics have also served as a means of determining asymmetry and extent of neurological motor deficit, with studies demonstrating a significant increase in swing duration, and decrease in gait speed and stride length following stroke onset (15, 16).

Comparable assessment of functional deficits in animal stroke models vary depending on the species. In rodents, composite scores such as the Bederson scale and Modified Neurological Severity Score (mNSS), motor function tests such as rotarod, cylinder test, ledged beam, grid walking, reaching chamber and staircase test, and quantitative systems to assess gait such as the Catwalk and DigiGait (17) are frequently employed. Large animal NHP models utilise the NHP Stroke Scale (NHPSS), which assesses level of consciousness, defence/startle reactions, upper/lower extremity movement, gait, circling, bradykinesia, balance, neglect, visual field, facial weakness, and grasp reflex (18). Additionally, the 2 tube choice test (hand preference, spatial neglect), the hill and valley staircase test (hemiparesis) and the Kluver board (motor control and planning) are common outcome measures used in NHP stroke models to evaluate the severity of post-stroke deficits (19). Although the ability to assess grasp in NHP's is of particular relevance given their comparable dexterity to humans (20–22), strict housing requirements, ethical considerations, and overall expense can limit use in large scale studies (10, 11, 23, 24). Pigs have also been used as a large species to study stroke, with open field [exploratory behaviour; (25)] and gait analysis [step length, step velocity, swing duration, stance duration and maximum hoof height; (26)] described to assess post-stroke deficits. However, some porcine species are not available internationally, such as the Yucatan minipig, limiting widespread use.

The relative availability, amenable nature, and gyrencephalic cerebral structure has led to the increased use of sheep as a species to model stroke (27–31). In addition, the high proportion of white matter within the sheep brain (27.7%) is much closer to that in humans (40–45%) compared with rodent species (10–20%): an important consideration given the vulnerability of white matter to ischaemic injury (24). Functional deficits have been documented following ovine middle cerebral artery occlusion (MCAo), including noticeable hemiplegia of the contralateral limbs and general apathetic behaviour (27, 30). These deficits are comparable to those seen clinically, where malignant MCAo often presents as unilateral hemiplegia and hemiparesis, and resultant compensatory reliance on unaffected ipsilateral limbs (32, 33). Functional assessment in sheep; however, presents unique challenges. Firstly, although composite scoring systems for hooved animals do exist, clinical tasks such as grasp reflex cannot be assessed. Secondly, motor function tests developed for rodents are often difficult to translate to large animals due to the need for increased size of test and measurement apparatus, whilst others are completely inappropriate for large species (e.g., rotarod). Thirdly, although quantitative systems to assess gait kinematics have been reported in ovine musculoskeletal, orthopaedic, and spinal cord injury models (34–37), no assessment for stroke has been described to date.

Developing functional assessment methods that overcome these challenges is key in enabling detection of acute and long-term functional changes post ovine stroke. As such, this study sought to establish a neurological composite scoring system and subsequently develop a method to assess gait kinematics using motion capture in an ovine model of ischaemic stroke. Specifically, this study aimed to: (1) develop a neurological composite scoring system and assess its repeatability in healthy animals pre-stroke; (2) develop a method to assess gait kinematics using motion capture and assess repeatability in healthy animals pre-stroke; (3) determine if a change in neurological composite scoring was detected 3-days post-stroke; (4) determine if a change in gait kinematics was detected 3-days post stroke and (5) determine the relationship between functional outcomes obtained *via* neurological composite scoring, gait kinematics, and infarct volume quantified *via* magnetic resonance imaging (MRI) at 3-days post-stroke.

## 2. Materials and methods

### 2.1. Ethics

This study was approved by the South Australian Health and Medical Research Institute (SAHMRI) Animal Ethics Committee (SAM 3) and conducted in accordance with the Australian National Health and Medical Research Council code of care and use of animals for scientific purposes (8^th^ Edition, 2013), and Animal Research Reporting of *In vivo* Experiments (ARRIVE) guidelines (38). A total of 26 adult Merino sheep (*Ovis aries*, 57 ± 4 kgs, 18–36 months), obtained from a single farm (Gum Creek, South Australia) were used (*n* = 13F; 13M). Six months prior to commencing the study, animals were moved from the farm to the research facility [SAHMRI Preclinical Imaging and Research Facility (PIRL)], where on arrival they were examined by a veterinarian and judged to be healthy prior to study inclusion based on complete physical and orthopaedic examinations. Sheep were treated prophylactically with antiparasitic ivermectin administered intramuscularly (0.25 mg/kg, Ivomec 0.8 g/L) and cydectin administered by oral drench (0.1% Moxidectin). Animals were fed once daily with a combination of feedlot, nuts, grain, and lucerne hay (Laucke Mills, South Australia), with free access to water.

### 2.2. Experimental design

**Figure 1:**
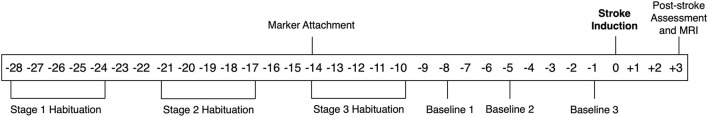
Experimental timeline. Animals arrived at the facility 6 months prior to stroke induction. Thereafter procedures commenced 4 weeks prior to stroke (where stroke induction is indicated as day 0) including staged habituation, palpation and marker attachment, baseline assessments, post-stroke assessment, and MRI.

To determine the repeatability of neurological composite scoring and gait kinematics, pre-stroke, baseline assessment was carried out on three occasions; 8-, 5- and 1-day prior to stroke induction. To compare pre- and post-stroke parameters, assessment was performed 3 days following stroke onset. MRI was carried out at 3 days post-stroke following completion of functional assessments. Experimental timeline and procedures are shown in Figure 1.

### 2.3. Neurological composite scoring

A 10 criteria neurological assessment score was adapted from previous work (27) based on the common system for neurologic dysfunctions in large animals (39). This study specifically focused on the functional deficits observed in animals following transient MCAo, including changes in demeanour, behaviour, and motor dysfunction (Table 1). A score of 0 was considered normal, with a possible total score of 36 indicating severe deficit.

**Table 1:** Neurological scoring system.

**Combined scores**	**Criterion**	**Assessment**	**Score**
**Demeanour**	**1**	**State of activity/consciousness**	**0 – 3**
		Normal	0
		Apathetic	1
		Stupor	2
		Comatose	3
**Behaviour**	**2**	**Presence of food debris in the mouth**	**0 – 1**
		No food	0
		Food present	1
	**3**	**Presence of torticollis**	**0 – 1**
		Absent	0
		Present	1
	**4**	**Partial flexion of fetlock and/or carpus**	**0 – 1**
		Absent	0
		Present	1
	**5**	**Ataxia/Dysmetria**	**0 – 3**
		Normal	0
		Dysmetric limb movement	1
		Staggering	2
		Animal falls down	3
	**6**	**Circling movements**	**0 – 2**
		None	0
		Occasional	1
		Continuous	2
**Left/Right postural reactions**	**7**	**Hemi-standing**	**0 – 4**
		Immediate adjustment of both limbs	0
		Delayed adjustment of hindlimb	1
		Delayed adjustment of forelimb	2
		Delayed adjustment of both limbs	3
		No adjustment of both limbs	4
		Knuckling limb on hoof release	+ 0.25/limb
	**8**	**Forelimb lateral and medial hopping**	**0 – 2**
		Normal response/immediate adjustment	0
		Delayed adjustment	1
		No adjustment	2
		Knuckling limb on hoof release	+ 0.25/limb
	**9**	**Limb medial adjustment “lateral dragging”**	**0 – 2**
		Normal response/immediate adjustment	0
		Delayed adjustment	1
		No adjustment	2
		Partial correction	+ 0.25/limb
		Limb drag on return	+ 0.25/limb
**Wheelbarrowing**	**10**	**Forced forelimb movement “wheelbarrowing”**	**0 – 2**
		Normal response/immediate adjustment	0
		Drifting to side	1
		Animal falls down	2

Each criterion was scored at the time of assessment upon agreement of two independent assessors. Observations of level of consciousness and state of activity gave a score for animal demeanour (Table 1, criterion 1). Animals who were comatose warranted euthanasia, and no further investigations were performed. Abnormalities in animal behaviour were assessed by cumulative scores for presence of food debris in the mouth indicating inability to properly masticate, torticollis, evidence of abnormal flexion at the fetlock and/or carpus/tarsus joints, general ataxia or dysmetria in limb movements, and circling (Table 1, criteria 2, 3, 4, 5, and 6 respectively). Circling behaviours (criterion 6) were monitored prior to animal handling on assessment days by undisturbed video recording of the animal for 10 min within their home pen environment.

Three postural reaction tests (Table 1, criteria 7, 8 and 9) were conducted by forcefully shifting the animal's weight over their centre of gravity on individual limbs and assessing their ability to correct the movement. Criterion 7 refers to “hemi-standing”, which evaluated the animal's ability to correct and co-ordinate fore- and hind-limbs during a lateral movement on the left and right side of the body. Criterion 8 refers to the “hopping reaction” which assessed forelimbs individually to determine the animal's ability to correct the limb during lateral movement. Additional quarter scores were allocated in criteria 7 and 8 if the animal exhibited inability to fully extend a limb upon release, causing ‘knuckling' on the ground. Criterion 9 encompassed “lateral dragging”, which involved the forced lateral movement of each individual limb and assessment of the animal's ability to return the limb back to the medial starting position. Quarter scores were given for criterion 9 if animals dragged a limb on return (0.25/limb) or if correction back to original position was only partial (0.25/limb). Scores for hemi-standing, hopping, and lateral drag were incorporated into a single postural reaction measure for the contralateral and ipsilateral side of the body, respectively. Forced forward movement of the animal on both forelimbs (“wheelbarrowing”, Table 1, criterion 10) assessed for any sideways deviation, indicative of hemineglect and potential hemiparesis, which was reported independently. All scoring took place approximately 2 h prior to commencing motion capture procedures.

### 2.4. Motion capture of gait kinematics

#### 2.4.1. System design and hardware

The relative size and strength of sheep requires the construction of robust systems that are adaptable for use in farming environments yet enable safe handling throughout assessment to ensure both animal and handler wellbeing. Given these requirements, a fenced, motion capture run measuring 10 × 5 × 1 m was fabricated using standard building and farming equipment (Supplementary Figure S1). One length of the run was defined as the capture volume (the space in which cameras can detect movement of the animal), with the remaining providing a circular pathway back to the capture volume. Sheep were encouraged to walk forwards through the run, with a familiar researcher walking behind them at a consistent pace. As the sheep turned the corners of the run, the researcher appeared in their visual field along the edge of their flight zone (40–42), encouraging continuous forward movement.

Ten motion capture cameras (Vicon Vero, Vicon Motion System Ltd., Oxford, UK) were placed equidistant around the periphery of the capture area, five on either side, approximately 1 m from the fence line and at a height of 1–1.5 m. Vicon Nexus software (v2.10) was used to capture marker data at a frame rate of 200 Hz. An additional video camera (Vicon Vue, Vicon, Oxford, UK) operating at a frame rate of 60 Hz captured video footage which was superimposed to the motion capture data for quality control when post-processing.

#### 2.4.2. Habituation

Staged habituation was undertaken prior to assessment to familiarise animals with handling and testing procedures. On facility arrival, animals were initially housed in protected outdoor pens in groups of six. Four weeks prior to surgery, animals were separated into pairs in the same outdoor pens. During this time, they underwent a three-stage habituation protocol (Figure 1): Stage 1: pairs of animals (housed together) were allowed to roam the functional run without a handler for 30 min on five consecutive days; Stage 2: individual animals traversed the run in a clockwise direction (30 min for five consecutive days), with a handler walking behind them to encourage forward movement; Stage 3: animals were trained to step into, and out of, a modified transport crate; grain (Laucke Mills, South Australia) was used to encourage animals to step into the crate without handler intervention. Habituation and testing procedures were carried out by four trained handlers familiar to the sheep.

#### 2.4.3. Anatomical landmarks

**Figure 2:**
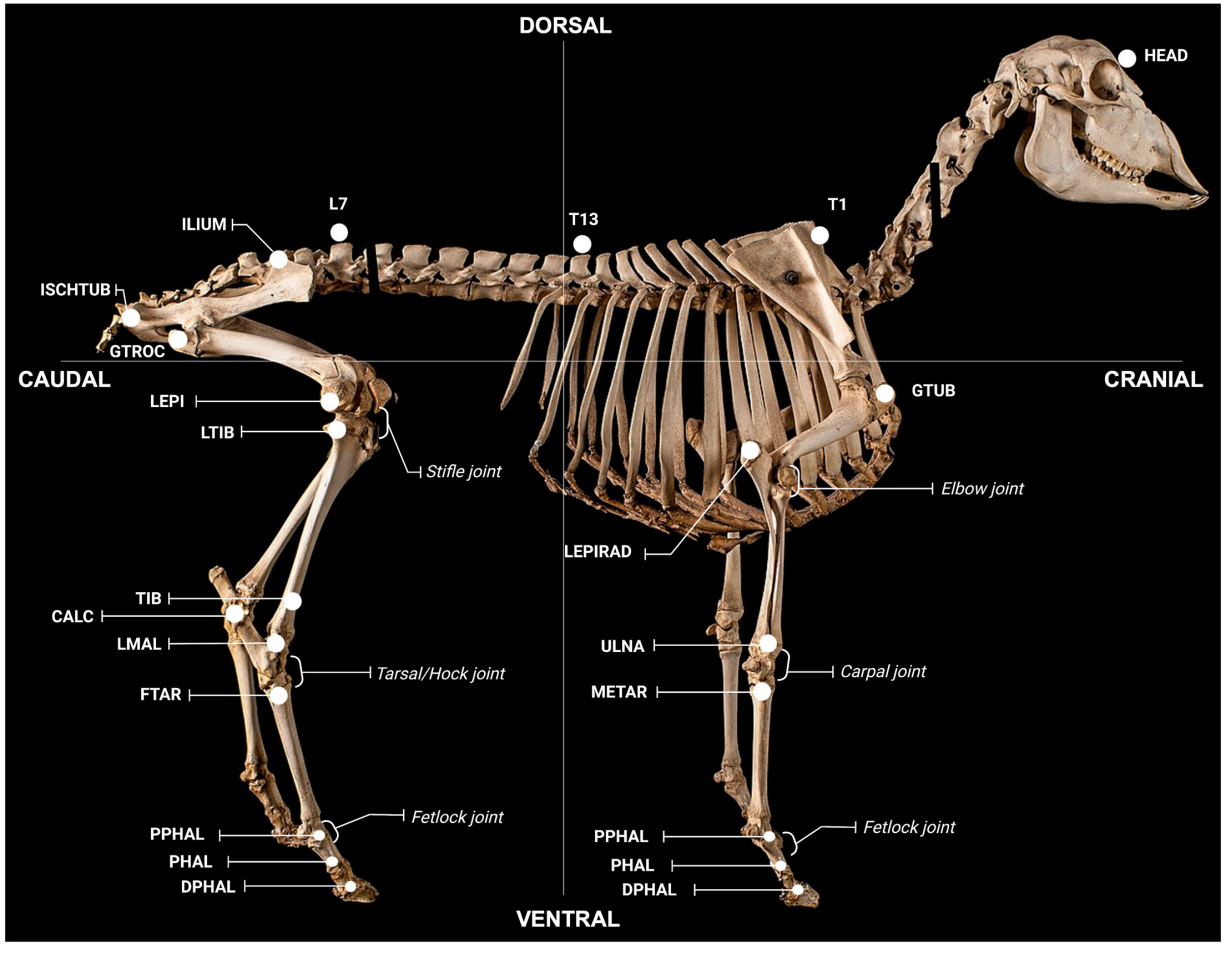
Anatomical locations for gait assessment. Locations were defined as follows; *global*: HEAD (on the head between the eyes), T1 (spinous process of T1), T13 (spinous process of T13), L7 (spinous process of L7); *forelimb*: GTUB, greater tubercle of the humerus; LEPIRAD, lateral epicondyle of the radius; ULNA, distal tubercule of the ulna; METAR, proximal tubercule of the metatarsal; PPHAL, forelimb proximal phalange; PHAL, forelimb phalange; DPHAL, forelimb distal phalange; *hindlimb*: ILIUM, iliac crest; ISCHTUB, ischial tuberosity; GTROC, greater trochanter of the femur; LEPI, lateral epicondyle of the femur; LTIB, lateral condyle of the tibia; TIB, tibia; CALC, calcaneus; LMAL, lateral malleolus; FTAR, fused tarsal of the metacarpus; PPHAL, hindlimb proximal phalange; PHAL, hindlimb phalange; DPHAL, hindlimb distal phalange. The METAR/FTAR, PPHAL, and PHAL were used to determine the fetlock angle of the fore and hind limbs respectively. The LEPIRAD, ULNA, METAR, and PPHAL and the TIB, LMAL, FTAR and PPHAL, were used to determine the angle of the carpus and tarsus, respectively. The GTUB, LEPIRAD, and ULNA and the GTROC, LEPI, LTIB, and LMAL were used to determine the angle of the elbow and stifle, respectively. Retro-reflective markers attached to each location were 15 mm in diameter, except for markers on the fore- and hind-limb PHAL, PHAL and DPHAL, which were 9 mm. Figure adapted from a sheep skeleton on display at the Museum of Veterinary Anatomy, Faculty of Veterinary Medicine and Animal Science, University of São Paulo, Brazil.

Spherical retro-reflective markers (9- and 15-mm diameter; B&L Engineering, California, USA) were non-invasively attached to 42 anatomical landmarks (Figure 2) using hooked Velcro^®^ (Velcro USA Inc, Manchester, NH, US). The opposing loop surface of the Velcro^®^ was adhered to the animal using cyanoacrylate adhesive (Bostik, Australia). To ensure consistent marker placing, 6 days prior to baseline testing, animals were intubated and anaesthetised (1.5% isoflurane, Henry Schein, Australia), anatomical locations palpated, and landmarks tattooed using a handheld tattoo gun and India ink (Windsor and Newton, Australia). Animals were shorn weekly to facilitate marker reattachment and visualisation.

#### 2.4.4. Motion capture data collection

On testing days, the Vicon motion capture system was calibrated, and the global coordinate system (GCS) set using a light emitting diode wand (Vicon Active Wand, Vicon Motion System Ltd., Oxford, UK). The GCS z-axis corresponded to the vertical (sagittal) direction with the positive axis pointing up; the y-axis corresponded to the direction of the progression (forward); and the x-axis corresponded to the lateral direction of the animal (left/right) with the positive axis pointing to the right side. Prior to assessment, animals were placed into a modified transport crate, reflective markers attached to anatomical locations, and moved to the functional testing space where they completed a minimum of 20 traverses of the functional run. Once motion capture was complete, reflective markers were removed and animals were returned to their home pen.

#### 2.4.5. Motion capture data post-processing

Post-processing of motion capture data was performed using Vicon Nexus software (version 2.10, Vicon Motion System Ltd., Oxford, UK) with 5 trials in which the animal maintained a consistent walking pace reconstructed for each session (8-, 5-, and 1-days pre-stroke and 3 days post-stroke). Markers were labelled and spline and cyclic algorithms used to fill all visible gaps (Vicon Motion System Ltd., Oxford, UK). A fourth order, zero lag, low pass Butterworth filter was applied with a cut-off frequency of 10 Hz. Data were exported to C3D format and further processed with custom MATLAB^®^ code (Mathworks, Natick, MA, USA).

Motion capture parameters selected for analysis sought to capture post-stroke gait, asymmetry and general apathetic behaviour observed, such as lowering of the head and shoulders and inability to extend the fetlock joint contralateral to the stroke affected hemisphere. Parameters of interest were subsequently classified into global and limb-specific. The final parameters selected for analysis and their purpose are provided in Supplementary Table S1 (global parameters) and Supplementary Table S2 (limb-specific parameters). Global parameters correspond to the outcome measures pertaining the entire trial, for example forward velocity. These outcomes were calculated as the mean value across the entire trial. Limb-specific parameters were computed from the observation of the kinematic data of each limb within its corresponding gait cycle. For each trial, the gait cycles were identified following the method from Ghoussayni et al. (43). Changes in the velocity of each limb's hoof marker (DPHAL, Figure 2) were detected in the vertical and progression directions, determining when the marker stopped moving (entering stance phase) or started moving (entering swing phase). One complete gait cycle per limb was extracted from each trial to calculate kinematic measures of interest using two-dimensional (2D) planar analysis. Planar analysis was independently performed in the vertical (sagittal) and lateral (left/right) directions. Joint angles were defined between two vectors in the sagittal plane for the fetlock, carpus, and elbow of the forelimb, and fetlock, tarsus, and stifle of the hindlimb (Figure 2).

### 2.5. Surgical procedures

#### 2.5.1. Preoperative preparation

Twelve hours prior to surgery, animals were moved to indoor pens and fasted. Anaesthesia was induced with intravenous ketamine (0.05 mL/kg, 100 mg/kg Injection, CEVA, Australia) and diazepam (0.08 mL/kg, 5 mg/mL injection, Pamlin, CEVA, Australia). A jugular catheter (18 g, Terumo SURFLO^®^) was inserted for delivery of intraoperative crystalloid fluids (Hartmann's, Baxter Health, Australia). Anaesthesia was maintained with inhaled isoflurane (1.5–2.0% in 3 L of air and 500 mL of oxygen, Henry Schein, Australia) and continuous ketamine infusion (4 mg/kg/hr) *via* the jugular catheter. An arterial catheter (20 g, Terumo SURFLO^®^) was placed in the distal hindlimb to yield arterial blood samples for blood gas analyses. A paediatric blood pressure cuff (Easy Care Cuff, Phillips) was placed on the proximal forelimb for a non-invasive measure of arterial blood pressure which was manually recorded at 5-minute intervals.

#### 2.5.2. Intraoperative procedures

Stroke surgery was performed as previously described in detail (30). Due to the presence of a rete mirabile in sheep, endovascular methods are precluded and direct access to the cerebrovasculature is required [for details and review please see (10, 44–46)]. To achieve this, an incision was made between the right ear and orbital rim, coronoid process of the mandible lateralised, and skull base exposed to perform a small craniotomy using a pneumatic drill (Midas Rex^®^ Legend Electric System, Medtronic USA). A 2 cm skull flap was removed, underlying dura breached, proximal middle cerebral artery (MCA) located, and an aneurysm clip (Aesculap YASARGIL^®^ Aneurysm Clip, Germany) placed over the vessel which remained *in situ* for 2 h. The clip was subsequently removed to achieve reperfusion, dura closed watertight with synthetic matrix (Durepair^®^, Medtronic, USA) and cyanoacrylate adhesive (Bostik, Australia), cranioplasty performed using dental cement (Sledgehammer, Keystone, Germany), and surgical site closed in layers using polyglactin suture (Vicryl^®^, ETHICON). Arterial blood samples were obtained at hourly intervals intra-operatively to maintain the animal within normal physiological limits.

#### 2.5.3. Postoperative recovery

Animals were removed from anaesthesia and, once lucid, treated with subcutaneous non-steroidal anti-inflammatory (NSAID, 0.7 mg/kg, 50 mg/mL every 12 h, Carprofen, Norbrook, Australia) and intramuscular Buprenorphine (Temgesic, 1.0 mL, 324 μg/mL Buprenorphine hydrochloride, Reckitt Benckiser, Australia) for pain relief, and intramuscular Depocillin for antibiosis (1 mL/25 kg every 12 h, Procaine benzylpenicillin, Intervet, Australia). NSAID and antibiotic treatment was continued for 3 days post-operatively, and as required thereafter. Clinical assessment was carried out twice daily to determine animal wellbeing, including urine and faecal output, food and water intake, and signs of apathy. Animals remained in indoor housing for 3 days post-operatively, after which they were returned to protected outdoor pens and housed individually.

### 2.6. Magnetic resonance imaging

Twenty (*n* = 10M; 10F) of the 26 animals underwent MRI at 3 days post-stroke under general anaesthesia (1.5% isoflurane, Henry Schein, Australia) on a 3 Tesla (T) Siemens Magnetom (Siemens AG, Munich, Germany) using a posterior 20-channel head coil enabling collection of T2 fluid attenuated inversion recovery (FLAIR) and diffusion weighted images (DWI). Axial T2 FLAIR sequences were acquired with a slice thickness of 0.89 mm, repetition time (TR) 5000 ms, echo time (TE) 386 ms, 1 average, flip angle of 12°, acquisition matrix of 256 × 256, and in plane resolution of 0.39 mm/pixel. DWI sequences were acquired with a slice thickness of 3 mm, TR 5600 ms, TE 80 ms, 1 average, flip angle of 180°, acquisition matrix of 190 × 190, in plane resolution of 0.85 mm/pixel, with 4 diffusion directions and *b*-values of 0 and 1000 s/mm^2^. Semi-automated segmentation of *b*-1000 DWI data was performed using ITK-SNAP (v3.8) to estimate infarct volume as previously described (47). Midline shift was calculated using axial T2 FLAIR images on RadiAnt (v2020.2). The midline between the left and right hemisphere was defined at the level of the foramen Monro and the degree of shift measured perpendicular to the midline in mm where the septum pellucidum was most displaced. Three measurements were recorded at intervals (one at the level of the foramen of Monro, one 4 mm superior, and one 4 mm inferior) and subsequently averaged to provide a single value in mm.

### 2.7. Euthanasia

At 28 days post-stroke, animals were euthanised *via* exsanguination under general anaesthesia (2% isoflurane, Henry Schein, Australia) and common carotid perfusion with tris-buffered saline (Sigma-Aldrich, Australia) following intravenous heparin administration (5000 IU/5 mL, Pfizer, NY).

### 2.8. Statistics

Statistical analysis was performed using Stata (version 17.0, StataCorp, College Station, TX). Normality was assessed *via* visual inspection of histograms. Normally distributed continuous variables are reported as mean and standard deviation (SD) and analysed using parametric modelling. Skewed data are reported as median and interquartile range (IQR) and analysed using non-parametric tests.

#### 2.8.1. Baseline repeatability analysis

Intraclass Correlation Coefficients (ICC) were used to describe repeatability across the three baseline sessions (8-, 5-, and 1-day pre-stroke). For neurological composite scoring, ICC estimates were based on a mean-rating (*k* = 3), absolute agreement, two-way mixed effects model with non-parametric bootstrapped 95% confidence intervals (CI). Differences between baseline testing days was analysed using a Kruskal-Wallis test.

For gait kinematics, ICC's were based on a mean-rating (*k* = 3), absolute agreement, two-way mixed-effects model with parametric 95% CI. For limb-specific measures, two ICC values were derived; one un-adjusted and one adjusted for the potential confounding effect of walking speed (measured as mean absolute velocity at T1) using linear regression analyses. One-way repeated measures analysis of variance (ANOVA) were used to determine if there was a difference between the three baseline measures for each kinematic variable.

#### 2.8.2. Post-stroke analysis

To determine differences in neurological scoring pre- vs. post-stroke, the mean across all baseline trials for each criterion was calculated to provide a single value. The mean baseline value was subsequently compared with 3 days post-stroke using a Mann–Whitney *U*-test.

To determine the change in gait kinematics pre- vs. post-stroke, a single baseline measure comprised of the mean of the three baselines was also calculated for each variable of interest. Linear mixed models (LMMs) were used to determine differences post-stroke. A random effect of sheep was used to account for the correlation between repeated or multiple measures on the same animal. Fixed effects were time (pre-, post-stroke), limb (left, right), and a time-by-limb interaction term. The interaction term was necessary as the right sided stroke was expected to cause left-sided deficits (with potential right-sided compensation) thus producing a side-dependent effect of time. Two models were fitted for each limb-specific measurement; the first un-adjusted and the second adjusted for velocity. Estimates of the difference between baseline and post-stroke were derived for each limb.

#### 2.8.3. Gender analysis

The effect of gender was assessed pre- and post-stroke for the following variables: infarct volume on DWI (post-stroke only), total neurological score, kinematic global measures including; mean absolute velocity and mean head to T1, and limb specific measures for the fore- and hind- limbs (both left and right) including minimum, maximum and range of the fetlock in stance, minimum, maximum and range of the fetlock in swing, and duration of stance, swing, and stride. Pre-stroke comparisons used the mean of all baseline measures. Infarct volume and neurological score were analysed using a Mann-Whitney *U*-test. Kinematic global measures were analysed using linear regression modelling. Limb specific measures were analysed using LMMs with fixed effects for gender and leg and random effect for animal. All models were adjusted for velocity.

#### 2.8.4. Principal component analysis

To determine the relationship between gait kinematics, total neurological examination score, and infarct volume at 3 days post-stroke, a Principal Components Analysis (PCA) was performed. The measures considered for inclusion in the PCA were infarct volume on DWI, total neurological score, kinematic global measures including; mean absolute velocity and mean head to T1, and limb specific measures for the forelimbs (both left and right) including minimum, maximum and range of the fetlock in stance, minimum, maximum and range of the fetlock in swing, and duration of stance, swing, and stride. The number of extracted components for analysis was based on eigenvalues >1, and inspection of scree plots. A correlation matrix was used to assess correlations between variables. Kaiser–Meyer Olkin (KMO) measures of sampling adequacy were used to assess how suitable the data was for PCA, with scores assigned to each variable and the complete model. Individual scores <0.50 implied that the variable was not sufficiently correlated with the other variables to warrant inclusion and was excluded from final analysis. Bartlett's test was used to assess whether the variables, after PCA, presented variable homogeneity.

#### 2.8.5. Statistical interpretation

Results for ICC's are presented as ICC and 95% confidence intervals (CI). ANOVA, Kruskal-Wallis and Mann–Whitney *U*-tests between baseline sessions are presented as *p*-values. Results from LMM and linear regression models are presented as mean difference, 95% CI, and *p*-value. Interpretation of ICC values was <0.50 poor; 0.50 −0.75 moderate; 0.75 −0.90 good; >0.90 excellent (48). A *p* < 0.05 was considered statistically significant throughout.

## 3. Results

Two animals were euthanised prematurely and excluded from the study (intravenous administration of 160 mg/kg sodium pentobarbital, Lethabarb, Australia). One animal had unsuccessful reperfusion of the MCA resulting in a permanent stroke, and the other had kidney failure leading to seizures. Twenty-four animals (*n* = 12M; 12F) reached the experimental endpoint for neurological scoring and gait kinematics and were included in the final analysis. Twenty (*n* = 10M; 10F) of these animals underwent MRI and were subsequently used for the PCA.

### 3.1. Neuroscore assessment

**Figure 3:**
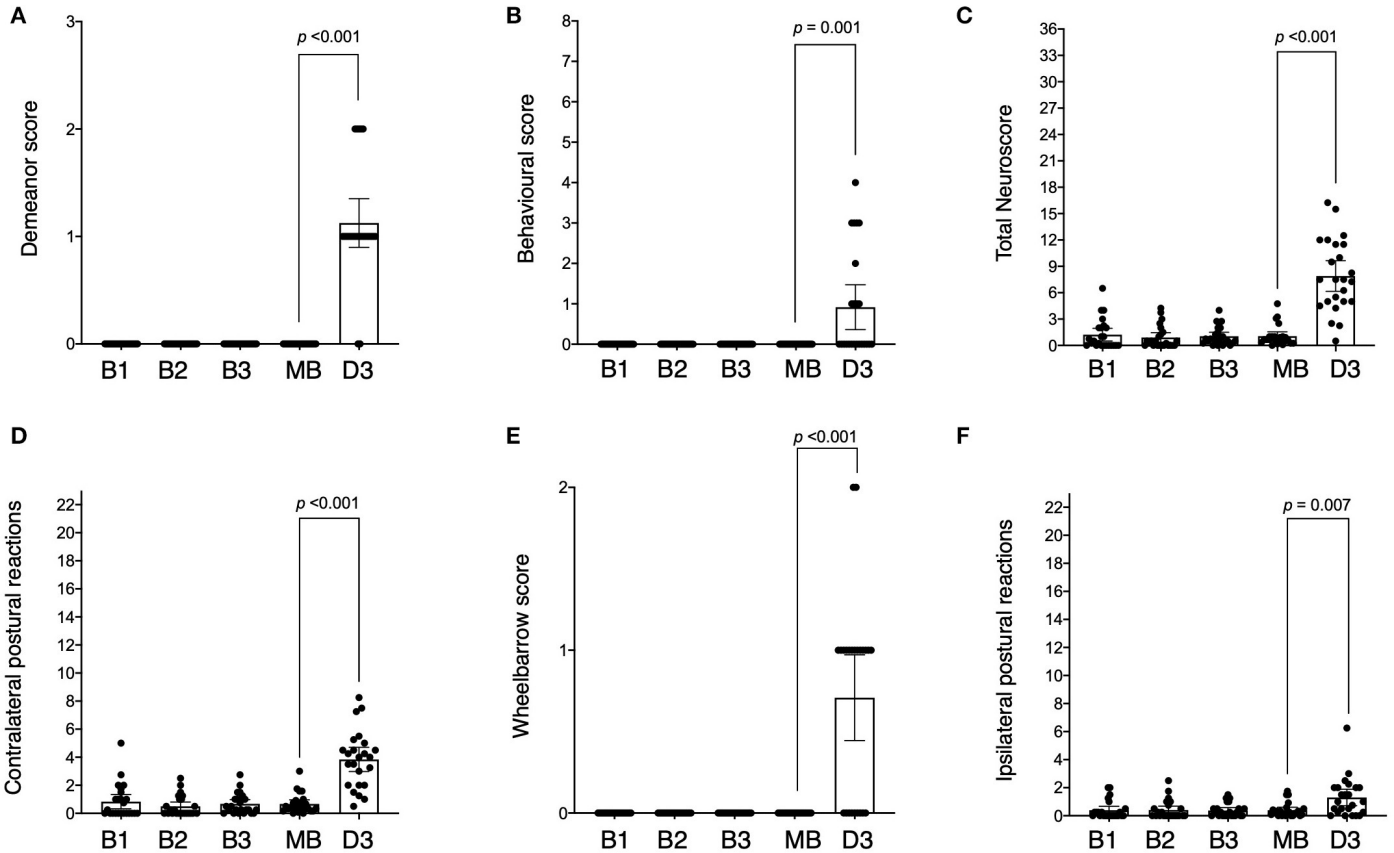
Neurological scores across baseline and post-stroke testing sessions. Sessions are indicated as baseline 1 (B1), baseline 2 (B2), baseline 3 (B3), mean of all the baselines (MB), and day 3 post-stroke (D3). Scores across testing sessions are shown for demeanour [**(A)** score range 0–3], behaviour [**(B)** score range 0–8], ipsilateral postural reactions [**(C)** score range 0–23], contralateral postural reactions [**(D)** score range 0–23], wheelbarrowing [**(E)** score range 0–2] and total neuroscore [**(F)** score range 0–36]. The difference (*p*-value) between mean baseline and day 3 post-stroke is indicated as shown.

All animals had scores of zero across baseline sessions for demeanour (criterion 1, Figure 3A), behaviour (criteria 2, 3, 4, 5, and 6, Figure 3B) and wheelbarrowing (criterion 10, Figure 3E) (all *p* ≥ 0.999), with only minor variations in postural reactions (criteria 7, 8, and 9) observed in both the left [0.25 (IQR 0.00 to 1.00), Figure 3D] and right [0.00 (IQR 0.00 to 0.50), Figure 3C] limbs, although not significant (*p* = 0.408 and *p* = 0.854 respectively). There was no difference in the total neuroscore between baseline trials [0.50 (IQR 0.00 to 2.00), *p* = 0.505, Figure 3F], and repeatability was moderate (ICC = 0.56, 95% CI: 0.22 to 0.90). Repeatability of right sided (ipsilateral) postural reactions was moderate (ICC = 0.59, 95% CI: 0.33 to 0.86), but left sided (contralateral) postural reactions had poor repeatability (ICC = 0.36, 95% CI: 0.07 to 0.66). Due to the results for demeanour and behaviour being consistently zero, no ICC could be calculated for these criteria.

At 3 days post-stroke, the total neuroscore was higher than mean baseline (median difference: 6.83, IQR: 4.21 to 10.08, *p* < 0.001), with post-stroke scores ranging from 0.5 to 16.25 out of a maximum score of 36 (Figure 3F). Twenty-two of the 24 animals displayed evidence of diminished demeanour following stroke (criterion 1) which was higher than that observed pre-stroke (median difference: 1.00, IQR: 1.00 to 1.00, *p* < 0.001, Figure 3A). Behavioural scores incorporating food debris, torticollis, flexion of the fetlock, dysmetria, and circling (criteria 2, 3, 4, 5, and 6 respectively) were also higher post-stroke compared to pre (median difference: 0.00, IQR 0.00 to 1.50, *p* = 0.001, Figure 3B). The postural reaction score (criteria 7, 8 and 9) for the right (ipsilateral) limbs following stroke was higher when compared to baseline (median difference: 0.63, IQR: 0.00 to 1.88, *p* = 0.007, Figure 3C) although this was more marked in the left (contralateral) limbs following stroke (median difference 3.33, IQR: 1.08 to 4.38, *p* < 0.001, Figure 3D). The wheelbarrow score (indicating lateral drifting; observed in 15/24 animals) was also greater post-stroke than pre-stroke (criterion 10, median difference: 1.00, IQR: 0.00 – 1.00, *p* < 0.001, Figure 3E).

### 3.2. Gait kinematics

#### 3.2.1. Baseline repeatability analysis of global parameters

No difference in the means was detected for any of the global gait measures across the three baseline sessions (all *p* ≥ 0.317) (Supplementary Table S3). However, the repeatability of forward velocity was poor (ICC = 0.41, 95% CI: 0.20 to 0.66), with animals walking at variable speeds between sessions (1.28 ± 0.20 s). Repeatability of measures in the vertical axis were good, with the position of the head in relation to T1 (ICC = 0.70, 95% CI: 0.51 to 0.84), T1 to T13 (ICC = 0.74, 95% CI: 0.56 to 0.86), and T13 to L7 (ICC = 0.80, 95% CI: 0.66 to 0.90) consistent across baseline testing sessions. The position of the head to T1 in the left/right axis had moderate repeatability (ICC = 0.58, 95% CI: 0.36 to 0.76), with animals consistently holding their head slightly to the right of the run (approximately 3 cm). Variables with moderate and above repeatability are denoted with an asterix (^*^) in Supplementary Table S3.

#### 3.2.2. Baseline repeatability analysis of limb parameters

There was no difference between baseline trials for all limb-related variables (all *p* ≥ 0.050) with the exception of the range of the hoof height in swing for the left forelimb (*p* = 0.036, 6.73 ± 0.33 m/s). ICC repeatability was good for some, but not all, of the recorded measures (highlighted by an asterix (^*^) in Supplementary Table S4 for the forelimbs and Supplementary Table S5 for the hindlimbs). To allow for comparison with post-stroke trials, the outcome measures for each individual limb are described herein.

For the forelimbs, the majority of outcome measures were repeatable (Supplementary Table S4). For the left forelimb, the variables that had moderate-to-good repeatability included swing duration (ICC = 0.60, 95% CI: 0.39 to 0.78), hoof vertical swing (ICC = 0.64, 95% CI: 0.44 to 0.80), range hoof height (ICC = 0.74, 95% CI: 0.56 to 0.86) and stride length (ICC = 0.73, 95% CI 0.56 to 0.86). Joint angles were also of moderate-to-good repeatability (all ICC ≥ 0.50), except for the minimum fetlock angle in swing (ICC = 0.43, 95% CI: 0.21 to 0.68). When adjusted for velocity, the ICC increased for most variables, and stride duration (which previously had poor repeatability) was subsequently considered moderately repeatable (ICC = 0.59, 95% CI: 0.38 to 0.77). For the right forelimb, all joint angles had moderate-to-good repeatability (all ICC ≥ 0.50), both adjusted and unadjusted for velocity. Swing duration (ICC = 0.53, 95% CI: 0.31 to 0.74), hoof lateral deviation (ICC = 0.51, 95% CI: 0.21 to 0.72), hoof vertical swing (ICC = 0.670, 95% CI: 0.39 to 0.78), and hoof height range (ICC = 0.70, 95% CI: 0.51 to 0.84), had moderate-to-good repeatability, but only after velocity adjustment.

The repeatability of hindlimb parameters was similar to that of the forelimbs (Supplementary Table S5). For the left hindlimb, repeatable variables unadjusted for velocity included swing duration (ICC = 0.54, 95% CI: 0.33 to 0.74), hoof vertical swing (ICC = 0.50, 95% CI: 0.28 to 0.72), hoof height range (ICC = 0.61, 95% CI: 0.40 to 0.79), and stride length (ICC = 0.62, 95% CI: 0.41 to 0.79). All left hindlimb angles had moderate-to-good repeatability (all ICC ≥ 0.50). When adjusted for velocity, the ICC increased for most variables, and hoof forward swing (ICC = 0.66, 95% CI: 0.46 to 0.82) and stride duration (ICC = 0.62, 95% CI: 0.41 to 0.79) became moderately repeatable measures. For the right hindlimb, variables repeatable in the unadjusted model included swing duration (ICC = 0.58, 95% CI: 0.37 to 0.77), hoof vertical swing (ICC = 0.59, 95% CI: 0.37 to 0.78), hoof height range (ICC = 0.61, 95% CI: 0.40 to 0.79), and stride length (ICC = 0.66, 95% CI: 0.47 to 0.82). Joint angles all had moderate-to-good repeatability (all ICC ≥ 0.50), except for the minimum fetlock angle during swing (ICC = 0.39, 95% CI: 0.18 to 0.65). When adjusted for velocity, the ICC for all variables increased (all ICC ≥ 0.50), and both stride duration (ICC = 0.62, 95% CI: 0.41 to 0.79) and hoof forward swing (ICC = 0.68, 95% CI: 0.48 to 0.83) were moderately repeatable. The distance between the matching limbs during stance was also repeatable (moderate-to-good) for the fore- and hind- limbs (ICC ≥ 0.50, only valid as a measurement when unadjusted for velocity).

#### 3.2.3. Baseline vs. post-stroke analysis of global parameters

Following stroke, all global gait parameters were reduced compared to pre-stroke (all *p* ≤ 0.049, Supplementary Table S6). Animals walked more slowly following stroke, reducing the mean forward velocity of T1 (mean difference: −0.28 m/s, 95% CI: −0.35 to −0.22, *p* < 0.001). Animals also held their head more to the centre of the run throughout the gait cycle, with the position of the head to T1 in the left/right axis closer to 0 (centre of the run) compared with pre-stroke (mean difference: −2.72 cm, 95% CI: 5.42 to 0.02, *p* = 0.049). The vertical position of the head in relation to T1 was lower 3 days following stroke (mean difference −8.21 cm, 95% CI: −10.73 to −5.68, *p* < 0.001). T1 in relation to T13 was also lower (mean difference:−1.88 cm, 95% CI: −2.47 to −1.30, *p* < 0.001), as was T1 to L7 (mean difference: 2.27 cm, 95% CI: −2.88 to −1.67, *p* < 0.001), indicating lowering of the thorax following stroke.

#### 3.2.4. Baseline vs. post-stroke analysis of limb parameters

##### 3.2.4.1. Forelimb parameters

For the left forelimb (Supplementary Table S7), post-stroke duration in stance (mean difference: 0.13 s, 95% CI: 0.11 to 0.16, *p* < 0.001), swing (mean difference: 0.02 s, 95% CI: 0.01 to 0.03, *p* < 0.001), and stride (mean difference: 0.15 s, 95% CI: 0.12 to 0.18, *p* < 0.001) was longer than pre-stroke. An increase in the ratio of stance to stride (mean difference: 0.06%, 95% CI: 0.05 to 0.07, *p* < 0.001) and stance to swing (mean difference: 0.31%, 95% CI 0.24 to 0.39, *p* < 0.001) and decrease in swing to stride (mean difference: −0.06%, 95% CI: *p* = 0.001) was consequently observed. Lateral deviation of the hoof was also reduced (mean difference: −1.02 cm, 95% CI: −1.88 to −0.16, *p* = 0.02), as was forward (mean difference: −0.31, 95% CI: −0.38 to −0.23, *p* < 0.001), and vertical (mean difference: −0.04 s, 95% CI: 0.07 to 0.02, *p* = 0.003) swing velocity, range of the hoof height during swing (mean difference: −0.62 cm, 95% CI: −1.20 to – 0.03, *p* = 0.038), and stride length (mean difference: −7.41 cm, 95% CI: −9.25 to −5.58, *p* < 0.001). Joint angles of both the lower and upper forelimb were also influenced post-stroke. For the lower forelimb, this included a reduction in the range of the fetlock (mean difference: −3.9°, 95% CI: −6.62 to −1.18, *p* = 0.005) and carpus (mean difference: −2.81°, 95% CI: −5.58 to −0.05, *p* = 0.046) during swing. For the upper forelimb, an increase in the minimum (mean difference: 6°, 95% CI: 0.55 to 11.45, *p* = 0.031) and maximum (mean difference: 5.18°, 95% CI: −0.07 to 10.43, *p* = 0.053) angle of the elbow during stance and increase in the minimum angle of the elbow during swing (mean difference: 5.71°, 95% CI: 0.54 to 10.87, *p* = 0.03) was observed. When adjusted for velocity, the duration in stance (mean difference: 0.02 s, 95% CI: 0 to 0.05, *p* = 0.037) and stride (mean difference: 0.03 s, 95% CI: 0 to 0.5, *p* = 0.028) remained longer than pre-stroke, as did the increased ratio of stance to stride (mean difference: 0.02%, 95% CI: 0 to 0.3, *p* = 0.018), and decreased ratio of swing to stride (mean difference: −0.02%, 95% CI: −0.03 to 0, *p* = 0.018). Lateral deviation of the hoof remained reduced following adjustment (mean difference: −1.10 cm, 95% CI: −2.13 to −0.07, *p* = 0.036), as did decreased range of flexion-extension of the elbow during stance (mean difference: −3.03, 95% CI: −5.81 to −0.25, *p* = 0.033). When adjusted for velocity, no differences were observed for minimum, maximum or range of the fetlock or carpus joints in stance or swing (all *p* > 0.11), with the exception of the range of the elbow in stance as above.

For the right forelimb, stroke resulted in an increase in stance (mean difference: 0.14, 95% CI: 0.11 to 0.16, *p* < 0.001), swing (mean difference: 0.02, 95% CI: 0.01 to 0.02, *p* = 0.002) and stride (mean difference: 0.15, 95% CI: 0.12 to 0.18, *p* < 0.001) duration, and decrease in stride length (mean difference: −7.05 cm, 95% CI: −8.88 to −5.22, *p* < 0.001). This resulted in an increased ratio of stance to stride (mean difference: 0.06, 95% CI: 0.05 to 0.08, *p* < 0.001) and stance to swing (mean difference: 0.33%, 95% CI: 0.25 to 0.41, *p* < 0.001) and decrease in swing to stride (mean difference: −0.06%, 95% CI: −0.08 to −0.05, *p* < 0.001). A reduction in hoof lateral deviation (mean difference:-1.43 cm, 95% CI: −2.29 to −0.57, *p* = 0.001), hoof forward swing velocity (mean difference: −0.30 m/s, 95% CI: −0.37 to −0.22, *p* < 0.001), vertical swing velocity (mean difference: −0.05 m/s, 95% CI: −0.08 to 0.02, *p* < 0.001) and range on the hoof height during swing (mean difference: −0.78, 95% CI: −1.36 to −0.19, *p* = 0.009) was also observed. Regarding joint angles in the lower forelimb, the range of the fetlock was reduced during swing (mean difference: −3.31°, 95% CI: −6.03 to −0.60, *p* = 0.017) as was the range of the carpus during swing (mean difference: −3.55°, 95% CI: −6.31 to −0.79, *p* = 0.012). No differences in the upper right forelimb (elbow) were observed post-stroke (all *p* > 0.13). Following adjustment for velocity, differences in stance (mean difference: 0.03 s, 95% CI: 0 to 0.05, *p* = 0.02) and stride (mean difference: 0.03 s, 95% CI: 0 to 0.5, *p* = 0.039) duration, ratio of stance to stride (mean difference: 0.02%, 95% CI: 0.02 to 0.04, *p* = 0.004), swing to stride (mean difference: −0.02%, 95% CI: −0.04 to −0.01, *p* = 0.004) and stance to swing (mean difference: 0.08, 95% CI: 0.01 to 0.16, *p* = 0.033) remained, as did hoof lateral deviation (mean difference: −1.51 cm, 95% CI: −2.54 to −0.48, *p* = 0.004), vertical swing velocity (mean difference: −0.04 m/s, 95% CI: −0.08 to −0.01, *p* = 0.023), and hoof height range (mean difference: −0.72 cm, 95% CI: −1.43 to −0.01, *p* = 0.048). No differences were observed for joint angles when adjusted for velocity (all *p* > 0.22). The distance between the left and right forelimbs during stance was also reduced following stroke (mean difference: −3.84 cm, 95% CI: −5.92 to −1.77, *p* < 0.001, only valid as a measurement when unadjusted for velocity).

##### 3.2.4.2. Hindlimb parameters

For the left hindlimb, stroke resulted in an increase in stance (mean difference: 0.13 s, 95% CI: 0.10, to 0.15, *p* < 0.001), swing (mean difference: 0.03 s, 95% CI: 0.02 to 0.04, *p* < 0.001) and stride (mean difference: 0.16 s, 95% CI: 0.13 to 0.19, *p* < 0.001) duration. This influenced the ratio stance to stride (mean difference: 0.05%, 95% CI: 0.03 to 0.06, *p* < 0.001), swing to stride (mean difference: −0.05%, 95% CI: −0.06 to −0.03, *p* < 0.001) and stance to swing (mean difference: 0.25%, 95% CI: 0.17 to 0.32, *p* < 0.001). Stroke also resulted in a reduction in hoof forward swing velocity (mean difference: −0.43 m/s, 95% CI: −0.50 to −0.35, *p* < 0.001) and stride length (mean difference: −7.70 cm, 95% CI: −9.53 to −5.87, *p* < 0.001). Regarding left hindlimb joint angles of the lower limb, stroke increased the minimum (mean difference: 6.35°, 95% CI: 2.22 to 10.47, *p* = 0.003) and decreased the range of the fetlock in swing (mean difference: −8.34°, 95% CI: −11.06 to −5.63, *p* < 0.001). Stroke also decreased the maximum (mean difference: −5.07°, 95% CI: −7.77 to −2.37, *p* < 0.001) and range (mean difference: −4.72°, 95% CI: −6.81 to −2.63, *p* < 0.001) of the tarsus in stance, and decreased the maximum (mean difference: −6.53°, 95% CI: −9.67 to −3.39, *p* < 0.001) and range of the tarsus in swing (mean difference: −6.15°, 95% CI: −8.91 to −3.39, *p* < 0.001). For the upper hindlimb, stroke resulted in an increase in the maximum (mean difference: 5.81°, 95% CI: 0.56 to 11.06, *p* = 0.030) and range (mean difference: 2.80°, 95% CI: 0.41 to 5.19, *p* = 0.022) of the stifle in stance, an increase in the minimum stifle angle during swing (mean difference: 6.69°, 95% CI: 1.52 to 11.86, *p* = 0.011), and a decrease in the range during swing (mean difference: −3.85°, 95% CI: −6.14 to −1.56, *p* = 0.001). When adjusted for velocity, differences remained for stride duration, hoof forward swing velocity, minimum and range of the fetlock angle during swing, maximum and range of the tarsus angle in stance, maximum and range of the tarsus in swing, range of the stifle in swing, and range of the fetlock during stance became significant following adjustment (all *p* < 0.042) (Supplementary Table S8).

Right hindlimb parameters were also significantly impacted post-stroke. Specifically, stroke produced an increase in right hindlimb stance (mean difference: 0.12 s, 95% CI: 0.10, to 0.15, *p* < 0.001), swing (mean difference: 0.03 s, 95% CI: 0.02 to 0.04, *p* < 0.001), and stride (mean difference: 0.15 s, 95% CI: 0.12 to 0.18, *p* < 0.001) duration. This subsequently influenced the ratio of stance to stride (mean difference: 0.05%, 95% CI: 0.03 to 0.06, *p* < 0.001), swing to stride (mean difference: −0.05%, 95% CI: −0.06 to −0.03, *p* < 0.001), and stance to swing (mean difference: 0.25%, 95% CI: 0.17 to 0.32, *p* < 0.001). Stroke also produced in a reduction in hoof lateral deviation (mean difference: −1.81 cm, 95% CI: −2.67 to −0.95, *p* < 0.001), forward (mean difference: −0.41 m/s, 95% CI: −0.48 to −0.33, *p* < 0.001) and vertical (mean difference: −0.05 m/s, 95% CI: −0.08 to −0.02, *p* = 0.001) swing velocity, range of the hoof height in swing (mean difference: −0.64 cm, 95% CI: −1.23 to −0.06, *p* = 0.032) and stride length (mean difference: −7.44 cm, 95% CI: −9.28 to −5.61, *p* < 0.001). Regarding right hindlimb joint angles, stroke influenced the lower limb, resulting in an increase in the minimum (mean difference: 6.95°, 95% CI: 2.82 to 11.07, *p* = 0.001) and decrease in the range (mean difference: −8.19°, 95% CI: −10.90 to −5.47, *p* < 0.001) of the fetlock during swing. Stroke also decreased the maximum (mean difference: −5.33°, 95% CI: −8.02 to −2.63, *p* < 0.001) and range (mean difference:−4.91°, 95% CI: −7.00 to −2.81, *p* < 0.001) of the tarsus in stance and decreased the maximum (mean difference: −7.51°, 95% CI: −10.65 to −4.37, *p* < 0.001) and range (mean difference: −6.68°, 95% CI: −9.44 to −3.91, *p* < 0.001) of the tarsus during swing. For the upper hindlimb, stroke also produced an increase in the maximum (mean difference: 7.77°, 95% CI: 2.52 to 13.02, *p* = 0.004) and range (mean difference: 3.69°, 95% CI: 1.30 to 6.08, *p* = 0.002) of the stifle during stance, increase in the minimum angle of the stifle during swing (mean difference: 7.41°, 95% CI: 2.24 to 12.58, *p* = 0.005), and a decrease in the range of the stifle in swing (mean difference: −2.35°, 95% CI: −4.64 to −0.06, *p* = 0.044). Following velocity adjustment, differences remained for stride duration, hoof lateral deviation, forward and vertical swing velocity, minimum and range of the fetlock during swing, maximum and range of the tarsal angle in swing (all *p* < 0.038) (Supplementary Table S8).

##### 3.2.4.3. Measures of asymmetry

Time-by-limb interactions to determine if the effect of time (pre- vs. post- stroke) differed by limb are reported in Supplementary Table S9, both adjusted and un-adjusted for velocity. Parameters with a significant interaction between time and limb side included hoof forward swing velocity (*p* = 0.027), range of the fetlock angle during swing (*p* = 0.01), range of the carpus/tarsus in stance (*p* = 0.012) and maximum angle of the carpus/tarsus in swing (*p* = 0.002). The interaction remained significant (all *p* < 0.05) for these variables following adjustment for velocity. Further, the interaction term for the range of the elbow during stance was significant following velocity adjustment (*p* = 0.049).

To probe these observations further, differences between ipsi- and contra- lateral limb pairs (fore- and hind- limbs), indicative of asymmetry, are reported in Supplementary Table S10 (forelimbs) and Supplementary Table S11 (hindlimbs). No differences were observed between left and right forelimbs post-stroke when unadjusted and adjusted for velocity (all *p* > 0.17, Supplementary Table S10). Significant differences were; however, observed between the left and right hindlimbs [highlighted by an asterix (^*^) in Supplementary Table S11]. This included greater swing velocity in the left hindlimb (mean difference: −0.04 m/s, 95% CI: −0.07 to −0.01, *p* = 0.012), a greater range of the hoof height in swing of the left hindlimb (mean difference: −0.74 cm, 95% CI: −1.32 to −0.15, *p* = 0.014), and a decreased range of the left hindlimb fetlock in stance (mean difference: 2.68°, 95% CI: 0.90 to 4.46, *p* = 0.003). Left hindlimb angles were also significantly greater than the right post-stroke, including the minimum (mean difference: −3.30°, 95% CI: −6.27 to −0.33, *p* = 0.030) and maximum (mean difference: −2.94°, 95% CI: −5.64 to −0.25, *p* = 0.032) angle of the tarsus in stance, and minimum (mean difference: 3.58°, 95% CI:−6.71 to −0.45, *p* = 0.025) and maximum (mean difference:−4.12°, 95% CI:−7.25 to −1.00, *p* = 0.010) tarsal angle in swing. Following velocity adjustment, significant differences for each variable remained (all *p* < 0.025).

### 3.3. Gender differences

Male and female animals had comparable function at baseline for total neurological score and each global outcome measure of interest (all *p* > 0.05, Supplementary Table S12). For baseline forelimb-specific kinematic measures (Supplementary Table S13), genders were also comparable for the majority of outcomes assessed with the exception of stance (mean difference: −0.03, 95% CI: −0.05 to −0.01, *p* = 0.007), swing (mean difference: −0.02, 95% CI:- 0.03 to −0.00, *p* = 0.009) and stride (mean difference: −0.05, 95% CI: −0.07 to −0.02, *p* = 0.002) duration in the left forelimb, and stance (mean difference: −0.03, 95% CI: −0.05 to −0.00, *p* = 0.021), swing (mean difference: −0.01, 95% CI: −0.03 to −0.00, *p* = 0.038) and stride (mean difference: −0.04, 95% CI: −0.07 to −0.01, *p* = 0.007) duration in the right forelimb, all of which were longer for male animals. In the hindlimbs (Supplementary Table S14) male animals also displayed a longer duration in swing for both left (mean difference: −0.03, 95% CI: −0.05 to −0.02, *p* < 0.001) and right (mean difference: −0.03, 95% CI: −0.05 to −0.02, *p* < 0.001) hindlimbs, in addition to a longer duration in stride for both left (mean difference: −0.04, 95% CI: −0.07 to −0.01, *p* = 0.006) and right (mean difference −0.04, 95% CI −0.07 to −0.01, *p* = 0.008) hindlimbs. Males also exhibited a decreased range of the fetlock in swing for both left (mean difference 3.62, 95% CI: 0.95 to 6.29, *p* = 0.008) and right (mean difference 4.19, 95% CI: 1.52 to 6.86, *p* = 0.002) hindlimbs.

Following stroke there were no differences observed between genders for total neurological score or global kinematic measures (all *p* > 0.05, Supplementary Table S15). For the forelimbs (Supplementary Table S16), males displayed an increase in stance duration in the left forelimb (mean difference: −0.03, 95% CI: −0.06 to −0.01 *p* = 0.019), an increase in swing duration in both the left (mean difference: −0.02, 95% CI: −0.04 to −0.01 *p* = 0.002) and right (mean difference: −0.03, 95% CI: −0.05 to −0.01, *p* = 0.004), and an increase in stride duration in both the left (mean difference: −0.06, 95% CI: −0.09 to −0.02, *p* = 0.001) and right (mean difference: −0.05, 95% CI: −0.09 to −0.02, *p* = 0.002) limbs post-stroke. In the hindlimbs (Supplementary Table S17), males also exhibited an increased duration in swing in the left (mean difference: −0.03, 95% CI: −0.05 to −0.01, *p* < 0.001) and right (mean difference: −0.04, 95% CI: −0.05 to −0.02, *p* < 0.001) hindlimbs, and increased duration in stride in the left (mean difference: −0.05, 95% CI: −0.08 to −0.1, *p* = 0.005) and right (mean difference: −0.05, 95% CI: −0.08 to −0.02, *p* = 0.003) hindlimbs. Males also had a decreased range of the fetlock angle in stance, which was isolated to the right hindlimb (mean difference: 4.32, 95% CI: 1.36 to 7.28, *p* = 0.004).

### 3.4. Infarct volume

All animals displayed evidence of infarction in the right parietal lobe encompassing the thalamus and/or cortical regions as quantified on DWI at 3 days post-stroke. Median (IQR) infarct volume was 2.7 (1.4 to 11.9) cm^3^ (raw values shown in Table 2). Those animals with larger infarcts exhibited a greater degree of midline shift, indicative of space occupying oedema (Supplementary Table S18), although infarct volume was not corrected for oedema due to lesion variability. Due to significant variation in lesion volume, animals with infarcts >18 cm^3^ (median: 21.99 cm^3^, IQR: 19.33 to 25.46 cm^3^, *n* = 5) were compared to those with infarcts measuring <6 cm^3^ (median: 1.99 cm^3^, IQR: 0.90 to 3.19 cm^3^, *n* = 15) (Supplementary Table S19) for each of the following variables: total neurological score, kinematic global measures including; mean absolute velocity and mean head to T1, and limb specific measures for the fore- and hind- limbs (both left and right) including minimum, maximum and range of the fetlock instance, minimum, maximum and range of the fetlock in swing, and duration of stance, swing, and stride. All limb-specific measure were adjusted for velocity. Differences were assessed as per the gender analysis (Section 2.8.3).

**Table 2:** Variables included for principal component analysis.

**Animal ID**	**Infarct volume (cm^3^)**	**Total neurological score (0–36)**	**Mean velocity (m/s)**	**Mean head to T1 (cm)**	**Swing duration right (s)**	**Swing duration left (s)**	**Stance duration left (s)**	**Minimum left fetlock angle in swing (*°*)**
1	18.49	7.5	0.90	2.31	0.40	0.41	0.60	−18.31
2	0.35	16.25	0.67	−3.42	0.42	0.54	0.62	−44.21
3	1.99	7.5	0.88	−0.15	0.36	0.37	0.60	−18.14
4	3.66	12.5	0.92	−3.63	0.38	0.35	0.55	−9.38
5	4.07	11.5	0.96	−2.93	0.39	0.36	0.59	−28.61
6	3.19	15.5	0.91	−4.69	0.38	0.38	0.64	−25.78
7	0.90	2.5	1.28	11.22	0.37	0.38	0.41	−28.02
8	0.63	5	1.17	5.14	0.38	0.36	0.44	−37.84
9	5.28	0.5	1.19	3.66	0.35	0.36	0.43	−34.27
10	0.84	10	1.29	15.39	0.35	0.35	0.36	−8.91
11	2.68	4.5	1.18	8.33	0.36	0.34	0.39	−15.22
12	1.53	5	1.24	4.53	0.35	0.34	0.41	−50.90
13	21.99	9.5	0.68	−5.33	0.36	0.40	0.72	−33.65
14	25.46	12	0.97	1.01	0.42	0.42	0.59	−16.65
15	2.66	6.25	0.88	−0.67	0.39	0.39	0.63	−30.20
16	2.01	4.25	0.98	7.49	0.38	0.37	0.50	−26.39
17	1.26	2.25	0.95	11.40	0.39	0.40	0.53	−13.97
18	19.33	7.5	0.96	6.23	0.35	0.35	0.56	−20.67
19	29.95	12	1.12	−19.41	0.40	0.36	0.60	−6.02
20	1.71	8.25	1.20	5.72	0.34	0.31	0.42	−12.60

No differences were observed between infarcts >18 cm^3^ and <6 cm^3^ for total neurological score or either kinematic global measure (all *p* > 0.05, Supplementary Table S19). For forelimb specific parameters (Supplementary Table S20), animals with infarcts >18 cm^3^ were revealed to have increased forelimb stance duration compared with animals with infarcts <6 cm^3^, although this was observed in both the left (mean difference: 0.04, 95% CI: 0.01 to 0.06, *p* = 0.018) and right (mean difference: 0.05, 95% CI: 0.01 to 0.08, *p* = 0.006) limbs. In the hindlimbs (Supplementary Table S21), stance duration was also significantly longer in animals with infarcts >18 cm^3^, although this was also observed in both left (mean difference: 0.06, 95% CI: 0.02 to 0.09, *p* = 0.001) and right (mean difference: 0.05, 95% CI: 0.02 to 0.09, *p* = 0.002) hindlimbs. Marked differences were also observed in the maximum angle of the fetlock in swing in both the left (mean difference: −8.82, 95% CI: −16.04 to−1.60, *p* = 0.017) and right (mean difference:−7.27, 95% CI: −14.49 to −0.05, *p* = 0.048) hindlimbs, in addition to the range of the fetlock in swing for both left (mean difference: −6.79, 95% CI: −12.44 to −1.14, *p* = 0.019) and right (mean difference: −7.18, 95% CI: −12.83 to −1.53, *p* = 0.013) hindlimbs.

### 3.5. Principal component analysis

Due to high correlations (*r* > 0.85), the following variables were removed from the PCA: minimum and maximum fetlock angle in stance, maximum fetlock angle in swing, and stance and stride duration. Gait kinematic variables below the threshold for KMO (<0.5) were excluded from PCA, including: minimum, maximum, and range of the right fetlock in stance, maximum and range of the left fetlock in stance, the minimum and maximum angle of fetlock in swing (left right forelimbs) and stance duration. The final PCA was thus fitted with infarct volume, total neurological score, kinematic global variables including mean absolute velocity and mean position of the head to T1, and limb specific variables for the left forelimb including minimum angle of the fetlock in swing and stance duration, and duration in swing for both left and right forelimbs. A summary of these variables is provided in Table 2. The final PCA produced an overall KMO = 0.67, implying that the data was appropriate for performing PCA. Two components had eigenvalues >1 which explained 67.2% of the variance. Bartlett's test of sphericity showed that there was an interrelationship among the final variables reported (χ^2^ = 91.7, *p* < 0.001).

**Figure 4:**
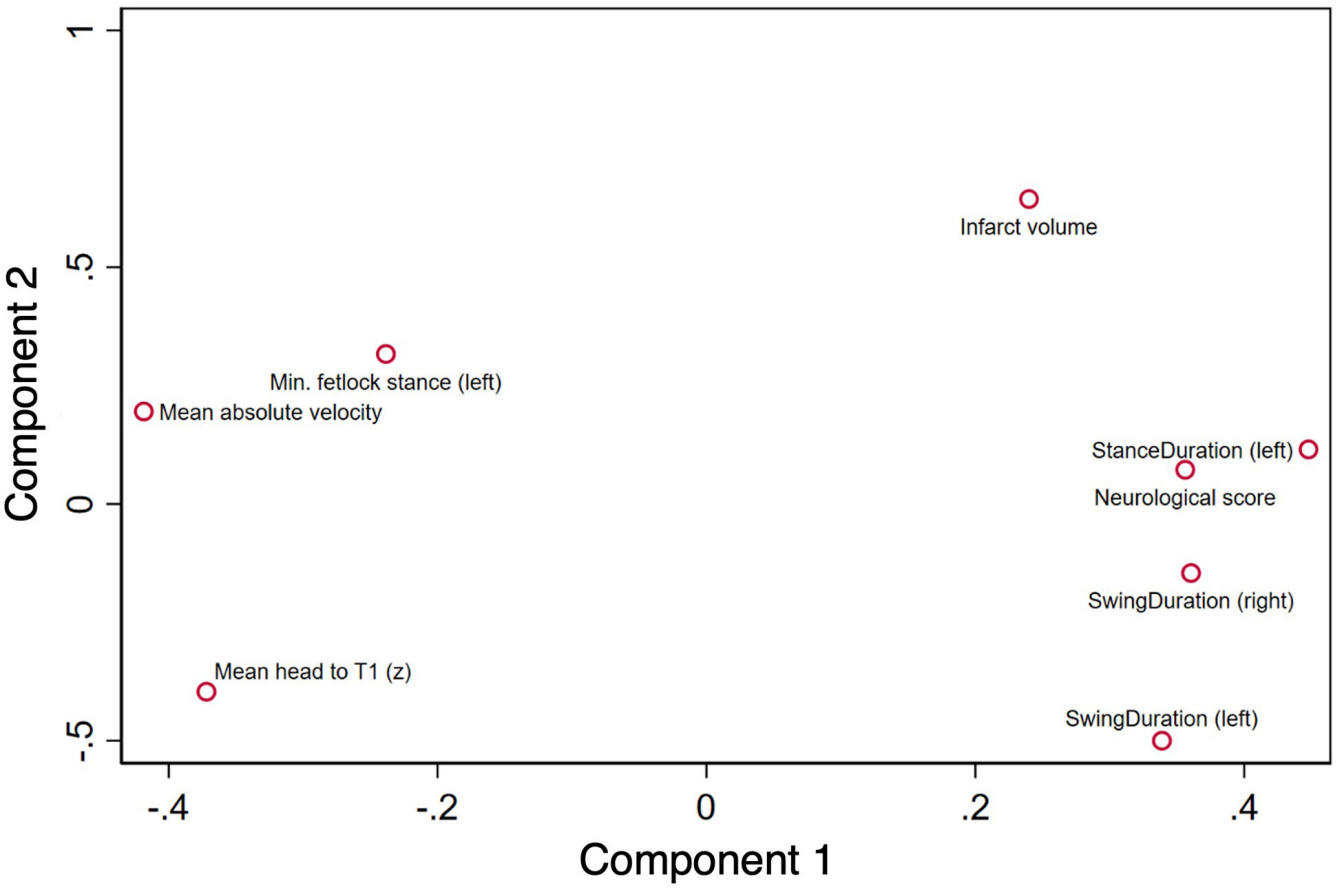
Principal component analysis loading plot for variables. Principal component 1 shows positive associations with stance duration (left forelimb), total neurological score, swing duration (left and right forelimbs); and negative associations with mean head to T1 and mean absolute velocity. Principal component 2 relates positively to infarct volume and minimum fetlock stance (left forelimb); and negatively to swing duration (left forelimb) and mean head to T1.

The final PCA yielded two components (Table 3), with the summary loading plot shown in Figure 4. Principal component 1 (PC1) accounted for 51.5% of the overall variance. PC1 was characterised by positive associations with stance duration of the left forelimb, total neurological score, swing duration (both left and right forelimbs); and negative associations with mean head to T1 and mean absolute velocity. Principal component 2 (PC2) related positively to infarct volume and minimum fetlock in stance (left forelimb); and negatively to swing duration (left forelimb) and mean head to T1.

**Table 3:** Component loadings and KMO values for all variables included in the PCA.

	**Component loadings**		
**Component (% variance)**	**Principal component 1**	**Principal component 2**	**Kaiser-Meyer Olkin**
**Component 1 (51.5%)**			
Stance duration (left forelimb)	0.45	0.12	0.63
Total neurological score	0.36	0.07	0.92
Swing duration (right forelimb)	0.36	−0.15	0.72
Swing duration (left forelimb)	0.34	−0.50	0.65
Mean head to T1	−0.37	−0.40	0.68
Mean absolute velocity	−0.42	0.20	0.68
**Component 2 (15.7%)**			
Infarct volume	0.24	0.64	0.68
Minimum fetlock stance (left forelimb)	−0.24	0.32	0.63
Overall			66.9

## 4. Discussion

In this study we present a comprehensive approach to assessing functional outcome in an ovine model of ischaemic stroke. First, through adaptation of a neurological assessment score, we characterised the pre- and post-stroke response of animals, including demeanour, behaviour, and postural reactions. Second, using motion capture, we developed a method to detect changes in gait kinematics, representing the first description of this approach to functional assessment in an ovine stroke model. We have shown both approaches to be repeatable in healthy animals through comparison of baseline pre-stroke trials, and subsequently used these findings to assess changes in functional outcomes at 3 days post- stroke.

### 4.1. Neuroscore

Neurological composite scoring remains a valuable tool both in pre-clinical models and clinical patients to assess functional outcomes across the post-stroke time course. Through adaptation of an ovine neurological score, this study demonstrated that composite scoring was a repeatable means to assess neurological function for most measures of interest. Stroke prognostic scores such as the NIHSS perform well in predicting clinical outcomes post-stroke (49). Post-stroke neuroscore values in the present study reflected significant functional impairment post-ictus, in keeping with the clinical literature and previous ovine studies (27). The most profound deficits observed included alterations in demeanour, including lowering of the head, and general apathy. It must be highlighted that the post-operative course is frequently reported as a potential confounder of animal demeanour (50), such that this may not be an observation linked solely to post-stroke sequalae given assessment was carried out 3 days post-operatively.

In comparison, postural disturbances are a reliable indicator of veterinary neurological dysfunction, including stroke (51). Herein, post-stroke animals displayed abnormal movement of the forelimbs, evidenced by both ipsi- and contra- lateral postural reactions during conscious proprioceptive positioning. Slight variability was observed in baseline postural reaction tests and postural assessment of the left limbs was not considered repeatable. This likely reflects difficulty in performing postural reactions in large animal species due to the need for significant manual handling, in addition to the fact that on occasion, animals were unwilling to perform the task, lying down or showing no desire to respond to the perturbation. In evaluating ipsi- vs. contra-lateral deficit post-stroke, postural reaction tests revealed significant deficits in the left limbs when compared to pre-stroke, although differences were also observed in the right limbs following stroke onset. These findings suggest global deficits, rather than limb specific changes, were apparent in our ovine cohort 3 days following stroke.

### 4.2. Gait kinematics

#### 4.2.1. Repeatability of gait kinematics

Assessment of gait kinematics using motion capture sought to detect subtle changes beyond the scope of composite scoring. Repeatability of human gait kinematics using motion capture in multiple laboratories is good (ICC > 0.80), supporting its use as a valuable tool across a range of environments and in different species (52). When determining the repeatability of global outcome measures of interest, this study revealed that the position of the head in relation to T1, T13, and L7 across baseline trials had good repeatability, with animals consistently walking with their head upright, and slightly towards the right of the functional run. Although the average speed of walking was comparable across animals, velocity had poor repeatability. This may have subsequently influenced limb specific parameters of interest given gait patterns change as a function of velocity (53, 54). This is true under normal physiological conditions, and factoring in all velocity-related changes when assessing gait in disease is especially challenging. Previous studies have suggested that observation of gait characteristics when speed is not controlled leads to variation from trial to trial, which is true for both experimental animals (55–58) and human participants (59–61). Neglect of velocity has also been proposed to lead to oversimplification of analysis and loss of potentially valuable data (54).

To address this, previous studies have used treadmills for functional assessment to control for velocity (35–37). Although this offers the advantage of regulating walking speed, the selected speed of the treadmill has been shown to directly influence walking patterns (62–64). Importantly, faster speed has been shown to facilitate a more normal walking pattern following stroke in humans (65). Given the unilateral effects of MCAo, the ability to accurately assess deficits of symmetry is imperative. Allowing animals to walk at self-selected pace may enable more accurate assessment of asymmetric gait following stroke, which can retrospectively be adjusted for velocity. In the current study, significant differences between left and right hindlimbs were observed following stroke. These differences remained when adjusted for walking speed *via* regression analysis. Use of regression-based analyses has been suggested as a robust approach to translational gait analysis and may be particularly relevant in the setting of stroke (54, 57). Indeed, the application of regression-based velocity adjustment reported in the current study suggests the method is reproducible. This enables application in various experimental conditions and environments such as those where velocity is not a controlled measure. Enabling animals to walk at their own pace also allowed us to determine the ‘comfortable' walking speed pre- and post-injury: an important consideration from an animal welfare perspective. Thus, adjusting for velocity during post-processing is of benefit from both ethical and experimental perspectives, and favours the generation of more reliable data.

Regarding limb-specific outcome measures, we observed good repeatability for most, but not all, variables, with the few that were not repeatable varying between left and right limbs. Specifically, all joint angles were repeatable except for the left forelimb and right hindlimb minimum fetlock angle during swing, regardless of velocity adjustment. Retro-reflective markers on the distal limbs were smaller (9 mm) than the other markers (15 mm), which was essential due to the close proximity of placement on the hooves. Consequent reduction in spatial resolution necessitated more extensive gap filling of these markers during post-processing. Tattooing was also less distinguishable over the superficial bones of the distal limb, such that reattachment of markers at these sites may have been more variable. Together, these factors may have introduced more error, potentially confounding experimental results. Other fetlock angle parameters; however, were repeatable in baseline testing, including the minimum fetlock angle during swing for the right forelimb. Discrepancy may thus also represent variation of animal behaviour during overland walking. Future analyses should aim to improve data capture for the distal forelimbs and focus on assessing repeatable measures reported herein to accurately evaluate the effect of stroke and post-stroke interventions on gait kinematics.

The baseline gait outcomes reported in this study were generally consistent with other gait assessments performed in healthy sheep. Shelton and colleagues reported a stride length of approximately 1 m in mature female sheep (66), comparable to the present study (~98 cm for all limbs). The duration of the gait cycle phases was also consistent with previous studies (35, 37, 67–70) as summarised in Table 4. Limb joint flexion and extension throughout walking has also been reported in the ovine gait literature. Previous studies have primarily focused on the upper hind limb [stifle and tarsal (hock) joints]. Tapper et al. (68) observed a minimum stifle flexion of −77° and maximum of −43°, with a range of 34 ° in healthy female Suffolk sheep (68) over the entire gait cycle. In the present study, for the left hindlimb we observed a minimum stifle angle of −69° to a maximum −45° in stance (range of 23°), and a minimum stifle angle of −81° to a maximum of −35° in swing (range of 45 °), calculated as mean values across all animals. For the right hindlimb, a minimum stifle angle of −71° to a maximum of −48° was observed during stance, and a minimum of −83° to a maximum of −38° in swing (a range of 23° in stance and 45 ° in swing, comparable to the left hindlimb). There is limited published data regarding the fetlock angle of ruminants; however, the fetlock angle of horses during gait has been reported (71, 72), with the overall pattern of the gait cycle of the equine fetlock qualitatively comparable to the sheep in the current study at baseline (71–73).

**Table 4:** Average stride, swing and stance duration in clinically healthy sheep.

	**Forelimb (L/R where applicable)**			**Hindlimb (L/R where applicable)**		

**Study**	**Stance (s)**	**Swing (s)**	**Stride (s)**	**Stance (s)**	**Swing (s)**	**Stride (s)**
**Present study**	0.41	0.36	0.77	0.43	0.34	0.77
Tapper et al. (68)	–	–	–	–	–	–/0.98
Tapper et al. (69)	–	–	–	–	–	–/0.94
Agostinho et al. (67)	0.41	0.28	0.70	0.43	0.27	0.69
Safayi et al. (35)	0.46	0.37	0.84	0.47	0.37	0.84
Wilson et al. (37)	0.51/0.49	0.33/0.34	0.84	0.49	0.35	0.84
Kim and Breur (70)	0.55	0.27	0.82	0.57	0.25	0.82

#### 4.2.2. Post-stroke assessment of gait kinematics

Following stroke, sheep had reduced forward velocity and a lowered head position relative to T1, in addition to lowering of the shoulders and thorax (position of T1 to T13 and T1 to L7 respectively). These findings potentially indicate motor deficit and/or animal apathy. Post-stroke apathy, mood and emotional disturbances are commonly reported clinically, presenting as a loss of motivation and initiative (74). Conducting cognitive tasks may provide a more accurate measure of motivation, and systems developed for use in sheep for other pathologies (75–78) may be a helpful avenue for assessment in ovine stroke models to probe underlying mechanisms.

**Figure 5:**
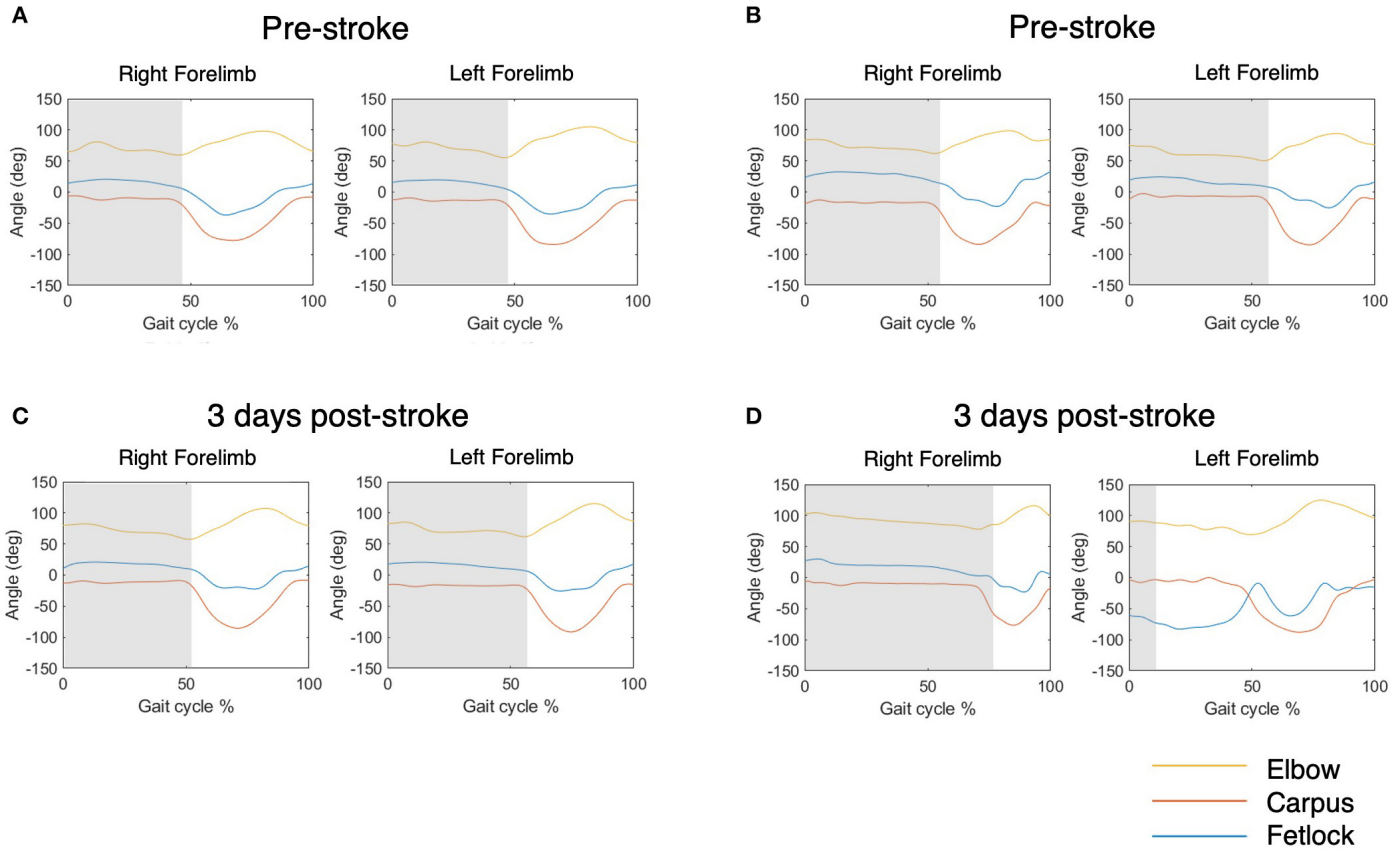
Exemplar elbow, carpus and fetlock joint angle data over 0–100% of the gait cycle for two animals. Shaded grey indicates the stance phase. **(A, B)** Show joint angles pre stroke and **(C, D)** show joint angles 3 days following stroke. **(A, C)** This animal did not display significant change of the fetlock joint angle pattern following stroke, as identified by the blue line in the left forefoot. **(B, D)** In comparison, this animal displayed significant fetlock dysfunction, with a decreased left fetlock angle throughout the cycle and a reduction of left stance duration.

Regarding limb-specific parameters, swing, stance, and stride durations were substantially longer post-stroke compared to baseline. These changes were observed in both the ipsi- and contra- lateral fore- and hind-limbs. This, in conjunction with decreased velocity, indicates that animals were less willing/able to execute forward movement. Furthermore, lateral deviation of the hoof in both left and right forelimbs was less than pre-stroke, as were forward and lateral swing velocity, indicating more “drag” of the limb and slower pace, respectively. Dysfunction of the left forelimb, contralateral to the lesion, was qualitatively observed following stroke, as per previous studies (27, 30). However, this was not uniform in all animals, as shown in the exemplar data for two animals in Figure 5. Therefore, over all animals, we did not detect pre-/post-stroke differences in joint angle minimum, maximum and range of the left forelimb, with the exception of the range of the elbow joint. It is important to note that the minimum angle of the elbow had poor repeatability across baseline sessions, so the significance of this finding is questionable.

Adjusting for velocity reduced the mean difference between pre- and post-stroke for most outcomes, although significant differences remained for stance, stride, and hoof lateral deviation, irrespective of limb. This suggests that the change observed in some of the gait parameters following stroke may reflect an alteration in gait signature due to the underlying pathology, not just a change in gait speed. Although these changes remained, they were not as anticipated when observing the animal qualitatively. As per the exemplar data (Figure 5), if significant side-dependent deficit was present, a reduction in stance of the affected limb due to hemiparesis and inability to execute motor control, and subsequent compensatory increase in stride of the unaffected limb, would be expected. The lack of significant differences between pre- and post-stroke forelimb joint angles was unexpected, particularly of the fetlock. Although fetlock paresis was observed during testing, deficit was not pronounced for every animal, and if deficit was present, it was not consistent for every step of the gait cycle. Consequently, although we observed a qualitative loss of motor control of the left (contralateral) fetlock in 5/20 animals, this deficit was not captured in the data reported herein.

Furthermore, although we did not observe asymmetry between the left and right forelimbs post-stroke, differences in symmetry were observed in the hindlimbs. Results suggest the left hindlimb, contralateral to the stroke, was impacted more than the right, particularly the tarsus joint. Specifically, the minimum and maximum angle of the tarsus was greater in the left compared with the right hindlimb, which was evident during both stance and swing. This may be indicative of left hindlimb deficit, especially as both the minimum and maximum angle of the tarsus had moderate-to-good repeatability across baseline trials, both adjusted and un-adjusted for velocity. Nevertheless, we cannot discount this finding may be indicative of variability between animals following stroke, rather than discreet post-stroke deficits of symmetry. Further trials and/or sessions post-stroke may be necessary to increase the likelihood of accurately detecting sided deficit, suggesting an avenue for future development.

Following stroke, male animals exhibited extended stance, swing and stride duration of the forelimbs, and longer swing and stance duration in the hindlimbs compared with female animals. Nevertheless, these findings were observed at baseline, and appear to reflect inter gender variation rather than a consequence of male animals being more affected by the stroke itself.

### 4.3. Relationship between gait kinematics, neuroscore, and MRI parameters

The PCA revealed that the parameters included clustered into two components of associated variables. For component 1, stance duration (left and right), swing duration (right) and neurological score had a positive association, while mean absolute velocity and mean head to T1 were negatively associated. Given the relationship between stance and swing duration in the normal gait cycle it is logical that these variables load to the same component. Further, the inclusion of neurological score with this cluster of variables is also logical considering that the neurological assessment provides an indicator of overall disability and encompasses measures of balance which likely align with the stance and swing variables. The negative association between mean absolute velocity and mean head to T1 likely reflects the observation that following stroke, animals that were more disabled tended to walk more slowly and had an apathetic demeanour, including a lowered head position whilst walking through the run. Decreased velocity subsequently increased the duration of stance and swing, hence the negative association. Minimum fetlock during stance (left) and infarct volume were both positively associated with component 2. The component loading plot showed that infarct volume did not load strongly onto component 1, and this likely reflects the variation in infarct volume we observed within the cohort following stroke.

Taken together, these findings suggest that there are some associations between neuroscore, gait kinematic and infarct volume variables, but do not support one measure being used in isolation. The results highlight the importance of a multimodal approach to assessing post-stroke outcome that encompasses both medical imaging information along with assessment of function (neuroscore, gait kinematics). In pre-clinical studies, the use of multiple outcome measures encompassing infarct volume and behavioural assessment serve to underscore the recommendations of the Stroke Therapeutic and Industry Roundtable (STAIR) preclinical guidelines (79). As different neurological deficits recover at varying rates post stroke (80), modality specific approaches to assess functional outcomes of interest may be warranted when assessing putative stroke therapies and should be factored into experimental design.

Finally, despite significant variation in infarct volume, animals with comparably large stroke volume (>18 cm^3^) compared with animals with smaller stroke volumes (<6 cm^3^) only exhibited a worsening of functional deficit in limb specific variables. Specifically, animals with a greater lesion burden displayed an increase in stance duration of the forelimbs, although this was not isolated to the contralateral limb. Furthermore, although a significant increase in stance duration was also observed in the hindlimbs, this was not unilateral. These findings suggest an overall increase in global deficit for animals with a greater stroke burden, rather than the unilateral impairment often seen clinically.

### 4.4. Limitations and future directions

There were several limitations in this study. Regarding composite scoring, we did not assess for sensory deficits despite inclusion in clinical stroke scoring systems. Previous studies have reported sheep rapidly habituate to nociceptive stimulation (27), and thus we chose to focus on behavioural and motor deficits. Regarding kinematics, we must firstly acknowledge that reflective markers attached to areas with more overlying tissue were prone to skin motion artefact. While marker pins inserted directly into the bone can eliminate skin motion artefact, this was not possible from an animal welfare perspective given pins can be painful and increase likelihood of infection, especially relevant given the large number of markers in the present study. Skin motion artefact was minimised by selecting marker positions with minimal overlying soft tissue, not performing analysis of joints/bones with substantial overlying muscle mass, and tattooing the skin to make marker placement repeatable. Secondly, we only performed 2D analysis predominantly in the sagittal plane. Three-dimensional joint angle analysis provides more comprehensive (i.e., rotations about three axes) and accurate (i.e., relative to anatomical coordinate systems rather than a GCS) joint angle assessment. We were limited in our approach due to the relative size of the animal, where given the number of joints assessed, there was insufficient space on rigid bodies to place additional markers. Future analyses should focus on refining the assessment to outcomes of the most relevance and where possible, ensuring markers are placed on rigid bodies. Thirdly, due to 2D analysis, any deviation from straight line walking in the forward direction (y axis in the GCS) could lead to errors in sagittal plane measures. We sought to minimise this by limiting the width of the run (1 m), using only one gait cycle per trial for which the animal was walking over the centre of the capture volume, and discarding trials where animals deviated from straight line walking. Fourthly, although this represents a comprehensive study for a large animal model, the number of animals used may limit interpretation of the statistical analyses. Certainly, utilising an even larger sample size than employed in the current study would limit variability and improve PCA interpretation.

It must also be acknowledged that for gait kinematics, neuroscore, and MRI, we only report a single time-point post-stroke. To accurately capture the temporal profile of post-stroke changes, functional studies should, ideally, mimic the clinical scenario where assessment is performed up to 90 days following stroke onset. Nevertheless, the purpose of the current study was to describe the capability of the functional assessment methods, in particular gait kinematics, to detect changes at 3 days post-stroke, rather than to characterise the temporal profile of post-stroke functional changes in detail. It must be acknowledged that stroke may not be fully organised by this time and animals may still be affected by the post-operative course; however, the decision to focus on day 3 post-stroke was made to avoid any residual effect of long-duration anaesthesia at day 1, and prior to onset of space-occupying oedema at day 5 post-stroke (30). A follow up study which goes beyond day 3 to provide a comprehensive and long-term assessment of post-stroke functional changes in this model is certainly warranted.

Finally, the 2 hour transient MCAo model reported herein was associated with a greater variability in lesion volume compared with permanent MCAo stroke (7.40 ± 9.59 cm^3^ at 3 days compared with 16.3 ± 5.2 cm^3^ at 1 day) (27), which may reflect differing arterial collateralisation between individual animals. In addition to large vessel stroke such as the MCA infarction described here, it is also pertinent to investigate the functional consequences of small vessel stroke. Specifically, lacunar infarcts typically have quite favourable functional prognoses (81), although there is a paucity of small vessel stroke models described in the literature, representing an avenue for future research.

## 5. Conclusions

Functional outcome is a major end-point in stroke clinical trials, and an essential component of pre-clinical stroke models. In this study we developed and described comprehensive methods to assess function post-stroke in a clinically-relevant ovine model. Following stroke, animals exhibited deficit, observed both *via* composite scoring and kinematically *via* motion capture. Taken together, these methods of functional assessment may provide an opportunity for the evaluation of medical and surgical interventions following stroke, and assessment of their contribution to function in a sheep model.

## Data availability statement

The original contributions presented in the study are included in the article/Supplementary material, further inquiries can be directed to the corresponding author.

## Ethics statement

The animal study was reviewed and approved by the South Australian Health and Medical Research Institute Animal Ethics Committee.

## Author contributions

AS-A, RT, and CJ wrote the manuscript. AS-A, OM, RT, AL, and CJ assisted in experimental design. AS-A designed the final experiments and performed the surgery, post-processing, and analysis of the data. OM, IB, and LE performed the experiments and assisted in all animal handling and welfare. JC produced the MATLAB code for analysis of gait kinematics. RC assisted in design of the functional run and determination of anatomical landmarks for assessments. GW and JS assisted in MRI analysis. KH performed statistical analyses. AS-A, OM, IB, LE, JC, KH, RC, AL, GW, JS, RT, and CJ read, edited, and revised the manuscript. All authors contributed to the article and approved the submitted version.
