# Supplementary material for: Neurological scoring and gait kinematics to assess functional outcome in an ovine model of ischaemic stroke
Source: Front Neurol. 2023 Feb 20;14:1071794. doi: 10.3389/fneur.2023.1071794 (PMC9986303; doi:10.3389/fneur.2023.1071794)
Supplement: Supplementary file 1 [file Data_Sheet_1.docx]

**Supplementary Figures and Tables**


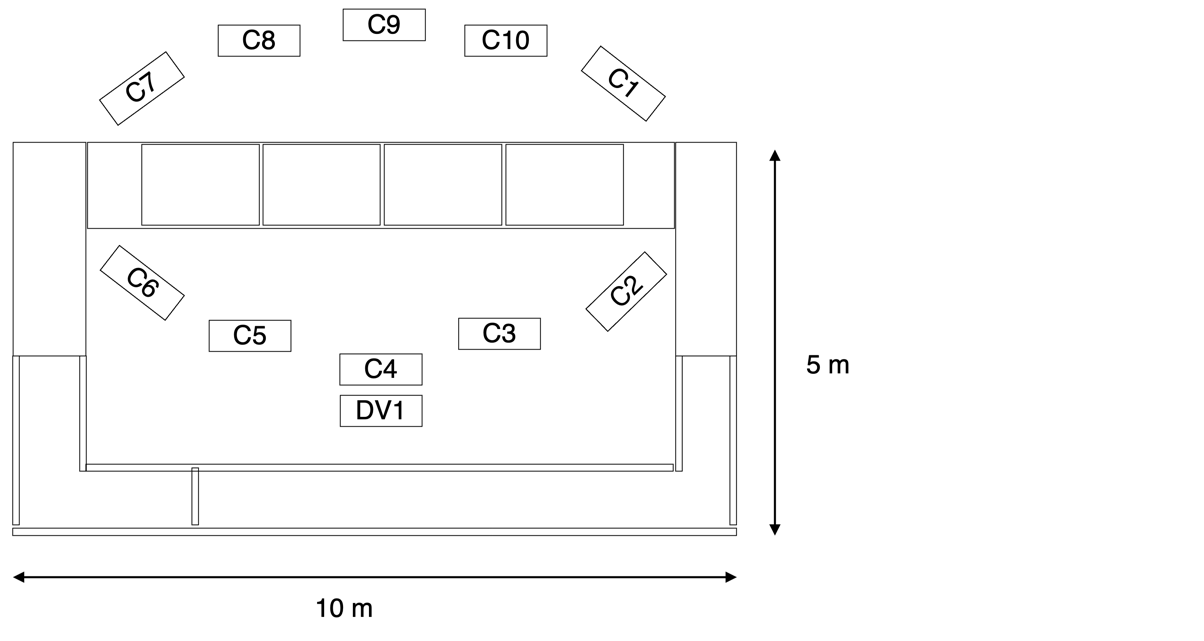


A

B


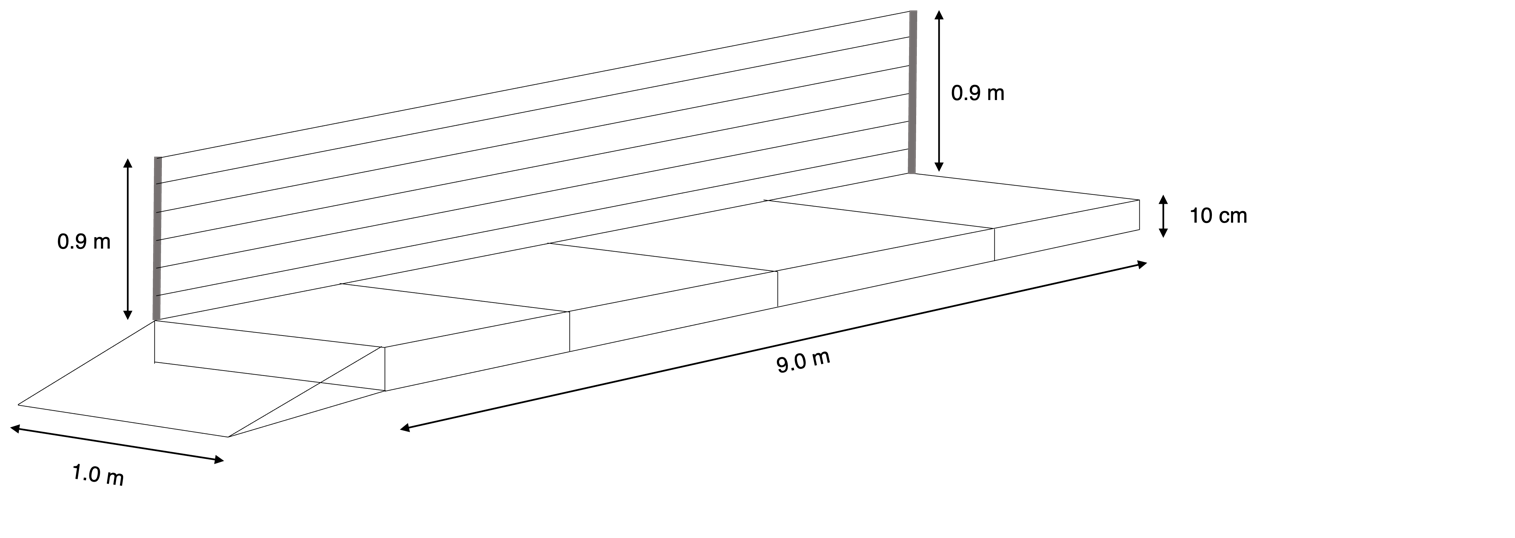


**Supplementary Figure S1. Functional Run Fabrication.** A) The capture area of the run comprised a wooden platform (9.0 m in length × 1.0 m in width × 10 cm in height) over which the animal walked, covered by matte rubber matting (2 ply Natural Insertion Rubber, Clark Rubber, Australia). Ramps on either end allowed smooth transition to/from the platform. The capture area was enclosed with matte, galvanised steel fencing at a height of 0.9 m (Cyclone Ringlock, Australia) comprising six horizontal wires of graduated spacing with a wire diameter of 2.5 mm. B) For the remaining periphery of the run (10 × 5 m), robust, galvanised steel interlocking 1.1 m high fencing was used. The run was enclosed in a large building, with limited external light sources to reduce environmental light for optimised motion capture. Numbered rectangles represent position of each infrared camera (labelled C1 – C10) and video camera (DV1). The final three-dimensional capture volume was 5 m in length × 5 m in width × 2 m in height.

| **Supplementary Table S1. Global outcome measures** | | | | |  |
| --- | --- | --- | --- | --- | --- |
| **Outcome** | **Direction** | **Marker(s) used** | **Variables measured** | **Purpose** | |
| Mean Absolute Velocity T1 | Forward | T1 | Velocity of T1 | Determine velocity of forward movement | |
| Mean Head to T1 | Left/right | HEAD and T1 | Position of the head in relation to T1 | Determine preference for side of the run (left or right) | |
| Mean Head to T1 | Vertical | HEAD and T1 | Position of the head in relation to T1 | Determine lowering of the head | |
| Mean T1 to T13 | Vertical | T1 and T13 | Position of T1 in relation to T13 | Determine lowering of the neck | |
| Mean T1 to L7 | Vertical | T1 and L7 | Position T13 in relation to L7 | Determine lowering of the back | |

| **Supplementary Table S2. Limb-specific outcome measures** | | | | | | | | | |
| --- | --- | --- | --- | --- | --- | --- | --- | --- | --- |
| **Outcome** | | **Direction** | | **Marker(s) used** | | **Variables measured** | | **Purpose** | |
| Stance Duration (s) | | Vertical | | DPHAL (each limb) | | Duration from end of swing to beginning of a new swing cycle | | Determine stance duration | |
| Swing Duration (s) | | Vertical | | DPHAL (each limb) | | Duration from beginning of swing to beginning of stance | | Determine swing duration | |
| Stride Duration (s) | | Vertical | | DPHAL (each limb) | | Duration from entering swing to ending stance | | Determine stride duration | |
| Hoof Lateral Deviation (cm) | | Left/right | | DPHAL (each limb) | | Lateral (outward) deviation of distal limb | |  | |
| Hoof Forward Swing Velocity (m/s) | | Forward | | DPHAL (each limb) | | The speed at which the animal is moving the limb forwards during swing | | The speed (m/s) the animal is lifting or dragging the limb during swing | |
| Hoof Vertical Swing Velocity (m/s) | | Vertical | | DPHAL (each limb) | | The speed at which the animal is lifting the hoof upwards during swing | | The speed (m/s) the animal is lifting or dragging the limb during swing | |
| Range Hoof Height in Swing (cm) | | Vertical | | DPHAL (each limb) | | Range of upwards movement of the hoof during swing | | The amount (cm) the animal is lifting or dragging the limb during swing | |
| Stride Length (cm) | | Vertical | | DPHAL (each limb) | | The length of the stride from stance to swing | | Determine length of stride | |
| Distance Matching Limb during stance (cm) | | Left/right | | Forelimb DPHAL and Hindlimb DPHAL | | Distance between limbs | | Determine splay of limbs pre- and post-stroke | |
| Forelimb Fetlock Angle Stance (º) | | Vertical | | Forelimb PHAL, PPHAL and METAR | | Maximum, minimum and range of joint during stance | | Determine forelimb ipsi- versus contra-lateral lower limb deficit | |
| Forelimb Fetlock Angle in Swing (º) | | Vertical | | Forelimb PHAL, PPHAL and METAR | | Maximum, minimum and range of joint during stance | | Determine forelimb ipsi- versus contra-lateral lower limb deficit | |
| Carpal Angle in Stance (º) | | Vertical | | METAR, PPHAL, LEPIRAD and ULNA | | Maximum, minimum and range of joint during stance | | Determine forelimb ipsi- versus contra-lateral mid limb deficit | |
|  | |  | |  | |  | | (continued) | |
|  | |  | |  | |  | |  | |
|  |  | |  | |  | |  | |  |
| **Outcome** | **Direction** | | **Marker(s) Used** | | **Variables Measured** | | **Purpose** | |  |
| Carpal Angle in Swing (º) | Vertical | | METAR, PPHAL, LEPIRAD and ULNA | | Maximum, minimum and range of joint during stance | | Determine forelimb ipsi- versus contra-lateral mid limb deficit | |  |
| Elbow Angle in Stance (º) | Vertical | | LEPIRAD, ULNA and GTUB | | Maximum, minimum and range of joint during stance | | Determine forelimb ipsi- versus contra-lateral upper limb deficit | |  |
| Elbow Angle in Swing (º) | Vertical | | LEPIRAD, ULNA and GTUB | | Maximum, minimum and range of joint during stance | | Determine forelimb ipsi- versus contra-lateral upper limb deficit | |  |
| Hindlimb Fetlock Angle in Stance (º) | Vertical | | Hindlimb PHAL, PPHAL and FTAR | | Maximum, minimum and range of joint during stance | | Determine hindlimb ipsi- versus contra-lateral lower limb deficit | |  |
| Hindlimb Fetlock Angle in Swing (º) | Vertical | | Hindlimb PHAL, PPHAL and FTAR | | Maximum, minimum and range of joint during stance | | Determine hindlimb ipsi- versus contra-lateral lower limb deficit | |  |
| Tarsal Angle in Stance (º) | Vertical | | FTAR, PPHAL, TIB and LMAL | | Maximum, minimum and range of joint during stance | | Determine hindlimb ipsi- versus contra-lateral mid limb deficit | |  |
| Tarsal Angle in Swing (º) | Vertical | | FTAR, PPHAL, TIB and LMAL | | Maximum, minimum and range of joint during stance | | Determine hindlimb ipsi- versus contra-lateral mid limb deficit | |  |
| Stifle Angle in Stance (º) | Vertical | | LTIB, LMAL, GTROC and LEPI | | Maximum, minimum and range of joint during stance | | Determine hindlimb ipsi- versus contra-lateral upper limb deficit | |  |
| Stifle Angle in Swing (º) | Vertical | | LTIB, LMAL, GTROC and LEPI | | Maximum, minimum and range of joint during stance | | Determine hindlimb ipsi- versus contra-lateral upper limb deficit | |  |
| Ratio Stance to Stride (%) | Vertical | | DPHAL (each limb) | | Ratio of time in stance compared with stride | | Determine ratio of time in stance compared with stride | |  |
| Ratio Swing to Stride (%) | Vertical | | DPHAL (each limb) | | Ratio of time in swing compared with stride | | Determine ratio of time in swing compared with stride | |  |
| Ratio Stance to Swing (%) | Vertical | | DPHAL (each limb) | | Ratio of time in stance compared with swing | | Determine ratio of time in stance compared with swing | |  |

| **Supplementary Table S3. Baseline global parameters (mean (SD))** | | | | | | |
| --- | --- | --- | --- | --- | --- | --- |
| *Variable* | *Baseline 1* | *Baseline 2* | *Baseline 3* | *Mean (SD) all baselines* | *P-value* | *ICC (95% CI)* |
| Mean Absolute Velocity T1 (m/s) | 1.31 (0.17) | 1.26 (0.24) | 1.27 (0.21) | 1.28 (0.20) | 0.409 | 0.41 (0.20, 0.66) |
| Mean Head to T1 (cm) | 2.68 (6.22) | 4.16 (6.26) | 3.35 (5.79) | 3.39 (6.04) | 0.434 | 0.58 (0.36, 0.76) * |
| Mean Head to T1 (cm) | 9.75 (4.59) | 10.41 (3.39) | 9.53 (4.31) | 9.89 (4.10) | 0.317 | 0.70 (0.51, 0.84) * |
| Mean T1 to T13 (cm) | -0.54 (1.14) | -0.43 (1.10) | -0.56 (1.07) | -0.51 (1.09) | 0.625 | 0.74 (0.56, 0.86) * |
| Mean T1 to L7 (cm) | -3.10 (1.49) | -3.04 (1.44) | -3.25 (1.50) | -3.13 (1.46) | 0.416 | 0.80 (0.66, 0.90) * |
| * = ICC >0.50 |  |  |  |  |  |  |

| **Supplementary Table S4. Baseline forelimb parameters (mean (SD))** | | | | | | | |
| --- | --- | --- | --- | --- | --- | --- | --- |
| **Left Forelimb** |  | | | | | | |
| *Variable* | *Baseline 1* | *Baseline 2* | *Baseline 3* | *Mean (SD) all baselines* | *P-value* | *Unadjusted ICC (95% CI)* | *Adjusted ICC (95% CI)* |
| Stance Duration (s) | 0.40 (0.07) | 0.42 (0.09) | 0.41 (0.09) | 0.41 (0.01) | 0.322 | 0.41 (0.20, 0.66) | 0.45 (0.24, 0.69) |
| Swing Duration (s) | 0.35 (0.02) | 0.36 (0.03) | 0.36 (0.02) | 0.36 (0.00) | 0.156 | 0.60 (0.39, 0.78) * | 0.67 (0.47, 0.82) * |
| Stride Duration (s) | 0.75 (0.08) | 0.78 (0.11) | 0.77 (0.11) | 0.77 (0.01) | 0.291 | 0.45 (0.24, 0.69) | 0.59 (0.38, 0.77) * |
| Ratio Stance To Stride (%) | 0.52 (0.04) | 0.53 (0.05) | 0.52 (0.05) | 0.52 (0.01) | 0.369 | 0.36 (0.16, 0.63) | 0.28 (0.09, 0.60) |
| Ratio Swing To Stride (%) | 0.48 (0.04) | 0.47 (0.05) | 0.48 (0.05) | 0.48 (0.01) | 0.369 | 0.36 (0.16, 0.63) | 0.28 (0.09, 0.60) |
| Ratio Stance To Swing (%) | 1.11 (0.18) | 1.16 (0.19) | 1.12 (0.21) | 1.13 (0.02) | 0.385 | 0.34 (0.14, 0.62) | 0.28 (0.10, 0.60) |
| Hoof Lateral Deviation (cm) | 7.29 (1.98) | 7.47 (2.31) | 7.45 (2.59) | 7.40 (0.08) | 0.773 | 0.40 (0.19, 0.65) | 0.41 (0.20, 0.66) |
| Hoof Forward Swing Velocity (m/s) | 2.77 (0.15) | 2.68 (0.25) | 2.71 (0.21) | 2.72 (0.03) | 0.243 | 0.44 (0.22, 0.68) | 0.42 (0.20, 0.67) |
| Hoof Vertical Swing Velocity (m/s) | 0.39 (0.08) | 0.35 (0.08) | 0.36 (0.10) | 0.37 (0.02) | 0.055 | 0.64 (0.44, 0.80) ***** | 0.70 (0.51, 0.84) * |
| Range Hoof Height in Swing (cm) | 7.16 (1.54) | 6.39 (1.59) | 6.63 (2.08) | 6.73 (0.33) | 0.036 | 0.74 (0.56, 0.86) * | 0.78 (0.62, 0.88) * |
| Stride Length (cm) | 98.44 (5.15) | 96.51 (6.17) | 98.64 (6.31) | 97.88 (0.96) | 0.143 | 0.73 (0.56, 0.86) * | 0.82 (0.68, 0.91) * |
| Minimum Forelimb Fetlock Angle Stance (º) | 4.10 (10.78) | 5.82 (8.63) | 5.47 (8.43) | 5.12 (0.75) | 0.449 | 0.56 (0.35, 0.76) * | 0.54 (0.31, 0.75) * |
| Maximum Forelimb Fetlock Angle Stance (º) | 19.51 (10.98) | 21.01 (8.81) | 20.93 (8.56) | 20.47 (0.70) | 0.491 | 0.53 (0.31, 0.74) * | 0.50 (0.27, 0.72) * |
| Range Forelimb Fetlock Angle in Stance (º) | 15.41 (4.18) | 15.18 (3.21) | 15.46 (3.20) | 15.35 (0.12) | 0.856 | 0.63 (0.43, 0.80) * | 0.63 (0.43, 0.80) * |
| Minimum Forelimb Fetlock Angle in Swing (º) | -29.70 (10.51) | -26.87 (7.91) | -27.49 (8.31) | -28.04 (1.22) | 0.233 | 0.43 (0.21, 0.68) | 0.43 (0.21, 0.69) |
| Maximum Forelimb Fetlock Angle in Swing (º) | 12.00 (11.16) | 13.48 (9.42) | 13.08 (8.78) | 12.84 (0.63) | 0.509 | 0.56 (0.35, 0.75) * | 0.52 (0.29, 0.74) * |
| Range Forelimb Fetlock Angle in Swing (º) | 41.70 (6.60) | 40.35 (5.80) | 40.57 (5.63) | 40.88 (0.59) | 0.255 | 0.68 (0.49, 0.83) * | 0.69 (0.49, 0.83) * |
| Minimum Carpal Angle in Stance (º) | -13.90 (4.43) | -14.74 (4.57) | -14.78 (5.10) | -14.47 (0.41) | 0.348 | 0.71 (0.53, 0.84) * | 0.72 (0.54, 0.85) * |
| Maximum Carpal Angle in Stance (º) | -1.74 (4.94) | -2.29 (5.10) | -2.42 (4.90) | -2.15 (0.30) | 0.442 | 0.78 (0.63, 0.89) * | 0.79 (0.63, 0.89) * |
| Range Carpal Angle in Stance (º) | 12.15 (3.49) | 12.45 (3.84) | 12.36 (3.45) | 12.32 (0.12) | 0.759 | 0.68 (0.49, 0.83) * | 0.72 (0.54, 0.85) * |
| Minimum Carpal Angle in Swing (º) | -77.34 (8.01) | -74.86 (8.16) | -77.88 (10.11) | -76.72 (1.32) | 0.109 | 0.75 (0.59, 0.87) * | 0.81 (0.66, 0.90) * |
| Maximum Carpal Angle in Swing (º) | -3.19 (4.75) | -3.96 (5.26) | -4.18 (4.74) | -3.77 (0.43) | 0.243 | 0.81 (0.66, 0.90) * | 0.81 (0.66, 0.90) * |
| Range Carpal Angle in Swing (º) | 74.15 (6.85) | 70.90 (7.44) | 73.71 (8.37) | 72.95 (1.44) | 0.050 | 0.73 (0.55, 0.86) * | 0.82 (0.68, 0.90) * |
| Minimum Elbow Angle in Stance (º) | 51.94 (5.22) | 51.97 (4.71) | 51.92 (5.99) | 51.94 (0.02) | 0.980 | 0.80 (0.65, 0.89) * | 0.85 (0.73, 0.92) * |
| Maximum Elbow Angle in Stance (º) | 76.21 (5.88) | 76.88 (5.93) | 77.23 (5.99) | 76.78 (0.43) | 0.198 | 0.89 (0.80, 0.94) * | 0.89 (0.80, 0.95) * |
| Range Elbow Angle in Stance (º) | 24.27 (3.81) | 24.92 (4.38) | 25.31 (4.49) | 24.83 (0.43) | 0.266 | 0.70 (0.52, 0.84) * | 0.82 (0.69, 0.91) * |
| Minimum Elbow Angle in Swing (º) | 52.92 (5.47) | 53.22 (4.70) | 53.23 (6.07) | 53.12 (0.14) | 0.736 | 0.81 (0.67, 0.90) * | 0.85 (0.74, 0.92) * |
| Maximum Elbow Angle in Swing (º) | 95.89 (6.37) | 94.50 (6.28) | 95.41 (6.75) | 95.27 (0.58) | 0.317 | 0.80 (0.65, 0.89) * | 0.82 (0.68, 0.91) * |
| Range Elbow Angle in Swing (º) | 42.97 (4.60) | 41.28 (5.31) | 42.18 (5.07) | 42.16 (0.69) | 0.175 | 0.74 (0.56, 0.86) * | 0.73 (0.56, 0.86) * |
| * = ICC >0.50 |  |  |  |  |  |  |  |
|  |  |  |  |  |  |  | (continued) |

| **Right Forelimb** |  | | | | | | |
| --- | --- | --- | --- | --- | --- | --- | --- |
| *Variable* | *Baseline 1* | *Baseline 2* | *Baseline 3* | *Mean (SD) all baselines* | *P-value* | *Unadjusted ICC (95% CI)* | *Adjusted ICC (95% CI)* |
| Stance Duration (s) | 0.40 (0.07) | 0.42 (0.10) | 0.42 (0.09) | 0.41 (0.01) | 0.299 | 0.47 (0.25, 0.70) | 0.48 (0.27, 0.71) |
| Swing Duration (s) | 0.36 (0.02) | 0.36 (0.03) | 0.36 (0.02) | 0.36 (0.00) | 0.253 | 0.37 (0.17, 0.64) | 0.53 (0.31, 0.74) * |
| Stride Duration (s) | 0.75 (0.08) | 0.79 (0.12) | 0.78 (0.11) | 0.77 (0.01) | 0.258 | 0.46 (0.24, 0.69) | 0.59 (0.37, 0.77) * |
| Ratio Stance To Stride (%) | 0.52 (0.04) | 0.53 (0.06) | 0.53 (0.05) | 0.52 (0.00) | 0.529 | 0.46 (0.24, 0.69) | 0.30 (0.10, 0.60) |
| Ratio Swing To Stride (%) | 0.48 (0.04) | 0.47 (0.06) | 0.47 (0.05) | 0.48 (0.00) | 0.529 | 0.46 (0.24, 0.69) | 0.30 (0.10, 0.60) |
| Ratio Stance To Swing (%) | 1.11 (0.18) | 1.15 (0.23) | 1.15 (0.21) | 1.13 (0.02) | 0.444 | 0.46 (0.24, 0.69) | 0.30 (0.11, 0.60) |
| Hoof Lateral Deviation (cm) | 7.59 (2.54) | 7.21 (2.98) | 7.07 (2.71) | 7.29 (0.22) | 0.516 | 0.49 (0.28, 0.71) | 0.51 (0.29, 0.72) * |
| Hoof Forward Swing Velocity (m/s) | 2.74 (0.19) | 2.64 (0.23) | 2.71 (0.23) | 2.70 (0.04) | 0.263 | 0.28 (0.10, 0.59) | 0.45 (0.22, 0.69) |
| Hoof Vertical Swing Velocity (m/s) | 0.37 (0.07) | 0.34 (0.07) | 0.36 (0.08) | 0.36 (0.01) | 0.221 | 0.49 (0.27, 0.71) | 0.60 (0.39, 0.78) * |
| Range Hoof Height in Swing (cm) | 6.63 (1.35) | 6.27 (1.37) | 6.54 (1.69) | 6.48 (0.16) | 0.335 | 0.65 (0.45, 0.81) * | 0.70 (0.51, 0.84) * |
| Stride Length (cm) | 97.83 (5.45) | 96.30 (6.12) | 98.14 (6.50) | 97.44 (0.81) | 0.280 | 0.69 (0.50, 0.84) * | 0.80 (0.66, 0.90) * |
| Minimum Forelimb Fetlock Angle Stance (º) | 5.59 (8.26) | 8.40 (8.30) | 5.34 (7.73) | 6.42 (1.39) | 0.199 | 0.61 (0.40, 0.79) * | 0.62 (0.41, 0.79) * |
| Maximum Forelimb Fetlock Angle Stance (º) | 21.03 (9.15) | 23.03 (9.26) | 20.80 (9.11) | 21.60 (1.00) | 0.387 | 0.65 (0.45, 0.81) * | 0.67 (0.48, 0.82) * |
| Range Forelimb Fetlock Angle in Stance (º) | 15.43 (3.79) | 14.62 (3.35) | 15.46 (3.96) | 15.18 (0.39) | 0.319 | 0.74 (0.56, 0.86) * | 0.75 (0.58, 0.87) * |
| Minimum Forelimb Fetlock Angle in Swing (º) | -26.96 (9.28) | -25.34 (10.05) | -25.82 (8.92) | -26.05 (0.68) | 0.453 | 0.73 (0.55, 0.86) * | 0.73 (0.55, 0.86) * |
| Maximum Forelimb Fetlock Angle in Swing (º) | 13.96 (7.68) | 15.59 (8.15) | 13.77 (7.97) | 14.43 (0.82) | 0.465 | 0.57 (0.36, 0.76) * | 0.61 (0.40, 0.79) * |
| Range Forelimb Fetlock Angle in Swing (º) | 40.92 (5.80) | 40.93 (7.05) | 39.59 (7.14) | 40.48 (0.64) | 0.347 | 0.70 (0.51, 0.84) * | 0.70 (0.51, 0.84) * |
| Minimum Carpal Angle in Stance (º) | -16.97 (5.58) | -18.73 (5.75) | -17.46 (5.25) | -17.70 (0.75) | 0.085 | 0.82 (0.68, 0.90) * | 0.84 (0.71, 0.92) * |
| Maximum Carpal Angle in Stance (º) | -3.27 (4.25) | -4.59 (5.02) | -3.87 (4.22) | -3.90 (0.54) | 0.085 | 0.85 (0.73, 0.92) * | 0.84 (0.72, 0.92) * |
| Range Carpal Angle in Stance (º) | 13.70 (3.83) | 14.14 (4.01) | 13.58 (3.61) | 13.80 (0.24) | 0.564 | 0.66 (0.47, 0.82) * | 0.70 (0.52, 0.84) * |
| Minimum Carpal Angle in Swing (º) | -79.86 (7.27) | -78.27 (7.76) | -79.72 (8.40) | -79.30 (0.72) | 0.282 | 0.78 (0.63, 0.88) * | 0.84 (0.72, 0.92) * |
| Maximum Carpal Angle in Swing (º) | -5.16 (4.64) | -6.38 (4.86) | -5.47 (4.73) | -5.66 (0.52) | 0.072 | 0.88 (0.78, 0.94) * | 0.89 (0.80, 0.94) * |
| Range Carpal Angle in Swing (º) | 74.70 (7.10) | 71.89 (6.78) | 74.25 (8.29) | 73.64 (1.23) | 0.092 | 0.71 (0.52, 0.84) * | 0.79 (0.64, 0.89) * |
| Minimum Elbow Angle in Stance (º) | 53.51 (6.59) | 54.30 (6.46) | 53.52 (7.87) | 53.77 (0.37) | 0.388 | 0.76 (0.60, 0.87) * | 0.77 (0.61, 0.88) * |
| Maximum Elbow Angle in Stance (º) | 77.33 (8.49) | 76.95 (8.07) | 77.10 (8.14) | 77.13 (0.16) | 0.858 | 0.85 (0.74, 0.92) * | 0.86 (0.75, 0.93) * |
| Range Elbow Angle in Stance (º) | 23.82 (5.88) | 22.65 (5.57) | 23.58 (4.66) | 23.36 (0.50) | 0.399 | 0.58 (0.36, 0.76) * | 0.61 (0.41, 0.79) * |
| Minimum Elbow Angle in Swing (º) | 54.66 (6.60) | 55.85 (6.54) | 55.14 (7.93) | 55.20 (0.49) | 0.279 | 0.78 (0.62, 0.88) * | 0.79 (0.63, 0.89) * |
| Maximum Elbow Angle in Swing (º) | 96.14 (8.20) | 96.51 (8.16) | 95.47 (8.76) | 96.03 (0.43) | 0.299 | 0.85 (0.73, 0.92) * | 0.85 (0.74, 0.92) * |
| Range Elbow Angle in Swing (º) | 41.48 (5.58) | 40.67 (6.52) | 40.33 (5.20) | 40.83 (0.49) | 0.386 | 0.69 (0.50, 0.83) * | 0.69 (0.50, 0.83) * |
| Distance Matching Limb during stance (cm) | 51.12 (2.71) | 50.98 (3.12) | 51.73 (2.59) | 51.28 (0.33) | 0.401 | 0.56 (0.34, 0.76) * | 0.52 (0.29, 0.74) * |
|  |  |  |  |  |  |  |  |
|  |  |  |  |  |  |  |  |

| **Supplementary Table S5. Baseline hindlimb parameters (mean (SD))** | | | | | | | |
| --- | --- | --- | --- | --- | --- | --- | --- |
| **Left Hindlimb** |  |  |  |  |  |  |  |
| *Variable* | *Baseline 1* | *Baseline 2* | *Baseline 3* | *Mean (SD) all baselines* | *P-value* | *Unadjusted ICC (95% CI)* | *Adjusted ICC (95% CI)* |
| Stance Duration (s) | 0.42 (0.07) | 0.44 (0.10) | 0.43 (0.09) | 0.43 (0.01) | 0.371 | 0.43 (0.22, 0.68) | 0.47 (0.24, 0.70) |
| Swing Duration (s) | 0.34 (0.03) | 0.34 (0.03) | 0.34 (0.03) | 0.34 (0.00) | 0.604 | 0.54 (0.33, 0.74) * | 0.66 (0.47, 0.82) * |
| Stride Duration (s) | 0.76 (0.09) | 0.78 (0.12) | 0.77 (0.11) | 0.77 (0.01) | 0.431 | 0.46 (0.24, 0.69) | 0.62 (0.41, 0.79) * |
| Ratio Stance To Stride (%) | 0.55 (0.04) | 0.56 (0.04) | 0.55 (0.05) | 0.55 (0.00) | 0.394 | 0.42 (0.20, 0.67) | 0.35 (0.14, 0.64) |
| Ratio Swing To Stride (%) | 0.45 (0.04) | 0.44 (0.04) | 0.45 (0.05) | 0.45 (0.00) | 0.394 | 0.42 (0.20, 0.67) | 0.35 (0.14, 0.64) |
| Ratio Stance To Swing (%) | 1.24 (0.19) | 1.29 (0.20) | 1.25 (0.22) | 1.26 (0.02) | 0.349 | 0.40 (0.19, 0.65) | 0.32 (0.12, 0.62) |
| Hoof Lateral Deviation (cm) | 7.66 (2.86) | 6.86 (2.12) | 6.68 (2.04) | 7.07 (0.43) | 0.281 | 0.12 (0.01, 0.61) | 0.11 (0.01, 0.63) |
| Hoof Forward Swing Velocity (m/s) | 2.89 (0.21) | 2.83 (0.31) | 2.88 (0.27) | 2.87 (0.03) | 0.616 | 0.39 (0.18, 0.65) | 0.66 (0.46, 0.82) * |
| Hoof Vertical Swing Velocity (m/s) | 0.25 (0.06) | 0.24 (0.06) | 0.24 (0.07) | 0.24 (0.01) | 0.438 | 0.50 (0.28, 0.72) * | 0.67 (0.48, 0.83) * |
| Range Hoof Height in Swing (cm) | 4.35 (0.97) | 4.10 (0.94) | 4.10 (1.09) | 4.18 (0.12) | 0.300 | 0.61 (0.40, 0.79) * | 0.72 (0.54, 0.85) * |
| Stride Length (cm) | 97.90 (5.99) | 96.33 (5.93) | 98.72 (5.77) | 97.67 (0.99) | 0.224 | 0.62 (0.41, 0.79) * | 0.73 (0.54, 0.85) * |
| Minimum Hindlimb Fetlock Angle Stance (º) | 3.68 (8.29) | 0.67 (6.97) | 1.96 (7.31) | 2.12 (1.24) | 0.186 | 0.53 (0.31, 0.73) * | 0.52 (0.31, 0.73) * |
| Maximum Hindlimb Fetlock Angle Stance (º) | 20.41 (9.09) | 17.60 (8.82) | 18.98 (8.00) | 19.02 (1.15) | 0.273 | 0.56 (0.34, 0.75) * | 0.56 (0.35, 0.76) * |
| Range Hindlimb Fetlock Angle in Stance (º) | 16.73 (5.44) | 16.93 (4.79) | 17.02 (4.99) | 16.90 (0.12) | 0.788 | 0.69 (0.50, 0.83) * | 0.69 (0.50, 0.83) * |
| Minimum Hindlimb Fetlock Angle in Swing (º) | -31.51 (8.65) | -33.72 (6.22) | -30.96 (7.49) | -32.04 (1.19) | 0.191 | 0.53 (0.31, 0.73) * | 0.55 (0.33, 0.75) * |
| Maximum Hindlimb Fetlock Angle in Swing (º) | 10.58 (7.78) | 7.33 (9.01) | 9.35 (7.13) | 9.11 (1.34) | 0.180 | 0.52 (0.31, 0.73) * | 0.56 (0.34, 0.75) * |
| Range Hindlimb Fetlock Angle in Swing (º) | 42.09 (6.22) | 41.05 (8.01) | 40.31 (6.26) | 41.15 (0.74) | 0.378 | 0.50 (0.28, 0.72) * | 0.67 (0.47, 0.82) * |
| Minimum Tarsal Angle in Stance (º) | 28.01 (7.37) | 27.95 (6.83) | 27.14 (8.61) | 27.70 (0.40) | 0.490 | 0.84 (0.71, 0.91) * | 0.83 (0.70, 0.91) * |
| Maximum Tarsal Angle in Stance (º) | 63.41 (6.57) | 62.82 (7.05) | 63.08 (9.01) | 63.10 (0.24) | 0.687 | 0.73 (0.56, 0.86) * | 0.75 (0.58, 0.87) * |
| Range Tarsal Angle in Stance (º) | 35.40 (5.51) | 34.86 (5.33) | 35.94 (5.51) | 35.41 (0.44) | 0.609 | 0.72 (0.54, 0.85) * | 0.75 (0.58, 0.87) * |
| Minimum Tarsal Angle in Swing (º) | 28.01 (7.37) | 27.98 (6.82) | 27.20 (8.59) | 27.73 (0.38) | 0.504 | 0.84 (0.71, 0.92) * | 0.84 (0.71, 0.92) * |
| Maximum Tarsal Angle in Swing (º) | 77.93 (6.84) | 77.24 (7.98) | 76.59 (9.09) | 77.25 (0.56) | 0.510 | 0.61 (0.40, 0.78) * | 0.73 (0.54, 0.85) * |
| Range Tarsal Angle in Swing (º) | 49.92 (5.15) | 49.25 (7.25) | 49.39 (6.95) | 49.53 (0.29) | 0.779 | 0.51 (0.29, 0.72) * | 0.64 (0.43, 0.80) * |
| Minimum Stifle Angle in Stance (º) | -69.42 (14.76) | -68.28 (14.05) | -67.92 (14.30) | -68.54 (0.65) | 0.513 | 0.85 (0.73, 0.92) * | 0.85 (0.74, 0.92) * |
| Maximum Stifle Angle in Stance (º) | -46.58 (13.23) | -44.31 (14.18) | -44.44 (14.87) | -45.12 (1.05) | 0.351 | 0.81 (0.66, 0.90) * | 0.84 (0.71, 0.92) * |
| Range Stifle Angle in Stance (º) | 22.85 (4.81) | 23.96 (4.58) | 23.48 (5.27) | 23.42 (0.46) | 0.382 | 0.61 (0.40, 0.78) * | 0.60 (0.39, 0.78) * |
| Minimum Stifle Angle in Swing (º) | -81.80 (13.70) | -80.80 (13.65) | -79.57 (15.07) | -80.72 (0.93) | 0.400 | 0.79 (0.64, 0.89) * | 0.85 (0.73, 0.92) * |
| Maximum Stifle Angle in Swing (º) | -37.38 (14.23) | -34.78 (13.99) | -34.18 (14.51) | -35.46 (1.41) | 0.162 | 0.84 (0.72, 0.92) * | 0.85 (0.73, 0.92) * |
| Range Stifle Angle in Swing (º) | 44.42 (5.04) | 46.02 (6.55) | 45.39 (4.84) | 45.26 (0.66) | 0.360 | 0.52 (0.30, 0.73) * | 0.71 (0.51, 0.85) * |
| * = ICC >0.50 |  |  |  |  |  |  |  |
|  |  |  |  |  |  |  | (continued) |
|  |  | | | | | | |
| **Right Hindlimb** |  |  |  |  |  |  |  |
| *Variable* | *Baseline 1* | *Baseline 2* | *Baseline 3* | *Mean (SD) all baselines* | *P-value* | *Unadjusted ICC (95% CI)* | *Adjusted ICC (95% CI)* |
| Stance Duration (s) | 0.42 (0.07) | 0.44 (0.09) | 0.44 (0.10) | 0.43 (0.01) | 0.418 | 0.46 (0.24, 0.69) | 0.49 (0.27, 0.71) |
| Swing Duration (s) | 0.34 (0.03) | 0.34 (0.03) | 0.34 (0.03) | 0.34 (0.00) | 0.476 | 0.58 (0.37, 0.77) * | 0.69 (0.50, 0.83) * |
| Stride Duration (s) | 0.76 (0.09) | 0.78 (0.11) | 0.78 (0.11) | 0.77 (0.01) | 0.390 | 0.47 (0.25, 0.70) | 0.62 (0.41, 0.79) * |
| Ratio Stance To Stride (%) | 0.55 (0.04) | 0.55 (0.05) | 0.55 (0.06) | 0.55 (0.00) | 0.583 | 0.49 (0.27, 0.71) | 0.48 (0.26, 0.71) |
| Ratio Swing To Stride (%) | 0.45 (0.04) | 0.45 (0.05) | 0.45 (0.06) | 0.45 (0.00) | 0.583 | 0.49 (0.27, 0.71) | 0.48 (0.26, 0.71) |
| Ratio Stance To Swing (%) | 1.24 (0.19) | 1.28 (0.22) | 1.28 (0.28) | 1.27 (0.02) | 0.524 | 0.44 (0.22, 0.68) | 0.41 (0.19, 0.66) |
| Hoof Lateral Deviation (cm) | 7.74 (1.59) | 7.71 (2.06) | 7.25 (2.13) | 7.56 (0.23) | 0.327 | 0.33 (0.13, 0.63) | 0.30 (0.10, 0.62) |
| Hoof Forward Swing Velocity (m/s) | 2.90 (0.22) | 2.83 (0.29) | 2.86 (0.23) | 2.86 (0.03) | 0.433 | 0.40 (0.19, 0.66) | 0.68 (0.48, 0.83) * |
| Hoof Vertical Swing Velocity (m/s) | 0.23 (0.06) | 0.22 (0.06) | 0.22 (0.06) | 0.22 (0.00) | 0.702 | 0.59 (0.37, 0.78) * | 0.80 (0.65, 0.90) * |
| Range Hoof Height in Swing (cm) | 3.96 (0.83) | 3.71 (0.78) | 3.87 (0.98) | 3.85 (0.10) | 0.535 | 0.61 (0.40, 0.79) * | 0.76 (0.59, 0.88) * |
| Stride Length (cm) | 98.04 (5.69) | 96.88 (6.02) | 97.94 (6.27) | 97.63 (0.52) | 0.595 | 0.66 (0.47, 0.82) * | 0.74 (0.56, 0.86) * |
| Minimum Hindlimb Fetlock Angle Stance (º) | 3.50 (8.58) | 0.50 (6.64) | 2.66 (7.61) | 2.25 (1.26) | 0.126 | 0.60 (0.39, 0.78) * | 0.60 (0.39, 0.78) * |
| Maximum Hindlimb Fetlock Angle Stance (º) | 20.68 (9.73) | 18.10 (7.16) | 20.44 (9.23) | 19.77 (1.16) | 0.156 | 0.67 (0.47, 0.82) * | 0.67 (0.47, 0.82) * |
| Range Hindlimb Fetlock Angle in Stance (º) | 17.19 (4.96) | 17.60 (3.84) | 17.78 (4.56) | 17.52 (0.25) | 0.526 | 0.75 (0.58, 0.87) * | 0.75 (0.58, 0.87) * |
| Minimum Hindlimb Fetlock Angle in Swing (º) | -31.36 (7.82) | -31.77 (6.67) | -29.69 (7.32) | -30.93 (0.91) | 0.346 | 0.39 (0.18, 0.65) | 0.48 (0.25, 0.71) |
| Maximum Hindlimb Fetlock Angle in Swing (º) | 10.82 (9.34) | 8.19 (6.77) | 11.28 (8.74) | 10.12 (1.36) | 0.095 | 0.66 (0.46, 0.81) * | 0.66 (0.46, 0.82) * |
| Range Hindlimb Fetlock Angle in Swing (º) | 42.18 (6.70) | 39.96 (7.76) | 40.98 (7.26) | 41.05 (0.91) | 0.212 | 0.63 (0.43, 0.80) * | 0.79 (0.64, 0.89) * |
| Minimum Tarsal Angle in Stance (º) | 24.61 (6.60) | 24.46 (7.20) | 24.23 (7.05) | 24.43 (0.16) | 0.646 | 0.77 (0.60, 0.88) * | 0.77 (0.60, 0.88) * |
| Maximum Tarsal Angle in Stance (º) | 59.87 (7.37) | 61.04 (8.54) | 60.60 (7.43) | 60.50 (0.48) | 0.630 | 0.65 (0.44, 0.81) * | 0.67 (0.47, 0.82) * |
| Range Tarsal Angle in Stance (º) | 35.26 (5.16) | 36.58 (5.63) | 36.37 (5.36) | 36.06 (0.58) | 0.161 | 0.76 (0.60, 0.87) * | 0.75 (0.58, 0.87) * |
| Minimum Tarsal Angle in Swing (º) | 24.63 (6.41) | 24.68 (7.12) | 24.38 (7.11) | 24.56 (0.13) | 0.749 | 0.76 (0.59, 0.87) * | 0.76 (0.58, 0.87) * |
| Maximum Tarsal Angle in Swing (º) | 73.76 (6.84) | 74.52 (8.96) | 74.23 (8.00) | 74.17 (0.32) | 0.805 | 0.62 (0.41, 0.79) * | 0.69 (0.50, 0.84) * |
| Range Tarsal Angle in Swing (º) | 49.13 (4.90) | 49.85 (6.63) | 49.85 (5.74) | 49.61 (0.34) | 0.503 | 0.68 (0.49, 0.83) * | 0.72 (0.53, 0.85) * |
| Minimum Stifle Angle in Stance (º) | -71.48 (13.23) | -71.00 (14.49) | -70.55 (13.52) | -71.01 (0.39) | 0.566 | 0.91 (0.84, 0.96) * | 0.91 (0.84, 0.96) * |
| Maximum Stifle Angle in Stance (º) | -48.67 (11.71) | -47.69 (12.99) | -47.31 (13.78) | -47.89 (0.58) | 0.509 | 0.84 (0.71, 0.92) * | 0.88 (0.77, 0.94) * |
| Range Stifle Angle in Stance (º) | 22.81 (4.62) | 23.31 (5.23) | 23.24 (5.03) | 23.12 (0.22) | 0.597 | 0.62 (0.41, 0.79) * | 0.67 (0.47, 0.82) * |
| Minimum Stifle Angle in Swing (º) | -84.25 (11.65) | -83.32 (14.27) | -82.52 (14.29) | -83.36 (0.72) | 0.395 | 0.87 (0.76, 0.93) * | 0.91 (0.83, 0.95) * |
| Maximum Stifle Angle in Swing (º) | -39.31 (11.24) | -38.23 (12.90) | -37.47 (12.59) | -38.34 (0.77) | 0.320 | 0.87 (0.76, 0.93) * | 0.87 (0.76, 0.93) * |
| Range Stifle Angle in Swing (º) | 44.94 (5.18) | 45.09 (6.13) | 45.05 (6.71) | 45.03 (0.06) | 0.909 | 0.64 (0.44, 0.80) * | 0.73 (0.55, 0.85) * |
| Distance Matching Limb during stance (cm) | 89.01 (6.85) | 88.73 (8.15) | 89.58 (9.63) | 89.11 (0.36) | 0.631 | 0.59 (0.38, 0.77) * | 0.70 (0.51, 0.84) * |
| * = ICC >0.50 |  |  |  |  |  |  |  |

| **Supplementary Table S6. Baseline compared to post-stroke global parameters (mean (SD))** | | | | |
| --- | --- | --- | --- | --- |
| *Variable* | *Mean (SD) all baselines* | *Post-stroke* | *Mean difference (95% CI)* | *P-value* |
| Mean Absolute Velocity T1 (m/s) | 1.28 (0.20) | 1.00 (0.18) | -0.28 (-0.35, -0.22) | <0.001 * |
| Mean Head to T1 (cm) | 3.39 (6.04) | 0.61 (4.79) | -2.72 (-5.42, -0.02) | 0.049 * |
| Mean Head to T1 (cm) | 9.89 (4.10) | 1.69 (7.29) | -8.21 (-10.73, -5.68) | <0.001 * |
| Mean T1 to T13 (cm) | -0.51 (1.09) | -2.40 (1.56) | -1.88 (-2.47, -1.30) | <0.001 * |
| Mean T1 to L7 (cm) | -3.13 (1.46) | -5.40 (1.82) | -2.27 (-2.88, -1.67) | <0.001 * |
| * = *p*<0.05 |  |  |  |  |

| **Supplementary Table S7. Baseline compared to post-stroke fore-limb parameters (mean (SD))** | | | | | | |  |
| --- | --- | --- | --- | --- | --- | --- | --- |
| **Left Forelimb** |  | |  |  |  |  |  |
| *Variable* | *Mean all baselines* | *Post-stroke* | *Mean difference un-adjusted (95% CI)* | *P-value un-adjusted* | *Mean difference adjusted (95% CI)* | *P-value adjusted* |  |
| Stance Duration (s) | 0.41 (0.01) | 0.54 (0.11) | 0.13 (0.11, 0.16) | <0.001 * | 0.02 (0.00, 0.05) | 0.037 * |  |
| Swing Duration (s) | 0.36 (0.00) | 0.38 (0.04) | 0.02 (0.01, 0.03) | <0.001 * | 0.00 (-0.01, 0.01) | 0.851 |  |
| Stride Duration (s) | 0.77 (0.01) | 0.92 (0.13) | 0.15 (0.12, 0.18) | <0.001 * | 0.03 (0.00, 0.05) | 0.028 * |  |
| Ratio Stance To Stride (%) | 0.52 (0.01) | 0.58 (0.04) | 0.06 (0.05, 0.07) | <0.001 * | 0.02 (0.00, 0.03) | 0.018 * |  |
| Ratio Swing To Stride (%) | 0.48 (0.01) | 0.42 (0.04) | -0.06 (-0.07, -0.05) | <0.001 * | -0.02 (-0.03, -0.00) | 0.018 * |  |
| Ratio Stance To Swing (%) | 1.13 (0.02) | 1.44 (0.23) | 0.31 (0.24, 0.39) | <0.001 * | 0.06 (-0.01, 0.14) | 0.096 |  |
| Hoof Lateral Deviation (cm) | 7.40 (0.08) | 6.39 (2.04) | -1.02 (-1.88, -0.16) | 0.020 * | -1.10 (-2.13, -0.07) | 0.036 * |  |
| Hoof Forward Swing Velocity (m/s) | 2.72 (0.03) | 2.42 (0.25) | -0.31 (-0.38, -0.23) | <0.001 * | -0.05 (-0.12, 0.03) | 0.240 |  |
| Hoof Vertical Swing Velocity (m/s) | 0.37 (0.02) | 0.32 (0.09) | -0.04 (-0.07, -0.02) | 0.003 * | -0.03 (-0.07, 0.00) | 0.061 |  |
| Range Hoof Height in Swing (cm) | 6.73 (0.33) | 6.10 (1.89) | -0.62 (-1.20, -0.03) | 0.038 * | -0.56 (-1.27, 0.15) | 0.124 |  |
| Stride Length (cm) | 97.88 (0.96) | 90.60 (5.41) | -7.41 (-9.25, -5.58) | <0.001 * | -1.50 (-3.34, 0.33) | 0.109 |  |
| Minimum Forelimb Fetlock Angle Stance (º) | 5.12 (0.75) | 4.16 (9.73) | -1.01 (-5.06, 3.03) | 0.624 | 0.57 (-4.09, 5.24) | 0.810 |  |
| Maximum Forelimb Fetlock Angle Stance (º) | 20.47 (0.70) | 19.73 (9.89) | -0.83 (-4.90, 3.24) | 0.689 | -0.38 (-5.21, 4.46) | 0.879 |  |
| Range Forelimb Fetlock Angle in Stance (º) | 15.35 (0.12) | 15.57 (4.10) | 0.18 (-1.60, 1.96) | 0.841 | -0.77 (-2.94, 1.39) | 0.485 |  |
| Minimum Forelimb Fetlock Angle in Swing (º) | -28.04 (1.22) | -24.69 (11.23) | 3.17 (-0.96, 7.30) | 0.132 | 3.26 (-1.54, 8.05) | 0.183 |  |
| Maximum Forelimb Fetlock Angle in Swing (º) | 12.84 (0.63) | 12.21 (9.22) | -0.73 (-4.74, 3.28) | 0.721 | 0.94 (-3.76, 5.63) | 0.696 |  |
| Range Forelimb Fetlock Angle in Swing (º) | 40.88 (0.59) | 36.90 (7.81) | -3.90 (-6.62, -1.18) | 0.005 * | -2.17 (-5.45, 1.11) | 0.195 |  |
| Minimum Carpal Angle in Stance (º) | -14.47 (0.41) | -15.47 (4.77) | -1.01 (-3.98, 1.96) | 0.506 | -0.98 (-4.49, 2.53) | 0.584 |  |
| Maximum Carpal Angle in Stance (º) | -2.15 (0.30) | -4.31 (4.91) | -2.15 (-4.85, 0.54) | 0.118 | -0.49 (-3.77, 2.79) | 0.769 |  |
| Range Carpal Angle in Stance (º) | 12.32 (0.12) | 11.16 (2.99) | -1.15 (-3.24, 0.95) | 0.283 | 0.55 (-1.91, 3.01) | 0.663 |  |
| Minimum Carpal Angle in Swing (º) | -76.72 (1.32) | -75.37 (6.58) | 1.42 (-1.71, 4.54) | 0.374 | 0.70 (-3.10, 4.51) | 0.717 |  |
| Maximum Carpal Angle in Swing (º) | -3.77 (0.43) | -5.18 (5.30) | -1.40 (-4.54, 1.74) | 0.383 | -0.00 (-3.79, 3.78) | 0.999 |  |
| Range Carpal Angle in Swing (º) | 72.95 (1.44) | 70.19 (6.41) | -2.81 (-5.58, -0.05) | 0.046 * | -0.61 (-3.93, 2.71) | 0.719 |  |
| Minimum Elbow Angle in Stance (º) | 51.94 (0.02) | 57.92 (6.73) | 6.00 (0.55, 11.45) | 0.031 * | 4.84 (-1.64, 11.33) | 0.143 |  |
| Maximum Elbow Angle in Stance (º) | 76.78 (0.43) | 81.98 (6.87) | 5.18 (-0.07, 10.43) | 0.053 * | 2.08 (-4.06, 8.22) | 0.507 |  |
| Range Elbow Angle in Stance (º) | 24.83 (0.43) | 24.05 (3.86) | -0.82 (-3.21, 1.57) | 0.503 | -3.03 (-5.81, -0.25) | 0.033 * |  |
| Minimum Elbow Angle in Swing (º) | 53.12 (0.14) | 58.82 (6.46) | 5.71 (0.54, 10.87) | 0.030 * | 3.50 (-2.66, 9.66) | 0.265 |  |
| Maximum Elbow Angle in Swing (º) | 95.27 (0.58) | 100.21 (7.49) | 4.88 (-0.49, 10.25) | 0.075 | 2.92 (-3.40, 9.23) | 0.365 |  |
| Range Elbow Angle in Swing (º) | 42.16 (0.69) | 41.40 (4.61) | -0.83 (-3.12, 1.46) | 0.479 | -0.91 (-3.67, 1.85) | 0.518 |  |
| * = *p*<0.05 |  |  |  |  |  |  |  |
|  |  |  |  |  | (continued) | |  |
|  |  | | | | | | |
| **Right forelimb** |  |  |  |  |  |  |  |
| *Variable* | *Mean all baselines* | *Post-stroke* | *Mean difference un-adjusted (95% CI)* | *P-value un-adjusted* | *Mean difference adjusted (95% CI)* | *P-value adjusted* |  |
| Stance Duration (s) | 0.41 (0.01) | 0.55 (0.11) | 0.14 (0.11, 0.16) | <0.001 * | 0.03 (0.00, 0.05) | 0.020 * |  |
| Swing Duration (s) | 0.36 (0.00) | 0.38 (0.02) | 0.02 (0.01, 0.02) | 0.002 * | -0.00 (-0.01, 0.01) | 0.567 |  |
| Stride Duration (s) | 0.77 (0.01) | 0.92 (0.13) | 0.15 (0.12, 0.18) | <0.001 * | 0.03 (0.00, 0.05) | 0.039 * |  |
| Ratio Stance To Stride (%) | 0.52 (0.00) | 0.59 (0.04) | 0.06 (0.05, 0.08) | <0.001 * | 0.02 (0.01, 0.04) | 0.004 * |  |
| Ratio Swing To Stride (%) | 0.48 (0.00) | 0.41 (0.04) | -0.06 (-0.08, -0.05) | <0.001 * | -0.02 (-0.04, -0.01) | 0.004 * |  |
| Ratio Stance To Swing (%) | 1.13 (0.02) | 1.47 (0.28) | 0.33 (0.25, 0.41) | <0.001 * | 0.08 (0.01, 0.16) | 0.033 * |  |
| Hoof Lateral Deviation (cm) | 7.29 (0.22) | 5.88 (1.57) | -1.43 (-2.29, -0.57) | 0.001 * | -1.51 (-2.54, -0.48) | 0.004 * |  |
| Hoof Forward Swing Velocity (m/s) | 2.70 (0.04) | 2.41 (0.18) | -0.30 (-0.37, -0.22) | <0.001 * | -0.03 (-0.11, 0.04) | 0.363 |  |
| Hoof Vertical Swing Velocity (m/s) | 0.36 (0.01) | 0.30 (0.06) | -0.05 (-0.08, -0.02) | <0.001 * | -0.04 (-0.08, -0.01) | 0.023 * |  |
| Range Hoof Height in Swing (cm) | 6.48 (0.16) | 5.71 (1.20) | -0.78 (-1.36, -0.19) | 0.009 * | -0.72 (-1.43, -0.01) | 0.048 * |  |
| Stride Length (cm) | 97.44 (0.81) | 90.52 (5.57) | -7.05 (-8.88, -5.22) | <0.001 * | -1.14 (-2.98, 0.70) | 0.224 |  |
| Minimum Forelimb Fetlock Angle Stance (º) | 6.42 (1.39) | 5.87 (8.97) | -0.37 (-4.42, 3.67) | 0.856 | 1.21 (-3.46, 5.88) | 0.611 |  |
| Maximum Forelimb Fetlock Angle Stance (º) | 21.60 (1.00) | 21.57 (9.46) | 0.11 (-3.96, 4.17) | 0.959 | 0.56 (-4.27, 5.40) | 0.820 |  |
| Range Forelimb Fetlock Angle in Stance (º) | 15.18 (0.39) | 15.71 (4.29) | 0.48 (-1.30, 2.26) | 0.596 | -0.47 (-2.64, 1.69) | 0.669 |  |
| Minimum Forelimb Fetlock Angle in Swing (º) | -26.05 (0.68) | -23.78 (8.87) | 2.32 (-1.80, 6.45) | 0.270 | 2.41 (-2.39, 7.20) | 0.325 |  |
| Maximum Forelimb Fetlock Angle in Swing (º) | 14.43 (0.82) | 13.32 (9.07) | -0.99 (-5.00, 3.02) | 0.629 | 0.68 (-4.02, 5.38) | 0.777 |  |
| Range Forelimb Fetlock Angle in Swing (º) | 40.48 (0.64) | 37.10 (6.51) | -3.31 (-6.03, -0.60) | 0.017 * | -1.58 (-4.86, 1.70) | 0.345 |  |
| Minimum Carpal Angle in Stance (º) | -17.70 (0.75) | -17.55 (4.79) | 0.13 (-2.84, 3.10) | 0.933 | 0.15 (-3.36, 3.67) | 0.931 |  |
| Maximum Carpal Angle in Stance (º) | -3.90 (0.54) | -5.18 (3.49) | -1.28 (-3.98, 1.41) | 0.351 | 0.38 (-2.90, 3.66) | 0.821 |  |
| Range Carpal Angle in Stance (º) | 13.80 (0.24) | 12.37 (3.32) | -1.41 (-3.50, 0.68) | 0.186 | 0.28 (-2.18, 2.74) | 0.822 |  |
| Minimum Carpal Angle in Swing (º) | -79.30 (0.72) | -76.25 (6.76) | 3.08 (-0.05, 6.20) | 0.054 | 2.36 (-1.44, 6.17) | 0.224 |  |
| Maximum Carpal Angle in Swing (º) | -5.66 (0.52) | -6.14 (3.72) | -0.47 (-3.61, 2.66) | 0.767 | 0.92 (-2.87, 4.71) | 0.634 |  |
| Range Carpal Angle in Swing (º) | 73.64 (1.23) | 70.12 (5.74) | -3.55 (-6.31, -0.79) | 0.012 * | -1.35 (-4.67, 1.97) | 0.427 |  |
| Minimum Elbow Angle in Stance (º) | 53.77 (0.37) | 56.38 (9.20) | 2.52 (-2.93, 7.97) | 0.366 | 1.36 (-5.12, 7.85) | 0.681 |  |
| Maximum Elbow Angle in Stance (º) | 77.13 (0.16) | 81.20 (8.27) | 3.98 (-1.27, 9.23) | 0.137 | 0.88 (-5.26, 7.02) | 0.778 |  |
| Range Elbow Angle in Stance (º) | 23.36 (0.50) | 24.82 (7.63) | 1.46 (-0.93, 3.86) | 0.230 | -0.75 (-3.53, 2.03) | 0.598 |  |
| Minimum Elbow Angle in Swing (º) | 55.20 (0.49) | 57.95 (8.95) | 2.65 (-2.51, 7.82) | 0.314 | 0.45 (-5.71, 6.60) | 0.887 |  |
| Maximum Elbow Angle in Swing (º) | 96.03 (0.43) | 99.29 (8.73) | 3.14 (-2.23, 8.51) | 0.252 | 1.17 (-5.14, 7.49) | 0.716 |  |
| Range Elbow Angle in Swing (º) | 40.83 (0.49) | 41.34 (7.18) | 0.49 (-1.80, 2.78) | 0.677 | 0.40 (-2.36, 3.16) | 0.775 |  |
| Distance Matching Limb during stance (cm) | 51.28 (0.33) | 47.51 (2.87) | -3.84 (-5.92, -1.77) | <0.001 * | -0.62 (-2.92, 1.67) | 0.595 |  |
| * = *p*<0.05 |  |  |  |  |  |  |  |

| **Supplementary Table S8. Baseline compared to post-stroke hind-limb parameters (mean (SD))** | | | | | | |
| --- | --- | --- | --- | --- | --- | --- |
| **Left hindlimb** |  | | | | | |
| *Variable* | *Mean all baselines* | *Post-stroke* | *Mean difference un-adjusted (95% CI)* | *P-value un-adjusted* | *Mean difference adjusted (95% CI)* | *P-value adjusted* |
| Stance Duration (s) | 0.43 (0.01) | 0.56 (0.11) | 0.13 (0.10, 0.15) | <0.001 * | 0.02 (-0.01, 0.04) | 0.132 |
| Swing Duration (s) | 0.34 (0.00) | 0.37 (0.04) | 0.03 (0.02, 0.04) | <0.001 * | 0.01 (-0.00, 0.02) | 0.061 |
| Stride Duration (s) | 0.77 (0.01) | 0.93 (0.13) | 0.16 (0.13, 0.19) | <0.001 * | 0.03 (0.01, 0.05) | 0.015 * |
| Ratio Stance To Stride (%) | 0.55 (0.00) | 0.60 (0.03) | 0.05 (0.03, 0.06) | <0.001 * | 0.00 (-0.01, 0.02) | 0.599 |
| Ratio Swing To Stride (%) | 0.45 (0.00) | 0.40 (0.03) | -0.05 (-0.06, -0.03) | <0.001 * | -0.00 (-0.02, 0.01) | 0.599 |
| Ratio Stance To Swing (%) | 1.26 (0.02) | 1.51 (0.22) | 0.25 (0.17, 0.32) | <0.001 * | -0.00 (-0.08, 0.07) | 0.962 |
| Hoof Lateral Deviation (cm) | 7.07 (0.43) | 6.55 (1.97) | -0.55 (-1.41, 0.31) | 0.208 | -0.63 (-1.66, 0.40) | 0.231 |
| Hoof Forward Swing Velocity (m/s) | 2.87 (0.03) | 2.45 (0.27) | -0.43 (-0.50, -0.35) | <0.001 * | -0.17 (-0.24, -0.09) | <0.001 * |
| Hoof Vertical Swing Velocity (m/s) | 0.24 (0.01) | 0.21 (0.08) | -0.03 (-0.06, 0.00) | 0.056 | -0.02 (-0.05, 0.02) | 0.319 |
| Range Hoof Height in Swing (cm) | 4.18 (0.12) | 3.97 (1.35) | -0.22 (-0.80, 0.37) | 0.469 | -0.16 (-0.87, 0.55) | 0.666 |
| Stride Length (cm) | 97.67 (0.99) | 90.13 (6.06) | -7.70 (-9.53, -5.87) | <0.001 * | -1.79 (-3.63, 0.05) | 0.056 |
| Minimum Hindlimb Fetlock Angle Stance (º) | 2.12 (1.24) | 1.81 (7.61) | -0.35 (-4.39, 3.70) | 0.867 | 1.24 (-3.43, 5.91) | 0.603 |
| Maximum Hindlimb Fetlock Angle Stance (º) | 19.02 (1.15) | 17.28 (7.29) | -1.79 (-5.85, 2.28) | 0.389 | -1.33 (-6.17, 3.50) | 0.589 |
| Range Hindlimb Fetlock Angle in Stance (º) | 16.90 (0.12) | 15.47 (4.24) | -1.44 (-3.22, 0.34) | 0.112 | -2.39 (-4.56, -0.23) | 0.03 * |
| Minimum Hindlimb Fetlock Angle in Swing (º) | -32.04 (1.19) | -25.68 (8.63) | 6.35 (2.22, 10.47) | 0.003 * | 6.43 (1.64, 11.23) | 0.009 * |
| Maximum Hindlimb Fetlock Angle in Swing (º) | 9.11 (1.34) | 7.16 (7.82) | -2.00 (-6.01, 2.02) | 0.329 | -0.33 (-5.03, 4.37) | 0.891 |
| Range Hindlimb Fetlock Angle in Swing (º) | 41.15 (0.74) | 32.84 (6.18) | -8.34 (-11.06, -5.63) | <0.001 * | -6.61 (-9.89, -3.33) | <0.001 * |
| Minimum Tarsal Angle in Stance (º) | 27.70 (0.40) | 27.15 (7.76) | -0.35 (-3.32, 2.62) | 0.818 | -0.32 (-3.84, 3.19) | 0.857 |
| Maximum Tarsal Angle in Stance (º) | 63.10 (0.24) | 58.01 (9.57) | -5.07 (-7.77, -2.37) | <0.001 * | -3.41 (-6.69, -0.12) | 0.042 * |
| Range Tarsal Angle in Stance (º) | 35.41 (0.44) | 30.86 (6.08) | -4.72 (-6.81, -2.63) | <0.001 * | -3.03 (-5.49, -0.56) | 0.016 * |
| Minimum Tarsal Angle in Swing (º) | 27.73 (0.38) | 27.14 (7.59) | -0.38 (-3.51, 2.75) | 0.812 | -1.09 (-4.90, 2.71) | 0.573 |
| Maximum Tarsal Angle in Swing (º) | 77.25 (0.56) | 70.65 (11.40) | -6.53 (-9.67, -3.39) | <0.001 * | -5.14 (-8.92, -1.35) | 0.008 * |
| Range Tarsal Angle in Swing (º) | 49.53 (0.29) | 43.51 (7.20) | -6.15 (-8.91, -3.39) | <0.001 * | -3.95 (-7.26, -0.63) | 0.02 * |
| Minimum Stifle Angle in Stance (º) | -68.54 (0.65) | -65.60 (15.97) | 3.01 (-2.44, 8.46) | 0.279 | 1.86 (-4.63, 8.34) | 0.575 |
| Maximum Stifle Angle in Stance (º) | -45.12 (1.05) | -39.39 (14.56) | 5.81 (0.56, 11.06) | 0.030 * | 2.71 (-3.43, 8.85) | 0.387 |
| Range Stifle Angle in Stance (º) | 23.42 (0.46) | 26.22 (4.98) | 2.80 (0.41, 5.19) | 0.022 * | 0.58 (-2.20, 3.36) | 0.68 |
| Minimum Stifle Angle in Swing (º) | -80.72 (0.93) | -74.06 (15.27) | 6.69 (1.52, 11.86) | 0.011 * | 4.48 (-1.68, 10.64) | 0.154 |
| Maximum Stifle Angle in Swing (º) | -35.46 (1.41) | -32.70 (14.73) | 2.84 (-2.53, 8.21) | 0.300 | 0.87 (-5.44, 7.19) | 0.786 |
| Range Stifle Angle in Swing (º) | 45.26 (0.66) | 41.36 (3.87) | -3.85 (-6.14, -1.56) | 0.001 * | -3.94 (-6.70, -1.18) | 0.005 * |
| * = *p*<0.05 |  |  |  |  |  |  |
|  |  |  |  |  |  | (continued) |
|  |  |  |  |  |  |  |
|  |  |  |  |  |  |  |
| **Right hindlimb** |  | | | | | |
| *Variable* | *Mean all baselines* | *Post-stroke* | *Mean difference un-adjusted (95% CI)* | *P-value un-adjusted* | *Mean difference adjusted (95% CI)* | *P-value adjusted* |
| Stance Duration (s) | 0.43 (0.01) | 0.56 (0.11) | 0.12 (0.10, 0.15) | <0.001 * | 0.02 (-0.01, 0.04) | 0.184 |
| Swing Duration (s) | 0.34 (0.00) | 0.37 (0.03) | 0.03 (0.02, 0.04) | <0.001 * | 0.01 (-0.00, 0.02) | 0.148 |
| Stride Duration (s) | 0.77 (0.01) | 0.92 (0.12) | 0.15 (0.12, 0.18) | <0.001 * | 0.03 (0.00, 0.05) | 0.038 * |
| Ratio Stance To Stride (%) | 0.55 (0.00) | 0.60 (0.04) | 0.05 (0.03, 0.06) | <0.001 * | 0.00 (-0.01, 0.02) | 0.57 |
| Ratio Swing To Stride (%) | 0.45 (0.00) | 0.40 (0.04) | -0.05 (-0.06, -0.03) | <0.001 * | -0.00 (-0.02, 0.01) | 0.57 |
| Ratio Stance To Swing (%) | 1.27 (0.02) | 1.52 (0.26) | 0.25 (0.17, 0.32) | <0.001 * | 0.00 (-0.07, 0.08) | 0.972 |
| Hoof Lateral Deviation (cm) | 7.56 (0.23) | 5.80 (1.81) | -1.81 (-2.67, -0.95) | <0.001 * | -1.89 (-2.92, -0.86) | <0.001 * |
| Hoof Forward Swing Velocity (m/s) | 2.86 (0.03) | 2.46 (0.26) | -0.41 (-0.48, -0.33) | <0.001 * | -0.15 (-0.22, -0.07) | <0.001 * |
| Hoof Vertical Swing Velocity (m/s) | 0.22 (0.00) | 0.18 (0.08) | -0.05 (-0.08, -0.02) | 0.001 * | -0.04 (-0.08, -0.00) | 0.03 * |
| Range Hoof Height in Swing (cm) | 3.85 (0.10) | 3.24 (1.22) | -0.64 (-1.23, -0.06) | 0.032 * | -0.58 (-1.29, 0.13) | 0.109 |
| Stride Length (cm) | 97.63 (0.52) | 90.33 (6.45) | -7.44 (-9.28, -5.61) | <0.001 * | -1.53 (-3.37, 0.30) | 0.102 |
| Minimum Hindlimb Fetlock Angle Stance (º) | 2.25 (1.26) | 1.38 (8.05) | -0.85 (-4.89, 3.20) | 0.681 | 0.73 (-3.93, 5.40) | 0.758 |
| Maximum Hindlimb Fetlock Angle Stance (º) | 19.77 (1.16) | 19.52 (9.10) | -0.18 (-4.25, 3.88) | 0.930 | 0.27 (-4.56, 5.11) | 0.912 |
| Range Hindlimb Fetlock Angle in Stance (º) | 17.52 (0.25) | 18.15 (4.99) | 0.67 (-1.11, 2.45) | 0.462 | -0.29 (-2.45, 1.88) | 0.796 |
| Minimum Hindlimb Fetlock Angle in Swing (º) | -30.93 (0.91) | -24.00 (6.68) | 6.95 (2.82, 11.07) | 0.001 * | 7.03 (2.24, 11.83) | 0.004 * |
| Maximum Hindlimb Fetlock Angle in Swing (º) | 10.12 (1.36) | 8.84 (8.72) | -1.24 (-5.26, 2.77) | 0.544 | 0.43 (-4.27, 5.12) | 0.859 |
| Range Hindlimb Fetlock Angle in Swing (º) | 41.05 (0.91) | 32.84 (5.43) | -8.19 (-10.90, -5.47) | <0.001 * | -6.46 (-9.74, -3.18) | <0.001 * |
| Minimum Tarsal Angle in Stance (º) | 24.43 (0.16) | 23.85 (6.69) | -0.42 (-3.39, 2.55) | 0.781 | -0.39 (-3.91, 3.12) | 0.826 |
| Maximum Tarsal Angle in Stance (º) | 60.50 (0.48) | 55.07 (7.94) | -5.33 (-8.02, -2.63) | <0.001 * | -3.66 (-6.95, -0.38) | 0.029 * |
| Range Tarsal Angle in Stance (º) | 36.06 (0.58) | 31.22 (4.90) | -4.91 (-7.00, -2.81) | <0.001 * | -3.21 (-5.67, -0.75) | 0.011 * |
| Minimum Tarsal Angle in Swing (º) | 24.56 (0.13) | 23.56 (6.64) | -0.83 (-3.96, 2.29) | 0.602 | -1.55 (-5.36, 2.26) | 0.426 |
| Maximum Tarsal Angle in Swing (º) | 74.17 (0.32) | 66.53 (8.89) | -7.51 (-10.65, -4.37) | <0.001 * | -6.12 (-9.90, -2.33) | 0.002 * |
| Range Tarsal Angle in Swing (º) | 49.61 (0.34) | 42.97 (5.81) | -6.68 (-9.44, -3.91) | <0.001 * | -4.47 (-7.79, -1.15) | 0.008 * |
| Minimum Stifle Angle in Stance (º) | -71.01 (0.39) | -66.98 (14.54) | 4.09 (-1.37, 9.54) | 0.142 | 2.93 (-3.55, 9.41) | 0.376 |
| Maximum Stifle Angle in Stance (º) | -47.89 (0.58) | -40.12 (12.71) | 7.77 (2.52, 13.02) | 0.004 * | 4.67 (-1.47, 10.82) | 0.136 |
| Range Stifle Angle in Stance (º) | 23.12 (0.22) | 26.86 (4.81) | 3.69 (1.30, 6.08) | 0.002 * | 1.48 (-1.30, 4.26) | 0.298 |
| Minimum Stifle Angle in Swing (º) | -83.36 (0.72) | -75.97 (14.00) | 7.41 (2.24, 12.58) | 0.005 * | 5.20 (-0.96, 11.36) | 0.098 |
| Maximum Stifle Angle in Swing (º) | -38.34 (0.77) | -33.28 (13.34) | 5.06 (-0.31, 10.43) | 0.065 | 3.10 (-3.22, 9.41) | 0.336 |
| Range Stifle Angle in Swing (º) | 45.03 (0.06) | 42.69 (3.77) | -2.35 (-4.64, -0.06) | 0.044 * | -2.43 (-5.19, 0.33) | 0.084 |
| Distance Matching Limb during stance (cm) | 89.11 (0.36) | 79.66 (5.02) | -9.38 (-11.46, -7.31) | <0.001 * | -6.16 (-8.46, -3.87) | <0.001 * |
| * = *p*<0.05 |  |  |  |  |  |  |

| **Supplementary Table S9. Interaction between all limbs pre- vs post-stroke** | | |
| --- | --- | --- |
| *Outcome* | *Overall Unadjusted* | *Overall Adjusted* |
|  | *P-value interaction* | *P-value interaction** |
|  |  |  |
| Stance Duration (s) | 0.923 | 0.789 |
| Swing Duration (s) | 0.151 | 0.114 |
| Stride Duration (s) | 0.996 | 0.986 |
| Ratio Stance To Stride (%) | 0.181 | 0.084 |
| Ratio Swing To Stride (%) | 0.181 | 0.084 |
| Ratio Stance To Swing (%) | 0.287 | 0.130 |
| Hoof Lateral Deviation (cm) | 0.207 | 0.208 |
| Hoof Forward Swing Velocity (m/s) | 0.027 * | 0.002 * |
| Hoof Vertical Swing Velocity (m/s) | 0.677 | 0.678 |
| Range Hoof Height in Swing (cm) | 0.577 | 0.578 |
| Stride Length (cm) | 0.970 | 0.943 |
| Minimum Fetlock Angle Stance (º) | 0.994 | 0.994 |
| Maximum Fetlock Angle Stance (º) | 0.921 | 0.921 |
| Range Fetlock Angle in Stance (º) | 0.337 | 0.336 |
| Minimum Fetlock Angle in Swing (º) | 0.314 | 0.314 |
| Maximum Fetlock Fetlock Angle in Swing (º) | 0.975 | 0.975 |
| Range Fetlock Angle in Swing (º) | 0.01 * | 0.009 * |
| Minimum Carpal/Tarsal Angle in Stance (º) | 0.963 | 0.963 |
| Maximum Carpal/Tarsal Angle in Stance (º) | 0.085 | 0.082 |
| Range Carpal/Tarsal Angle in Stance (º) | 0.012 * | 0.011 * |
| Minimum Carpal/Tarsal Angle in Swing (º) | 0.286 | 0.287 |
| Maximum Carpal/Tarsal Angle in Swing (º) | 0.002 * | 0.002 * |
| Range Carpal/Tarsal Angle in Swing (º) | 0.141 | 0.139 |
| Minimum Elbow/Stifle Angle in Stance (º) | 0.820 | 0.818 |
| Maximum Elbow/Stifle Angle in Stance (º) | 0.789 | 0.782 |
| Range Elbow/Stifle Angle in Stance (º) | 0.052 | 0.049 * |
| Minimum Elbow/Stifle Angle in Swing (º) | 0.595 | 0.588 |
| Maximum Elbow/Stifle Angle in Swing (º) | 0.912 | 0.910 |
| Range Elbow/Stifle Angle in Swing (º) | 0.051 | 0.051 |

* = *p*<0.05

| **Supplementary Table S10. Comparison between left and right forelimbs post-stroke (mean (SD))** | | | | | | |
| --- | --- | --- | --- | --- | --- | --- |
| *Variable* | *Right forelimb* | *Left forelimb* | *Mean difference un-adjusted (95% CI)* | *P-value un-adjusted* | *Mean difference adjusted (95% CI)* | *P-value adjusted* |
| Stance Duration (s) | 0.55 (0.11) | 0.54 (0.11) | 0.01 (-0.02, 0.03) | 0.668 | 0.01 (-0.01, 0.02) | 0.526 |
| Swing Duration (s) | 0.38 (0.02) | 0.38 (0.04) | -0.00 (-0.01, 0.01) | 0.581 | -0.00 (-0.01, 0.01) | 0.559 |
| Stride Duration (s) | 0.92 (0.13) | 0.92 (0.13) | 0.00 (-0.03, 0.03) | 0.835 | 0.00 (-0.02, 0.02) | 0.746 |
| Ratio Stance To Stride (%) | 0.59 (0.04) | 0.58 (0.04) | 0.00 (-0.01, 0.02) | 0.561 | 0.00 (-0.01, 0.02) | 0.497 |
| Ratio Swing To Stride (%) | 0.41 (0.04) | 0.42 (0.04) | -0.00 (-0.02, 0.01) | 0.561 | -0.00 (-0.02, 0.01) | 0.497 |
| Ratio Stance To Swing (%) | 1.47 (0.28) | 1.44 (0.23) | 0.02 (-0.05, 0.10) | 0.567 | 0.02 (-0.04, 0.08) | 0.483 |
| Hoof Lateral Deviation (cm) | 5.88 (1.57) | 6.39 (2.04) | -0.51 (-1.37, 0.35) | 0.244 | -0.51 (-1.37, 0.35) | 0.245 |
| Hoof Forward Swing Velocity (m/s) | 2.41 (0.18) | 2.42 (0.25) | -0.01 (-0.09, 0.06) | 0.768 | -0.01 (-0.07, 0.05) | 0.712 |
| Hoof Vertical Swing Velocity (m/s) | 0.30 (0.06) | 0.32 (0.09) | -0.02 (-0.05, 0.01) | 0.262 | -0.02 (-0.05, 0.01) | 0.263 |
| Range Hoof Height in Swing (cm) | 5.71 (1.20) | 6.10 (1.89) | -0.39 (-0.97, 0.20) | 0.194 | -0.39 (-0.97, 0.20) | 0.194 |
| Stride Length (cm) | 90.52 (5.57) | 90.60 (5.41) | -0.08 (-1.91, 1.75) | 0.933 | -0.08 (-1.53, 1.38) | 0.916 |
| Minimum Forelimb Fetlock Angle Stance (º) | 5.87 (8.97) | 4.16 (9.73) | 1.71 (-2.34, 5.75) | 0.408 | 1.71 (-2.33, 5.74) | 0.407 |
| Maximum Forelimb Fetlock Angle Stance (º) | 21.57 (9.46) | 19.73 (9.89) | 1.84 (-2.23, 5.91) | 0.375 | 1.84 (-2.22, 5.91) | 0.375 |
| Range Forelimb Fetlock Angle in Stance (º) | 15.71 (4.29) | 15.57 (4.10) | 0.13 (-1.64, 1.91) | 0.883 | 0.13 (-1.64, 1.91) | 0.883 |
| Minimum Forelimb Fetlock Angle in Swing (º) | -23.78 (8.87) | -24.69 (11.23) | 0.91 (-3.22, 5.04) | 0.666 | 0.91 (-3.22, 5.04) | 0.666 |
| Maximum Forelimb Fetlock Angle in Swing (º) | 13.32 (9.07) | 12.21 (9.22) | 1.11 (-2.90, 5.12) | 0.588 | 1.11 (-2.88, 5.10) | 0.586 |
| Range Forelimb Fetlock Angle in Swing (º) | 37.10 (6.51) | 36.90 (7.81) | 0.20 (-2.51, 2.92) | 0.885 | 0.20 (-2.49, 2.89) | 0.883 |
| Minimum Carpal Angle in Stance (º) | -17.55 (4.79) | -15.47 (4.77) | -2.08 (-5.05, 0.89) | 0.170 | -2.08 (-5.05, 0.89) | 0.170 |
| Maximum Carpal Angle in Stance (º) | -5.18 (3.49) | -4.31 (4.91) | -0.86 (-3.56, 1.83) | 0.530 | -0.86 (-3.54, 1.82) | 0.528 |
| Range Carpal Angle in Stance (º) | 12.37 (3.32) | 11.16 (2.99) | 1.22 (-0.87, 3.31) | 0.254 | 1.22 (-0.87, 3.30) | 0.252 |
| Minimum Carpal Angle in Swing (º) | -76.25 (6.76) | -75.37 (6.58) | -0.88 (-4.01, 2.24) | 0.580 | -0.88 (-4.01, 2.25) | 0.580 |
| Maximum Carpal Angle in Swing (º) | -6.14 (3.72) | -5.18 (5.30) | -0.95 (-4.09, 2.19) | 0.552 | -0.95 (-4.08, 2.17) | 0.551 |
| Range Carpal Angle in Swing (º) | 70.12 (5.74) | 70.19 (6.41) | -0.07 (-2.83, 2.69) | 0.962 | -0.07 (-2.82, 2.68) | 0.962 |
| Minimum Elbow Angle in Stance (º) | 56.38 (9.20) | 57.92 (6.73) | -1.55 (-7.00, 3.90) | 0.578 | -1.55 (-6.98, 3.88) | 0.577 |
| Maximum Elbow Angle in Stance (º) | 81.20 (8.27) | 81.98 (6.87) | -0.78 (-6.03, 4.47) | 0.772 | -0.78 (-5.96, 4.41) | 0.769 |
| Range Elbow Angle in Stance (º) | 24.82 (7.63) | 24.05 (3.86) | 0.77 (-1.62, 3.16) | 0.528 | 0.77 (-1.61, 3.15) | 0.525 |
| Minimum Elbow Angle in Swing (º) | 57.95 (8.95) | 58.82 (6.46) | -0.86 (-6.03, 4.30) | 0.743 | -0.86 (-5.99, 4.26) | 0.742 |
| Maximum Elbow Angle in Swing (º) | 99.29 (8.73) | 100.21 (7.49) | -0.92 (-6.29, 4.45) | 0.736 | -0.92 (-6.25, 4.41) | 0.734 |
| Range Elbow Angle in Swing (º) | 41.34 (7.18) | 41.40 (4.61) | -0.06 (-2.35, 2.23) | 0.959 | -0.06 (-2.35, 2.23) | 0.959 |

* = *p*<0.05

| **Supplementary Table S11. Comparison between left and right hindlimbs post-stroke (mean (SD))** | | | | | | | | |
| --- | --- | --- | --- | --- | --- | --- | --- | --- |
| *Variable* | *Right hindlimb* | *Left hindlimb* | *Mean difference un-adjusted (95% CI)* | | *P-value un-adjusted* | | *Mean difference adjusted (95% CI)* | *P-value adjusted* |
| Stance Duration (s) | 0.56 (0.11) | 0.56 (0.11) | | -0.00 (-0.03, 0.03) | | 0.922 | -0.00 (-0.02, 0.02) | 0.886 |
| Swing Duration (s) | 0.37 (0.03) | 0.37 (0.04) | | -0.00 (-0.01, 0.01) | | 0.696 | -0.00 (-0.01, 0.01) | 0.679 |
| Stride Duration (s) | 0.92 (0.12) | 0.93 (0.13) | | -0.00 (-0.03, 0.03) | | 0.831 | -0.00 (-0.02, 0.02) | 0.740 |
| Ratio Stance To Stride (%) | 0.60 (0.04) | 0.60 (0.03) | | 0.00 (-0.01, 0.01) | | 0.977 | 0.00 (-0.01, 0.01) | 0.973 |
| Ratio Swing To Stride (%) | 0.40 (0.04) | 0.40 (0.03) | | -0.00 (-0.01, 0.01) | | 0.977 | -0.00 (-0.01, 0.01) | 0.973 |
| Ratio Stance To Swing (%) | 1.52 (0.26) | 1.51 (0.22) | | 0.01 (-0.07, 0.08) | | 0.824 | 0.01 (-0.05, 0.07) | 0.786 |
| Hoof Lateral Deviation (cm) | 5.80 (1.81) | 6.55 (1.97) | | -0.75 (-1.61, 0.11) | | 0.085 | -0.75 (-1.61, 0.11) | 0.086 |
| Hoof Forward Swing Velocity (m/s) | 2.46 (0.26) | 2.45 (0.27) | | 0.01 (-0.06, 0.09) | | 0.756 | 0.01 (-0.05, 0.07) | 0.698 |
| Hoof Vertical Swing Velocity (m/s) | 0.18 (0.08) | 0.21 (0.08) | | -0.04 (-0.07, -0.01) | | 0.012* | -0.04 (-0.07, -0.01) | 0.012* |
| Range Hoof Height in Swing (cm) | 3.24 (1.22) | 3.97 (1.35) | | -0.74 (-1.32, -0.15) | | 0.014* | -0.74 (-1.32, -0.15) | 0.014* |
| Stride Length (cm) | 90.33 (6.45) | 90.13 (6.06) | | 0.20 (-1.64, 2.03) | | 0.833 | 0.20 (-1.26, 1.65) | 0.791 |
| Minimum Hindlimb Fetlock Angle Stance (º) | 1.38 (8.05) | 1.81 (7.61) | | -0.44 (-4.48, 3.61) | | 0.832 | -0.44 (-4.47, 3.60) | 0.831 |
| Maximum Hindlimb Fetlock Angle Stance (º) | 19.52 (9.10) | 17.28 (7.29) | | 2.24 (-1.83, 6.31) | | 0.28 | 2.24 (-1.82, 6.30) | 0.280 |
| Range Hindlimb Fetlock Angle in Stance (º) | 18.15 (4.99) | 15.47 (4.24) | | 2.68 (0.90, 4.46) | | 0.003* | 2.68 (0.90, 4.45) | 0.003* |
| Minimum Hindlimb Fetlock Angle in Swing (º) | -24.00 (6.68) | -25.68 (8.63) | | 1.68 (-2.45, 5.80) | | 0.426 | 1.68 (-2.45, 5.80) | 0.426 |
| Maximum Hindlimb Fetlock Angle in Swing (º) | 8.84 (8.72) | 7.16 (7.82) | | 1.68 (-2.34, 5.69) | | 0.413 | 1.68 (-2.32, 5.67) | 0.410 |
| Range Hindlimb Fetlock Angle in Swing (º) | 32.84 (5.43) | 32.84 (6.18) | | -0.00 (-2.72, 2.72) | | >0.999 | -0.00 (-2.69, 2.69) | >0.999 |
| Minimum Tarsal Angle in Stance (º) | 23.85 (6.69) | 27.15 (7.76) | | -3.30 (-6.27, -0.33) | | 0.030* | -3.30 (-6.27, -0.33) | 0.030* |
| Maximum Tarsal Angle in Stance (º) | 55.07 (7.94) | 58.01 (9.57) | | -2.94 (-5.64, -0.25) | | 0.032* | -2.94 (-5.62, -0.26) | 0.031* |
| Range Tarsal Angle in Stance (º) | 31.22 (4.90) | 30.86 (6.08) | | 0.36 (-1.74, 2.45) | | 0.739 | 0.36 (-1.73, 2.44) | 0.738 |
| Minimum Tarsal Angle in Swing (º) | 23.56 (6.64) | 27.14 (7.59) | | -3.58 (-6.71, -0.45) | | 0.025* | -3.58 (-6.71, -0.45) | 0.025* |
| Maximum Tarsal Angle in Swing (º) | 66.53 (8.89) | 70.65 (11.40) | | -4.12 (-7.26, -0.98) | | 0.010* | -4.12 (-7.25, -1.00) | 0.010* |
| Range Tarsal Angle in Swing (º) | 42.97 (5.81) | 43.51 (7.20) | | -0.54 (-3.30, 2.22) | | 0.701 | -0.54 (-3.29, 2.21) | 0.700 |
| Minimum Stifle Angle in Stance (º) | -66.98 (14.54) | -65.60 (15.97) | | -1.38 (-6.83, 4.07) | | 0.620 | -1.38 (-6.81, 4.05) | 0.618 |
| Maximum Stifle Angle in Stance (º) | -40.12 (12.71) | -39.39 (14.56) | | -0.74 (-5.99, 4.51) | | 0.783 | -0.74 (-5.92, 4.45) | 0.780 |
| Range Stifle Angle in Stance (º) | 26.86 (4.81) | 26.22 (4.98) | | 0.64 (-1.75, 3.03) | | 0.598 | 0.64 (-1.73, 3.02) | 0.595 |
| Minimum Stifle Angle in Swing (º) | -75.97 (14.00) | -74.06 (15.27) | | -1.90 (-7.07, 3.26) | | 0.470 | -1.90 (-7.03, 3.22) | 0.466 |
| Maximum Stifle Angle in Swing (º) | -33.28 (13.34) | -32.70 (14.73) | | -0.58 (-5.95, 4.79) | | 0.833 | -0.58 (-5.91, 4.75) | 0.831 |
| Range Stifle Angle in Swing (º) | 42.69 (3.77) | 41.36 (3.87) | | 1.33 (-0.96, 3.61) | | 0.256 | 1.33 (-0.96, 3.61) | 0.256 |

* = *p*<0.05

| **Supplementary Table S12. Comparison of male and female global parameters and total neuroscore across baseline trials** | | | | |
| --- | --- | --- | --- | --- |
| *Variable* | *Males* | *Females* | *Difference (95% CI)* | *P-value* |
| Mean absolute velocity (SD) | 1.26 (0.17) | 1.30 (0.16) | 0.05 (-0.09, 0.18) | 0.488 |
| Mean head to T1 (SD) | 9.47 (3.85) | 10.33 (3.62) | 0.76 (-2.51, 4.03) | 0.634 |
| Total Neurological score (Median [IQR]) | 0.88 [0.42, 1.00] | 0.67 [0.38, 2.04] |  | 0.831 |
|  |  |  |  |  |

| **Supplementary Table S13. Comparison of male and female forelimb specific parameters across baseline trials (mean (SD))** | | | | | | | | |
| --- | --- | --- | --- | --- | --- | --- | --- | --- |
| *Variable* | *Male left forelimb* | *Female left forelimb* | *Mean difference (95% CI)* | *P-value* | *Male right forelimb* | *Female right forelimb* | *Mean difference (95% CI)* | *P-value* |
| Stance Duration (s) | 0.43 (0.06) | 0.38 (0.06) | -0.03 (-0.05, -0.01) | 0.007* | 0.44 (0.06) | 0.38 (0.07) | -0.03 (-0.05, -0.00) | 0.021* |
| Swing Duration (s) | 0.37 (0.02) | 0.35 (0.02) | -0.02 (-0.03, -0.00) | 0.009* | 0.37 (0.02) | 0.35 (0.02) | -0.01 (-0.03, -0.00) | 0.038* |
| Stride Duration (s) | 0.80 (0.07) | 0.73 (0.08) | -0.05 (-0.07, -0.02) | 0.002* | 0.81 (0.07) | 0.74 (0.08) | -0.04 (-0.07, -0.01) | 0.007* |
| Minimum Fetlock Angle Stance (º) | 3.81 (7.89) | 6.53 (7.89) | 0.15 (-2.98, 3.29) | 0.923 | 7.29 (6.47) | 5.19 (7.86) | 1.22 (-3.55, 6.00) | 0.615 |
| Maximum Fetlock Angle Stance (º) | 17.92 (7.87) | 23.20 (7.24) | 2.25 (-1.51, 6.02) | 0.241 | 21.02 (6.20) | 21.91 (9.89) | 3.16 (-2.19, 8.51) | 0.247 |
| Range Fetlock Angle in Stance (º) | 14.11 (3.14) | 16.67 (2.58) | 2.10 (-0.11, 4.31) | 0.062 | 13.73 (2.89) | 16.72 (3.26) | 1.93 (-0.74, 4.61) | 0.156 |
| Minimum Fetlock Angle Swing (º) | -29.57 (8.37) | -26.14 (5.53) | 1.11 (-2.06, 4.28) | 0.493 | -25.28 (8.71) | -26.93 (8.58) | 2.87 (-1.94, 7.67) | 0.243 |
| Maximum Fetlock Angle Swing (º) | 10.69 (8.66) | 15.19 (7.53) | 1.98 (-1.39, 5.34) | 0.250 | 14.18 (5.47) | 14.45 (8.09) | 3.35 (-1.59, 8.28) | 0.184 |
| Range Fetlock Angle in Swing (º) | 40.27 (5.44) | 41.33 (5.43) | 0.87 (-2.66, 4.40) | 0.629 | 39.45 (6.85) | 41.37 (5.05) | 0.48 (-3.64, 4.60) | 0.819 |
| * = *p*<0.05 | | | | | |  |  |  |

| **Supplementary Table S14. Comparison of male and female hindlimb specific parameters across baseline trials (mean (SD))** | | | | | | | | |
| --- | --- | --- | --- | --- | --- | --- | --- | --- |
| *Variable* | *Male left hindlimb* | *Female left hindlimb* | *Mean difference (95% CI)* | *P-value* | *Male right hindlimb* | *Female right hindlimb* | *Mean difference (95% CI)* | *P-value* |
| Stance Duration (s) | 0.45 (0.07) | 0.41 (0.06) | -0.01 (-0.03, 0.02) | 0.525 | 0.45 (0.07) | 0.41 (0.07) | -0.01 (-0.03, 0.02) | 0.565 |
| Swing Duration (s) | 0.35 (0.01) | 0.33 (0.03) | -0.03 (-0.05, -0.02) | <0.001* | 0.35 (0.02) | 0.33 (0.03) | -0.03 (-0.05, -0.02) | <0.001* |
| Stride Duration (s) | 0.80 (0.08) | 0.74 (0.08) | -0.04 (-0.07, -0.01) | 0.006* | 0.80 (0.08) | 0.74 (0.08) | -0.04 (-0.07, -0.01) | 0.008* |
| Minimum Fetlock Angle Stance (º) | 2.65 (5.41) | 1.67 (7.16) | -2.86 (-7.63, 1.92) | 0.241 | 1.17 (6.81) | 3.28 (6.34) | -2.79 (-7.56, 1.99) | 0.252 |
| Maximum Fetlock Angle Stance (º) | 18.92 (6.29) | 19.22 (8.38) | 0.76 (-4.58, 6.11) | 0.779 | 18.06 (8.54) | 21.35 (6.74) | 1.40 (-3.95, 6.75) | 0.608 |
| Range Fetlock Angle in Stance (º) | 16.27 (4.58) | 17.55 (4.54) | 3.62 (0.95, 6.29) | 0.008* | 16.89 (4.16) | 18.07 (4.09) | 4.19 (1.52, 6.86) | 0.002* |
| Minimum Fetlock Angle Swing (º) | -31.79 (5.07) | -32.26 (7.41) | -3.06 (-7.86, 1.75) | 0.213 | -32.31 (5.99) | -29.59 (5.06) | -1.98 (-6.79, 2.83) | 0.420 |
| Maximum Fetlock Angle Swing (º) | 8.74 (6.02) | 9.58 (7.37) | -1.81 (-6.75, 3.13) | 0.473 | 8.23 (7.55) | 11.93 (6.91) | -0.89 (-5.82, 4.05) | 0.725 |
| Range Fetlock Angle in Swing (º) | 40.53 (6.57) | 41.83 (4.60) | 1.25 (-2.87, 5.37) | 0.553 | 40.54 (7.49) | 41.51 (5.05) | 1.09 (-3.03, 5.21) | 0.603 |
| * = *p*<0.05 | | | |  |  |  |  |  |

| **Supplementary Table S15. Comparison of male and female global parameters and total neuroscore post-stroke** | | | | |
| --- | --- | --- | --- | --- |
| *Variable* | *Males* | *Females* | *Difference (95% CI)* | *P-value* |
| Mean absolute velocity (SD) | 0.98 (0.17) | 1.01 (0.19) | 0.03 (-0.12, 0.18) | 0.675 |
| Mean head to T1 (SD) | -0.63 (7.70) | 4.02 (6.33) | 4.04 (-1.27, 9.35) | 0.129 |
| Total Neurological score (Median [IQR]) | 8.88 [5.00, 12.00] | 7.50 [5.00, 8.88] |  | 0.619 |

| **Supplementary Table S16. Comparison of male and female forelimb specific parameters post-stroke (mean (SD))** | | | | | | | | |
| --- | --- | --- | --- | --- | --- | --- | --- | --- |
| *Variable* | *Male left forelimb* | *Female left forelimb* | *Mean difference (95% CI)* | *P-value* | *Male right forelimb* | *Female right forelimb* | *Mean difference (95% CI)* | *P-value* |
| Stance Duration (s) | 0.57 (0.11) | 0.52 (0.11) | -0.03 (-0.06, -0.01) | 0.019* | 0.58 (0.11) | 0.52 (0.11) | -0.03 (-0.05, 0.00) | 0.092 |
| Swing Duration (s) | 0.39 (0.05) | 0.36 (0.03) | -0.02 (-0.04, -0.01) | 0.002* | 0.39 (0.02) | 0.36 (0.02) | -0.03 (-0.05, -0.01) | 0.004* |
| Stride Duration (s) | 0.96 (0.13) | 0.88 (0.12) | -0.06 (-0.09, -0.02) | 0.001* | 0.97 (0.13) | 0.88 (0.11) | -0.05 (-0.09, -0.02) | 0.002* |
| Minimum Fetlock Angle Stance (º) | 1.06 (10.97) | 7.26 (7.52) | -0.40 (-3.74, 2.93) | 0.813 | 8.55 (6.56) | 3.18 (10.46) | 1.30 (-4.46, 7.07) | 0.658 |
| Maximum Fetlock Angle Stance (º) | 15.74 (11.15) | 23.72 (6.74) | 1.34 (-2.59, 5.26) | 0.504 | 22.79 (6.72) | 20.35 (11.77) | 3.18 (-2.98, 9.34) | 0.312 |
| Range Fetlock Angle in Stance (º) | 14.68 (4.10) | 16.47 (4.07) | 1.74 (-0.33, 3.81) | 0.099 | 14.25 (3.65) | 17.17 (4.53) | 1.87 (-1.08, 4.83) | 0.214 |
| Minimum Fetlock Angle Swing (º) | -28.89 (12.65) | -20.49 (8.06) | 0.15 (-3.40, 3.70) | 0.935 | -21.99 (6.80) | -25.57 (10.55) | 1.06 (-5.04, 7.15) | 0.734 |
| Maximum Fetlock Angle Swing (º) | 8.75 (9.89) | 15.68 (7.33) | 1.10 (-2.34, 4.53) | 0.532 | 14.34 (6.52) | 12.31 (11.28) | 2.21 (-3.64, 8.06) | 0.459 |
| Range Fetlock Angle in Swing (º) | 37.64 (9.40) | 36.16 (6.16) | 0.95 (-2.34, 4.23) | 0.571 | 36.33 (6.52) | 37.87 (6.69) | 1.15 (-3.42, 5.72) | 0.621 |
| * = *p*<0.05 | | |  |  |  |  |  |  |

| **Supplementary Table S17. Comparison of male and female hindlimb specific parameters post-stroke (mean (SD))** | | | | | | | | |
| --- | --- | --- | --- | --- | --- | --- | --- | --- |
| *Variable* | *Male left hindlimb* | *Female left hindlimb* | *Mean difference (95% CI)* | *P-value* | *Male right hindlimb* | *Female right hindlimb* | *Mean difference (95% CI)* | *P-value* |
| Stance Duration (s) | 0.59 (0.11) | 0.53 (0.10) | -0.01 (-0.04, 0.01) | 0.315 | 0.57 (0.11) | 0.54 (0.11) | -0.02 (-0.05, 0.01) | 0.274 |
| Swing Duration (s) | 0.39 (0.04) | 0.35 (0.03) | -0.03 (-0.05, -0.01) | <0.001* | 0.38 (0.03) | 0.35 (0.03) | -0.04 (-0.05, -0.02) | <0.001* |
| Stride Duration (s) | 0.97 (0.14) | 0.88 (0.12) | -0.05 (-0.08, -0.01) | 0.005* | 0.95 (0.12) | 0.90 (0.12) | -0.05 (-0.08, -0.02) | 0.003* |
| Minimum Fetlock Angle Stance (º) | 3.95 (5.63) | -0.32 (8.91) | -2.75 (-8.51, 3.02) | 0.350 | -0.08 (7.95) | 2.83 (8.22) | -3.19 (-8.95, 2.58) | 0.279 |
| Maximum Fetlock Angle Stance (º) | 18.83 (4.92) | 15.73 (9.03) | -1.11 (-7.27, 5.05) | 0.724 | 17.88 (9.15) | 21.17 (9.14) | 1.13 (-5.03, 7.29) | 0.719 |
| Range Fetlock Angle in Stance (º) | 14.88 (3.97) | 16.06 (4.59) | 1.64 (-1.32, 4.60) | 0.278 | 17.96 (4.50) | 18.33 (5.63) | 4.32 (1.36, 7.28) | 0.004* |
| Minimum Fetlock Angle Swing (º) | -22.66 (7.04) | -28.69 (9.29) | -0.84 (-6.94, 5.25) | 0.787 | -25.15 (8.40) | -22.85 (4.45) | 0.84 (-5.26, 6.93) | 0.788 |
| Maximum Fetlock Angle Swing (º) | 8.45 (5.73) | 5.87 (9.57) | -3.95 (-9.80, 1.89) | 0.185 | 7.32 (9.39) | 10.35 (8.12) | -2.28 (-8.13, 3.57) | 0.445 |
| Range Fetlock Angle in Swing (º) | 31.11 (6.28) | 34.57 (5.81) | -3.11 (-7.68, 1.45) | 0.181 | 32.48 (5.60) | 33.20 (5.48) | -3.11 (-7.68, 1.45) | 0.181 |
| * = *p*<0.05 | | |  |  |  |  |  |  |

|  |  |
| --- | --- |
| **Supplementary Table S18. Midline shift measurements** |  |
| *Animal ID* | *Midline Shift (mm)* |
| 1 | 3.76 |
| 2 | 3.77 |
| 3 | 1.02 |
| 4 | 1.87 |
| 5 | 3.99 |
| 6 | 2.64 |
| 7 | 1.98 |
| 8 | 2.06 |
| 9 | 1.41 |
| 10 | 2.11 |
| 11 | 1.16 |
| 12 | 1.05 |
| 13 | 4.31 |
| 14 | 4.95 |
| 15 | 3.31 |
| 16 | 2.35 |
| 17 | 2.77 |
| 18 | 4.02 |
| 19 | 4.98 |
| 20 | 3.35 |

| **Supplementary Table S19. Comparison between global parameters and neuroscore post-stroke for infarcts <6 cm^3^ and >18 cm^3^** | | | | |
| --- | --- | --- | --- | --- |
| *Variable* | *Infarct volume < 6cm^3^* | *Infarct volume >18 cm^3^* | *Mean difference (95% CI)* | *P-value* |
| Infarct volume (Median [IQR]) | 1.99 [0.90, 3.19] | 21.99 [19.33, 25.46] |  | <0.001* |
| Mean absolute velocity (SD) | 1.05 (0.18) | 0.92 (0.16) | -0.12 (-0.32, 0.07) | 0.202 |
| Mean head to T1 (SD) | 3.83 (6.26) | -3.04 (10.05) | -4.89 (-12.73, 2.95) | 0.206 |
| Total Neurological score (Median [IQR]) | 6.25 [4.25, 11.5] | 9.50 [7.50, 12.00] |  | 0.256 |
| * = *p*<0.05 |  |  |  |  |

| **Supplementary Table S20. Comparison between forelimb specific parameters post-stroke for infarcts <6 cm^3^ and >18 cm^3^ (mean (SD))** | | | | | | | | |
| --- | --- | --- | --- | --- | --- | --- | --- | --- |
|  | **Left forelimb** |  |  |  | **Right forelimb** |  |  |  |
| *Variable* | *Infarct volume <6 cm^3^* | *Infarct volume >18 cm^3^* | *Mean difference (95% CI)* | *P-value* | *Infarct volume <6 cm^3^* | *Infarct volume >18 cm^3^* | *Mean difference (95% CI)* | *P-value* |
| Stance duration | 0.50 (0.10) | 0.61 (0.06) | 0.04 (0.01, 0.06) | 0.018* | 0.52 (0.12) | 0.60 (0.07) | 0.05 (0.01, 0.08) | 0.006* |
| Swing duration | 0.37 (0.05) | 0.39 (0.03) | -0.00 (-0.03, 0.02) | 0.931 | 0.37 (0.02) | 0.39 (0.03) | -0.00 (-0.03, 0.02) | 0.822 |
| Stride duration | 0.88 (0.13) | 1.00 (0.08) | 0.03 (-0.01, 0.08) | 0.126 | 0.89 (0.14) | 0.99 (0.07) | 0.04 (-0.00, 0.09) | 0.065 |
| Minimum fetlock stance | 3.78 (11.93) | 4.87 (5.10) | -1.75 (-6.38, 2.88) | 0.458 | 6.62 (9.71) | 2.43 (10.12) | -0.23 (-7.35, 6.88) | 0.949 |
| Maximum fetlock stance | 18.62 (11.92) | 22.04 (4.93) | -1.45 (-6.99, 4.09) | 0.608 | 21.97 (11.14) | 19.19 (7.41) | 0.36 (-7.45, 8.16) | 0.929 |
| Range Fetlock stance | 14.84 (4.52) | 17.17 (4.23) | 0.30 (-2.70, 3.31) | 0.843 | 15.36 (4.65) | 16.76 (4.69) | 0.59 (-3.25, 4.43) | 0.764 |
| Minimum fetlock swing | -25.63 (12.70) | -19.06 (9.90) | 0.54 (-4.41, 5.48) | 0.832 | -23.76 (10.19) | -21.86 (7.94) | 1.24 (-6.32, 8.80) | 0.748 |
| Maximum fetlock swing | 12.76 (10.94) | 13.36 (5.01) | -3.20 (-7.89, 1.50) | 0.182 | 14.23 (10.34) | 10.69 (8.52) | -2.76 (-9.98, 4.46) | 0.453 |
| Range fetlock swing | 38.39 (8.47) | 32.42 (6.86) | -3.73 (-8.08, 0.61) | 0.092 | 37.99 (7.06) | 32.54 (5.70) | -4.00 (-9.65, 1.65) | 0.165 |
| * = *p*<0.05 |  |  |  |  |  |  |  |  |

| **Supplementary Table S21. Comparison between hindlimb specific parameters post-stroke for infarcts <6 cm^3^ and >18 cm^3^ (mean (SD))** | | | | | | | | |
| --- | --- | --- | --- | --- | --- | --- | --- | --- |
|  | **Left hindlimb** |  |  |  | **Right hindlimb** |  |  |  |
| *Variable* | *Infarct volume <6 cm^3^* | *Infarct volume >18 cm^3^* | *Mean difference (95% CI)* | *P-value* | *Infarct volume <6 cm^3^* | *Infarct volume >18 cm^3^* | *Mean difference (95% CI)* | *P-value* |
| Stance duration | 0.53 (0.11) | 0.62 (0.07) | 0.06 (0.02, 0.09) | 0.001* | 0.52 (0.10) | 0.63 (0.10) | 0.05 (0.02, 0.09) | 0.002* |
| Swing duration | 0.37 (0.04) | 0.38 (0.03) | -0.01 (-0.04, 0.02) | 0.463 | 0.36 (0.04) | 0.38 (0.02) | -0.01 (-0.04, 0.02) | 0.443 |
| Stride duration | 0.89 (0.14) | 1.00 (0.07) | 0.05 (0.00, 0.09) | 0.050 | 0.88 (0.12) | 1.01 (0.09) | 0.04 (-0.00, 0.09) | 0.078 |
| Minimum fetlock stance | 2.90 (8.81) | -2.80 (2.59) | -4.33 (-11.45, 2.79) | 0.233 | 1.51 (9.29) | -1.46 (4.13) | -5.04 (-12.16, 2.08) | 0.165 |
| Maximum fetlock stance | 18.30 (8.55) | 14.15 (3.08) | -3.66 (-11.46, 4.15) | 0.359 | 20.34 (10.61) | 16.17 (5.48) | -1.63 (-9.43, 6.18) | 0.683 |
| Range Fetlock stance | 15.41 (4.87) | 16.95 (1.68) | 0.67 (-3.17, 4.51) | 0.731 | 18.83 (5.60) | 17.63 (3.90) | 3.41 (-0.43, 7.25) | 0.082 |
| Minimum fetlock swing | -24.91 (9.41) | -31.49 (6.04) | -2.03 (-9.60, 5.53) | 0.598 | -23.95 (7.54) | -26.63 (2.77) | -0.09 (-7.65, 7.47) | 0.981 |
| Maximum fetlock swing | 9.10 (8.84) | 1.83 (2.50) | -8.82 (-16.04, -1.60) | 0.017* | 10.20 (10.16) | 4.74 (4.83) | -7.27 (-14.49, -0.05) | 0.048* |
| Range fetlock swing | 34.02 (6.18) | 33.31 (6.79) | -6.79 (-12.44, -1.14) | 0.019* | 34.15 (5.90) | 31.37 (4.56) | -7.18 (-12.83, -1.53) | 0.013* |
| * = *p*<0.05 |  |  |  |  |  |  |  |  |
